# C-phycocyanin reinforces autophagy to block pulmonary fibrogenesis by inhibiting lncIAPF biogenesis

**DOI:** 10.1007/s12272-024-01508-y

**Published:** 2024-07-22

**Authors:** Wenjie Hu, Yujie Wang, Huiling Yang, Leiming Zhang, Bo Liu, Yunxia Ji, Xiaodong Song, Changjun Lv, Songzi Zhang

**Affiliations:** 1 Department of Cellular and Genetic Medicine, Binzhou Medical University, Yantai, 264003 China; 2 Department of Respiratory and Critical Care Medicine, Binzhou Medical University Hospital, Binzhou Medical University, Binzhou, 256603 China; 3 Department of Integrated Traditional Chinese and Western Medicine, Binzhou Medical University, Yantai, 264003 China; 4 Department of Biomedical Science, College of Life Sciences, CHA University, Songnam, 13496 Korea

**Keywords:** Pulmonary fibrosis, C-phycocyanin, lncIAPF, ATF3, Smad3, Autophagy

## Abstract

Pulmonary fibrosis is a chronic and irreversible progressive lung disease caused by various factors, such as age and environmental pollution. With countries stepping into an aging society and the seriousness of environmental pollution caused by global industrialization, the incidence of pulmonary fibrosis is annually increasing. However, no effective drug is available for pulmonary fibrosis treatment. C-phycocyanin (C-PC), extracted from blue-green algae, has good water solubility and antioxidation. This study elucidated that C-PC reinforces autophagy to block pulmonary fibrogenesis by inhibiting long noncoding RNA (lncRNA) biogenesis in vivo and in vitro. Cleavage under targets and release using nuclease (CUT & RUN)-PCR, co-immunoprecipitation (Co-IP), and nuclear–cytoplasmic separation experiments clarified that C-PC blocked the nuclear translocation of activating transcription factor 3 (ATF3) to prevent the binding between ATF3 and transcription factor Smad3, thereby hindering lncIAPF transcription. Human antigen R (HuR) truncation experiment and RNA binding protein immunoprecipitation (RIP) were then performed to identify the binding domain with lncIAPF in the 244–322 aa of HuR. lncIAPF exerted its profibrogenic function through the binding protein HuR, a negative regulator of autophagy. In summary, C-PC promoted autophagy via down-regulating the lncIAPF–HuR-mediated signal pathway to alleviate pulmonary fibrosis, showing its potential as a drug for treating pulmonary fibrosis. Exploring how C-PC interacts with biological molecules will help us understand the mechanism of this drug and provide valuable target genes to design new drugs.

**Supplementary Information:**

The online version contains supplementary material available at 10.1007/s12272-024-01508-y.

## Introduction

Pulmonary fibrosis is a chronic and irreversible progressive lung disease caused by various factors, such as age, environmental pollution, virus infection, chemical drugs, and some autoimmune diseases (Scholand and Wells 2022). Its main clinical manifestations are pulmonary function injury, cough, and dyspnea, which eventually cause the patients to die from respiratory failure (Gu et al. 2021). Among the types of pulmonary fibrosis, idiopathic pulmonary fibrosis (IPF) is the most common with very high morbidity and mortality. More than half of the patients die 2–3 years after diagnosis, and the 5-year survival rate is less than 30% (Richeldi et al. 2017; Moss et al. 2022). The incidence of pulmonary fibrosis is annually increasing due to two main reasons: the first is that with the average life span extending, more and more countries step into an aging society (Han et al. 2023). The second is that with the pace of global industrialization, the environment that we rely on has been heavily damaged. Pollution has become serious (Yue et al. 2023). Pirfenidone and nidanib are the two drugs approved by the Food and Drug Administration for IPF treatment; however, they only delay the progress of pulmonary fibrosis and cannot improve lung function and prolong patient survival time (Raghu and Selman 2015; Kinoshita and Goto 2019; Savin et al. 2022). Hence, no effective drug is available for pulmonary fibrosis treatment. One of the reasons for this situation is that the requirements for drug target design cannot be met because the mechanism of pulmonary fibrosis remains unclear. Therefore, exploring the mechanism of pulmonary fibrosis and developing effective intervention drugs against this disease is of social significance.

LncRNA is a kind of RNA transcript with a length of more than 200 nucleotides; they usually cannot encode protein (Hangauer et al. 2013; Kopp and Mendell 2018). LncRNAs can control biological processes, including endocytic trafficking, cell cycle, immune response, and cell fate such as proliferation, migration, differentiation, and death, by interacting with proteins, DNAs, and RNAs (Herman et al. 2022). To date, numerous lncRNAs have been identified as molecular players involved in the occurrence and development of diseases (Delás et al. 2019). For example, lncRNA metastasis-related lung adenocarcinoma transcript 1 is up-regulated in osteoarthritis (OA) chondrocytes; its down-regulation can inhibit cell proliferation and promote cell apoptosis and ECM degradation, thus inhibiting the progress of OA (Zhang et al. 2019a, b). LncRNA MAMDC2 antisense 1 plays an important role in virus infection. It interacts with RNA binding protein heat shock protein 90α, which promotes the nuclear transport of virus envelope protein VP16 and enhances the susceptibility of HSV-1 and the expression of HSV-1 immediately early gene in human cells (Wang et al. 2020a, b). However, only a few lncRNAs have been functionally studied as the target for drug action. A recent study demonstrated that as a fibrogenic factor, lncIAPF can form an RNA–protein complex with HuR to accelerate pulmonary fibrosis by blocking autophagy (Zhang et al. 2022). Whether lncIAPF can serve as a drug target needs further exploration.

Owing to the unique environment of the ocean that creates many active substances with special structures and functions, marine compounds have received increasing attention. For example, bryostatin-1 extracted from bryozoan *Bugula neritina* can block the infiltration of neutrophils into the graft by activating EC messenger PKC and reduce histological and ultrastructural damage, thus protecting the kidney from ischemia–reperfusion injury and reducing allograft rejection (Becker et al. 2022). Another marine compound, didemnin B, was applied for the treatment of nonalcoholic obesity liver disease (NAFLD) in western diet-induced obese mice and found to reduce hepatic steatosis and hepatocyte vesiculation and improve NAFLD histopathology, glucose homeostasis, and dyslipidemia (Wilson et al. 2020). C-phycocyanin (C-PC), extracted from *Spirulina platensis*, is also a marine compound. Here, C represents the cyanobacteria biological type. This compound is composed of two subunits, α and β, and has good water solubility, antioxidation, and spontaneous red fluorescence characteristics (Zheng et al. 2019; Ji et al. 2020). C-PC can promote the balance of mitochondrion division and fusion and prevent myocardial cells from intrinsic apoptosis by inhibiting the phosphorylation of extracellular signal-regulated kinase 1/2 and C-jun N-terminal protein kinase (Gao et al. 2019). However, whether C-PC can alleviate pulmonary fibrosis by regulating lncRNA has not been reported yet. This study aimed to illustrate the anti-pulmonary fibrosis function and lncIAPF-mediated mechanism of C-PC and provide a potential therapeutic approach for pulmonary fibrosis.

## Materials and methods

### Animal model and ethical statement

All animal experiments were approved by the Animal Experimental Ethics Committee of Binzhou Medical University (No. 2021-355). C57BL/6 mice aged 6–8 weeks were purchased from Nanjing University Model Animal Research Center (Nanjing, China). According to the experimental requirements, mice were divided into different groups (10 mice in each group). Mice in the bleomycin (BLM, Nippon Chemical Co., Ltd., Japan) group were sprayed 5 mg/kg BLM into the trachea by using Penn-century microplayer (Penncentury, Inc., Wyndmoor, PA, USA), while the control group mice were sprayed with the same volume of physiological saline. C-PC (Aladdin, Shanghai, China) treatment group was given 25, 50, and 100 mg/kg of C-PC aqueous solution respectively after BLM modeling. In the lncIAPF/NC group, 1.0 × 10^12^ vg/mL adenovirus-lncIAPF/NC was sprayed with Penn-Century MicroSprayer respectively, and the sham operation group was sprayed with the same volume of normal saline. Lung tissue was collected on the 28th day after BLM modeling. After anesthesia, the right lobe lung tissue of mice was taken and quickly stored in liquid nitrogen for extracting tissue protein and RNA; the left lung tissue was perfused with normal saline and then fixed with 4% paraformaldehyde for pathological examination.

### Cell culture

Human fetal lung fibroblasts (MRC-5 cell line) were purchased from the American Type Culture Collection. Cells were cultured in a Thermo Scientific incubator with 5% carbon dioxide at 37 °C. The cell culture system is Minimum Essential Medium (MEM, Gibco, CA, USA), with 10% fetal bovine serum (FBS, Gibco, CA, USA) and 1% 100 × penicillin/streptomycin/amphotericin B solution (SparkJade, Shandong, China).

### Cell viability assay

3 × 10^3^ MRC-5 cells/well were cultured in the 96-well plate. Different concentrations of C-PC (50, 100, 200, 400, and 800 μg/mL) were added and cultured for 48 h. Then, the fresh medium containing 10% CCK-8 reagent (Meilun Biotechnology, Dalian, China) was added. After 2 h of culture, the absorbance was detected at 450 nm wavelength by a microplate reader. The cell viability was calculated according to the cell viability formula provided by the kit.

### Wound healing assay

After 4 × 10^3^ MRC-5 cells were cultured in a 96-well plate for 24 h, the cells were divided into different groups according to the experimental requirement. When cells overgrew one well of 96-well, the 96-well plate was put into an IncuCyte S3 scratch device (Reese, Sartor, MI, USA) to draw the dividing line. Cell residues were washed with PBS three times. 100 μL serum-free medium was added. Then the 96-well plate was put into the IncuCyte S3 instrument for observation. Images were obtained for analysis by using Image-Pro Plus software.

### Quantitative real-time polymerase chain reaction (qRT-PCR)

RNA was extracted from tissue or cell samples with Trizol reagent (Aikerui, Hunan, China). Taking 1000 ng RNA (20 μL system), using a one-step reverse transcription kit Evo M-MLV RT premix (Aikerui), the extracted RNA was reverse transcribed into cDNA at 37 °C, 15 min, 85 °C and 5 s. The relative expression of each target gene was normalized by GAPDH (Aikerui). The total PCR reaction system is 20 μL, and the conditions are as follows: pre-denaturation at 95 °C for 30 s; then 45 cycles at 95 °C for 5 s and 60 °C for 20 s. using the following primers: GAPDH, Forward Primer 5′ to 3′, GCACCGTCAAGGCTGAGAAC, Reverse Primer 5′ to 3′, TGGTGAAGACGCCAGTGGA; lncIAPF, Forward Primer 5′ to 3′, GCGGTAGCCTTCTCTGAACTG, Reverse Primer 5′ to 3′, GTTGCATAACCTGACCTGCC; ATF3, Forward Primer 5′ to 3′, CCATCCAGAACAAGCACCTCT, Reverse Primer 5′ to 3′, GGCACTCCGTCTTCTCCTTC.

### Western blot

Total proteins of MRC-5 cells and mouse lung tissues were extracted with RIPA lysis buffer (SparkJade, Shandong, China) containing 1% (v/v) PMSF (SparkJade). After lysis for 30 min, the supernatant was obtained by centrifugation at 12,000 rpm for 20 min. The concentration of total protein was determined by the BCA protein detection kit (Coolaber, Beijing, China). 20 μg protein in each group was separated by SDS-PAGE, then transferred to polyvinylidene fluoride (PVDF, Merck KGaA, New Jersey, Germany) under the conditions of ice bath and 200 mA. The PVDF-contained protein was incubated with 5% skim milk for 2 h at room temperature, washed with 1 × TBST, and then incubated with the corresponding primary antibody at 4 °C. The next day, it was washed with 1 × TBST, and incubated with the corresponding HRP-coupled secondary antibody of the primary antibody for 1 h at room temperature, and then was washed with 1 × TBST. Finally, the target band was detected by ECL super (SparkJade) and Tanon5200 multi-chemiluminescence imaging system (Tanon, Shanghai, China). The antibodies used in this study are as follows: rabbit anti-GAPDH (1:8000, Affinity Bioscience, WV, USA); rabbit anti-Collagen I (1:2000, Proteintech, Wuhan, China); rabbit anti-Collagen III (1:1000, Proteintech); rabbit anti-FAP1 (1:1000, Cell Signal Technology, MA, USA); rabbit anti-Vimentin, (1:1000, Cell Signal Technology); rabbit anti-alpha-SMA (1:1000, Affinity Bioscience); rabbit anti-S100A4 (1:1000, Cell Signal Technology); mouse anti-Smad3 (1:8000, Proteintech); mouse anti-ATF3 (1:2000, Boster Biological Technology, Wuhan, China); rabbit anti-HuR (1:10,000, Proteintech); goat anti-mouse HRP-conjugated IgG (1:8000, Affinity Bioscience) and goat anti-rabbit HRP-conjugated IgG (1:8000, Affinity Bioscience).

### Cleavage under targets and release using nuclease (CUT & RUN)-qPCR

Hyperactive pG-MNase CUT & RUN Assay Kit for PCR/qPCR (Vazyme, Nanjing, China) was used and the kit instructions were followed. In short, 2 × 10^5^ living cells were collected and were transferred to the EP tube of ConA Beads Pro, and incubated at room temperature for 10 min after mixing. After centrifugation, the precipitant was treated with the precooled antibody buffer and the antibodies of rabbit IgG (5 μg, Cell Signal Technology) and rabbit anti-ATF3 (5 μg, Abcam, Cambridge, UK) were added, respectively. The mixtures were incubated for 2 h at room temperature. After centrifugation again, the precipitant was washed by the dig-wash buffer 4 times, treated with PG-Mnase Enzyme, and then centrifuged to gain the new precipitant. The new precipitant was treated with dig-wash buffer twice, treated with CaCl_2_ premix to fragment, and incubated on ice for 1 h. A stop buffer was added to the mixture containing the new precipitant and CaCl_2_ premix. The mixture was put into a thermostat water bath at 37 °C for 30 min. Finally, the products were collected and quantitatively detected by qPCR.

### Immunofluorescence analysis

The cells were transferred to cell slides for culture. After treatment with C-PC or TGFβ1, the culture medium was discarded. Cells were washed with 1 × PBS and fixed with 4% cell fixation fluid for 30 min. The fixed cells were washed with 1 × PBS and perforated with 0.1% TritonX-100 (SparkJade) for 10 min. Then the perforated cells were sealed with goat serum (SparkJade) at room temperature and incubated with rabbit anti-ATF3 (1:200, Abcam, Cambridge, UK) antibody at 4 °C. The next day, cells were treated with goat anti-rabbit IgG (h + L) fluor488-conjugated antibody (1:300, Affinity Bioscience) for 1 h at room temperature. Then the cells were treated with DAPI (1:400, Suolaibao, Beijing, China) for 5 min in the dark and washed with 1 × PBS. Finally, the fluorescence was detected by a laser confocal microscope (Leica, Hesse, Germany).

### Nuclear–cytoplasmic fractionation

The cytoplasm and nuclear protein were separated by NE-PER Nuclear and Cytoplasmic Extraction Reagents kit (Thermo Scientific, MA, USA). Briefly, the cells were collected and added with the precooled CER I and CER II. The whole process was carried out on ice. After centrifugation, the supernatant was cytoplasmic protein. The precipitation was added with precooled NER, resuspended, and centrifuged. The supernatant was the nuclear protein. The protein samples were detected by SDS-PAGE.

### RNA immunoprecipitation (RIP) assay

RIP was performed by using the RNA Immunoprecipitation kit (Geneseed, Guangzhou, China). Briefly, 1 × 10^7^ cells were collected and lysed on ice for 10 min. After centrifugation, 100 µL supernatant was taken to a new centrifuge tube and pretreated with magnetic beads to decrease the non-specific combination. The IgG antibody and DYKDDDDK label antibody were treated with magnetic beads at 4 °C for 2 h to make antibodies combine with the beads. These magnetic beads that bound with rabbit anti-DYKDDDDK label (5 μg, Proteintech) and the antibody of rabbit IgG (5 μg, Cell Signal Technology) were added into supernatant and stood at 4 °C overnight. The next day, the magnetic beads were collected and washed. Finally, the target gene bound to the magnetic beads was eluted, purified, and analyzed by qRT-PCR.

### Co-immunoprecipitation (Co-IP)

Co-immunoprecipitation kit (Absin, Shanghai, China) was used according to the instructions of the kit. First, 1 × 10^6^ cells were collected, treated with precooled lysis buffer for 5 min, and ultrasonically crushed on ice 3 times. After centrifugation, the supernatant was treated with 5 μL ProteinA/G and incubated at 4 °C for 1 h. After centrifugation again, the antibody of rabbit IgG (5 μg, Cell Signal Technology) and rabbit anti-ATF3 (5 μg, Abcam) were added respectively into the supernatant and incubated at 4 °C overnight. The next day, the supernatant was added with 5 μL ProteinA/G and incubated at 4 °C for 3 h. After centrifugation, the precipitant was washed 3 times with washing buffer, treated with 1 × SDS to collect protein samples, and tested.

### Histological evaluation

The lung tissue samples of mice were collected and fixed with 4% paraformaldehyde at 4 °C for 48 h. After dehydration with graded alcohol, the tissues were soaked in paraffin at 57 °C overnight and embedded in paraffin. Then, the paraffin tissue blocks were cut into 5 μM sections by microtomes (Leica, Hesse, Germany). The sections were stained with hematoxylin and eosin or a Masson trichrome staining kit. The images were captured and analyzed under the inverted microscope.

### Dual-luciferase reporter assay

Cells were inoculated into 24-well plates until the fusion rate reached 60% before transfection. The constructed lncIAPF pGL4.10-CXCL10 firefly luciferase plasmid, pRL-CMV sea kidney luciferase plasmid (internal reference), and ATF3 plasmid were co-transfected into cells, and drugs were added into cells according to different groups. Then the cell lysates were extracted. The luciferase activities of fireflies and sea kidneys were detected by a dual luciferase report detection system (Promega, WI, USA) and a Spark multifunctional micropore detector (TECAN, Scona Province, Switzerland).

### FVC testing

After the mice were anesthetized intraperitoneally, a small incision was made in the trachea of the mice, and a plastic catheter was inserted into the small incision. Then, a plastic catheter was connected to the pulmonary function testing system (DSI Buxco, MN, USA) after the insertion of a metal cannula within the small incision. The mice were mechanically ventilated with a respiratory rate of 150 breaths/min, tidal volume of 10 mL/kg, and PEEP 3 cm H_2_O. The negative pressure-driven forced expiratory maneuver was applied. The mouse lung was inflated to a pressure of 30 cm H_2_O for 2 s, then the airway was connected to the negative pressure reservoir (− 50 cm H_2_O) for 2 s. FVC was calculated directly from the flow cycle generated during lung deflation.

### MicroCT measurement

The MicroCT imaging system for small animals (PerkinElmer, MA, USA) was warm-up and the parameters were adjusted in advance. After the mice were subjected to intraperitoneal anesthesia, they were positioned flat on the MicroCT machine. The X-ray parameters were adjusted to 90 kV and 88 μA. The resolution of CT images was 36 mm FOV, with an exposure time of 4 min. Two-dimensional tomographic images were obtained by using imaging software. Finally, the MicroCT images were taken and outputted.

### Statistical analysis

Data were expressed as the means ± standard deviation (SD) and analyzed using the GraphPad Prism statistic software program. Data were presented as the mean ± SD of at least three independent experiments. Differences between groups were assessed by a two-sided Student’s t-test. Statistical significance was considered at p < 0.05.

## Results

### C-PC blocked pulmonary fibrogenesis in TGFβ1-induced MRC-5 cells

**Fig. 1:**
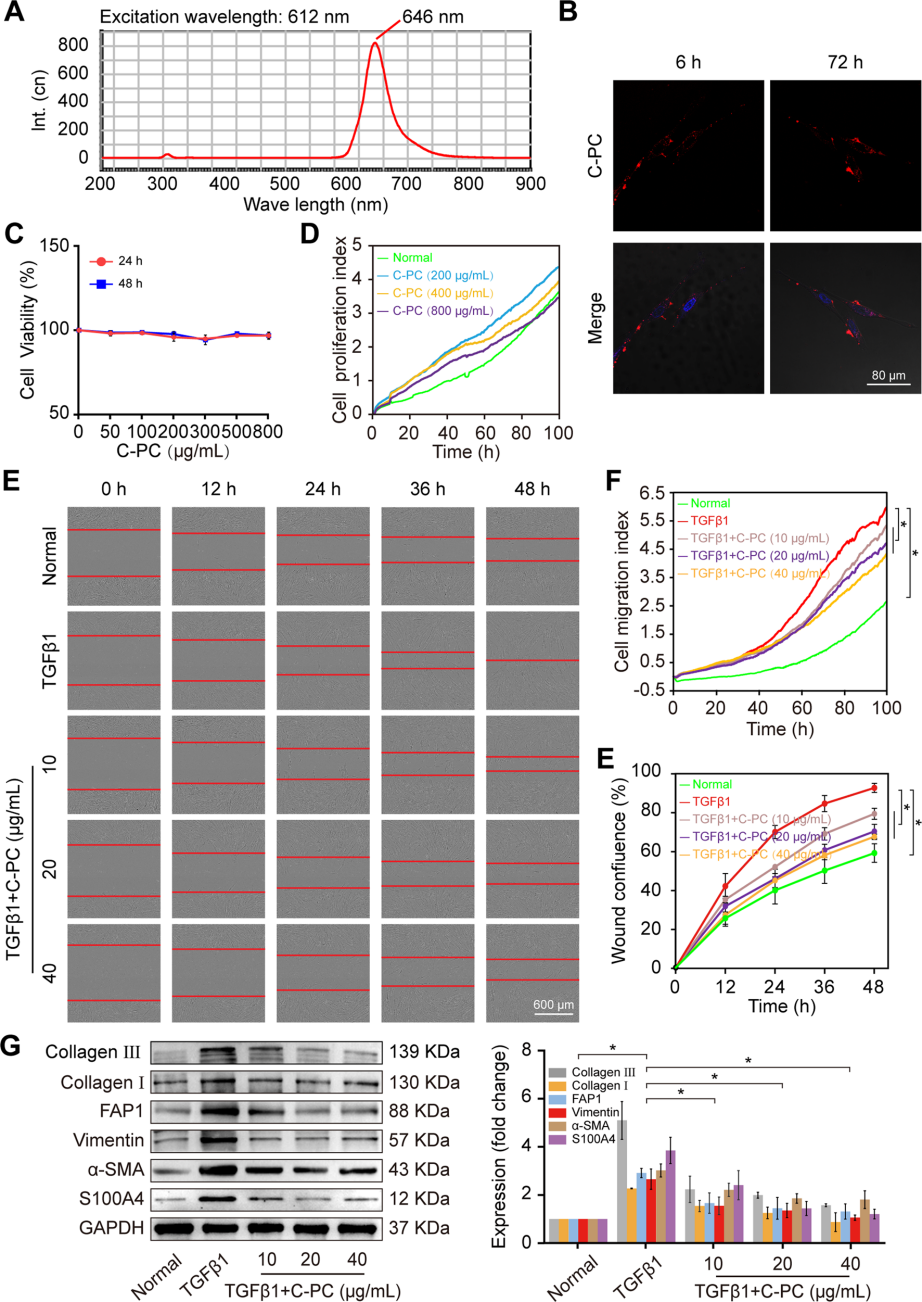
C-PC blocked pulmonary fibrogenesis in TGFβ1-induced MRC-5 cells. **A** Ultraviolet spectrophotometer detection showed that the excitation and emission wavelengths of C-PC were 612 and 646 nm, respectively. **B** Laser scanning confocal microscope observation showed that C-PC entered the cell after 6 h of treatment and mainly existed in the cytoplasm. **C** CCK-8 results displayed that C-PC was almost nontoxic to the viability of MRC-5 cells. **D** The real-time cellular analysis curve depicted that 200, 400, and 800 μg/mL C-PC had minimal effects on the activity of MRC-5 cells. **E** Wound healing results showed that 10, 20, and 40 μg/mL of C-PC could effectively inhibit the cell migration ability after TGFβ1 treatment. **F** The real-time cellular analysis curve depicted that compared with that of the TGFβ1 control group, 10, 20, and 40 μg/mL C-PC significantly inhibited the migration of the TGFβ1-treated cells. **G** Western blot analysis showed that C-PC treatment for 48 h significantly inhibited the expression of FAP1, S100A4, vimentin, α-SMA, and collagen I and III compared with those in the TGFβ1 group. The data were expressed as the mean ± SD from at least triplicate experiments

First, the spontaneous fluorescence characteristic of C-PC was detected using an ultraviolet spectrophotometer to identify whether C-PC can enter the cell. The results showed that the excitation and emission wavelengths of C-PC were 612 and 646 nm, respectively (Fig. 1A). These values were then applied to identify the intracellular localization of C-PC in MRC-5 cells by using a laser scanning confocal microscope. The images uncovered that C-PC entered the cell and mainly existed in the cytoplasm after C-PC administration to MRC-5 cells for 6 h, and until 72 h later, C-PC still mainly existed in the cytoplasm. (Fig. 1B). CCK-8 was used to detect the toxicity of C-PC for normal MRC-5 cells. The results showed that C-PC had no evident toxicity on normal MRC-5 cells cultured with different concentrations of C-PC (Fig. 1C). Similarly, real-time cellular analysis confirmed that different concentrations of C-PC were almost nontoxic to normal MRC-5 cells (Fig. 1D). The cells were treated with 5 ng/mL TGFβ1 and different concentrations of C-PC. The experimental results of wound healing using IncuCyte S3 showed that the C-PC treatment significantly reduced the migration rate of TGFβ1-treated MRC-5 cells compared with that of the TGFβ1 group (Fig. 1E). The Real-time cellular analysis curve also demonstrated this result (Fig. 1F). Western blot analysis showed that after C-PC treatment, the fibrotic protein markers including vimentin, α-SMA, and collagen I and III and the differentiation marker proteins including fibroblast activation protein 1 (FAP1) and S100 calcium-binding protein A4 (S100A4) were significantly inhibited (Fig. 1G). The above data indicated that C-PC inhibited fibroblast activation to block pulmonary fibrogenesis in vitro.

### C-PC ameliorated bleomycin (BLM)-induced pulmonary fibrosis in mice

**Fig. 2:**
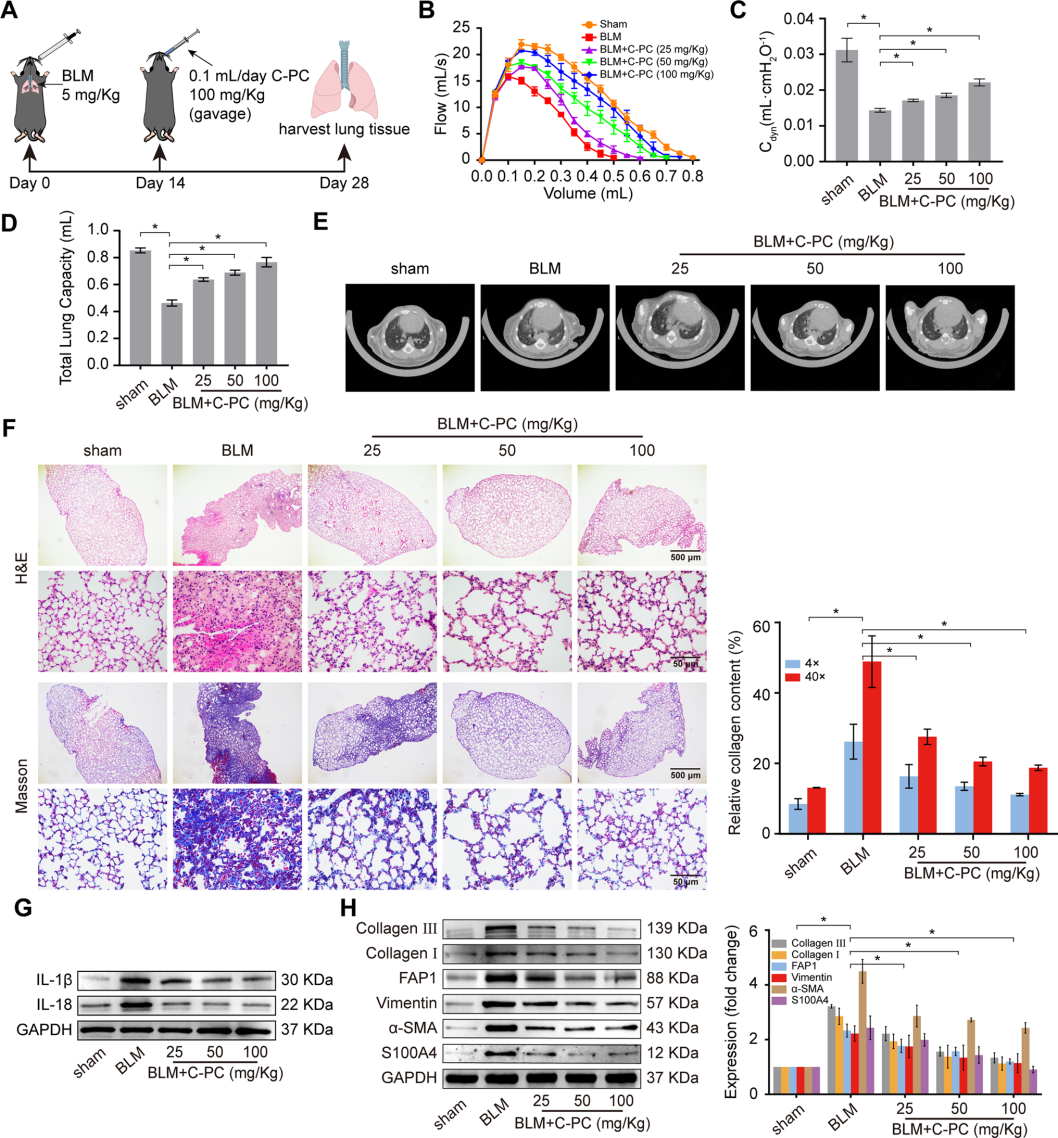
C-PC treatment ameliorated BLM-induced pulmonary fibrosis in mice. **A** Schematic of BLM tracheal spray and C-PC intragastric administration. **B** FVC testing showed that compared with the BLM group, the C-PC treatment group had significantly improved lung function. **C** The results of Cdyn in mice showed that the lung compliance of the BLM-treated group decreased, while the lung compliance of the C-PC-treated group increased. **D** Compared with the BLM treatment group, the total lung capacity of mice increased significantly in the C-PC treatment group. **E** MicroCT images depicted that the C-PC treatment group had no evident pulmonary fibrosis symptoms, and the BLM-treated mice had severe pulmonary fibrosis and lungs showing the typical honeycomb manifestation and ground-glass shadows. **F** H&E and Masson staining were performed to assess the pulmonary fibrosis degree. Alveolar walls thinned out, alveolar structure was clear, and collagen deposition decreased in the C-PC treatment group compared with those in the BLM treatment group. **G** Western blot showed that the expression of inflammation-related proteins decreased in the C-PC treatment group compared with the BLM group. **H** Western blot detection showed that the expression levels of FAP1, S100A4, vimentin, α-SMA, and collagen I and III were reduced by C-PC treatment compared with BLM treatment. The data were expressed as the mean ± SD from at least triplicate experiments

Next, we studied the antifibrotic effect of C-PC on BLM-induced pulmonary fibrosis in mice (Fig. 2A). First, the animal pulmonary function test system was used to detect the forced vital capacity (FVC) of mice. Compared with that of the mice treated with BLM alone, the lung function of the mice who received C-PC was significantly improved (Fig. 2B). The results of dynamic lung compliance (Cdyn) of mice showed that the lung compliance of the BLM-treated group was reduced compared to the sham-operated group, while the lung compliance of the C-PC-treated group increased (Fig. 2C). The results of the total lung volume (TLC) of mice showed that BLM treatment led to a decrease in the lung volume of mice. After C-PC treatment, the lung volume of mice was improved (Fig. 2D). The lungs of the mice were observed using a MicroCT imaging system for small animals. The images depicted that severe pulmonary fibrosis occurred in the BLM-treated mice, and their lungs had the typical honeycomb manifestation and ground-glass shadows. Meanwhile, the lung fibrosis symptoms were reduced in the C-PC-treated mice (Fig. 2E). H&E and Masson staining elucidated that the BLM-treated mice demonstrated typical pulmonary fibrosis characteristics such as alveolar wall thickening, excessive collagen accumulation, and slight inflammatory cell infiltration. By contrast, the manifestations of pulmonary fibrosis were reduced in the C-PC-treated mice, and the alveolar structure presented continuous and clear alveolar interstitial walls with no evident inflammatory cell infiltrations (Fig. 2F). Western blot results showed that the expression of IL-1β and IL-18 increased significantly after BLM treatment, but the inflammation decreased after C-PC treatment (Fig. 2G). Western blot results showed that the high expression levels of FAP1, S100A4, vimentin, α-SMA, and collagen I and III induced by BLM were substantially repressed by C-PC treatment (Fig. 2H). These findings were consistent with the in vitro studies, indicating that C-PC treatment significantly blocked pulmonary fibrosis in vivo.

### C-PC attenuated pulmonary fibrosis through down-regulating lncIAPF in vivo and in vitro

**Fig. 3:**
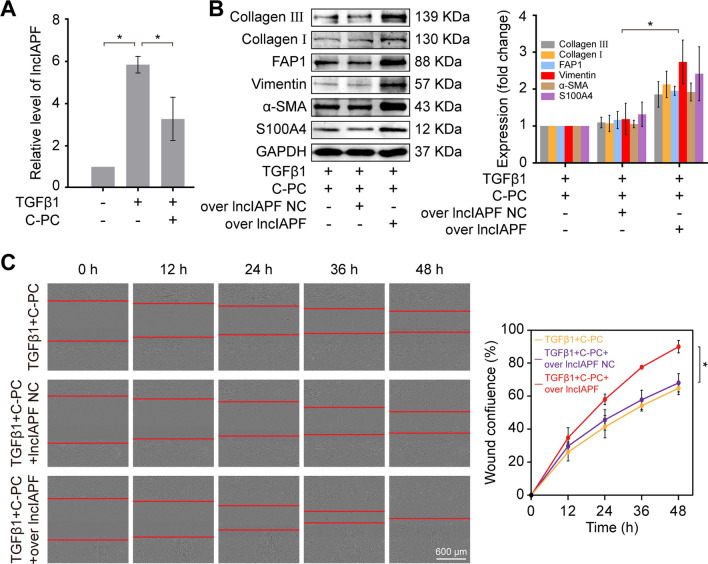
LncIAPF was verified as a target of C-PC in MRC-5 cells. **A** qRT-PCR results manifested that C-PC repressed lncIAPF expression. **B** Western blot analysis showed that lncIAPF overexpression reversed the inhibition of C-PC on fibrosis marker proteins and fibroblast activation marker proteins. **C** The wound healing experiment result showed that lncIAPF overexpression reversed the inhibitory effect of C-PC on cell migration. The data were expressed as the mean ± SD for at least triplicate experiments

LncIAPF can accelerate pulmonary fibrosis by activating the differentiation of fibroblast into myofibroblast (Zhang et al. 2022). Whether lncIAPF can be the therapeutic target of C-PC was further explored. qRT-PCR analysis showed that C-PC treatment significantly decreased the expression of lncIAPF (Fig. 3A), indicating that C-PC can act on lncIAPF. Rescue experiments were designed to prove that lncIAPF is the target of C-PC. Plasmids containing lncIAPF (overexpressed lncIAPF) and without lncIAPF (negative control of lncIAPF, NC) were designed, synthesized, and transfected into MRC-5 cells. Western blot result showed that lncIAPF overexpression significantly increased the expression levels of FAP1, S100A4, vimentin, α-SMA, and collagen I and III, which reversed the therapeutic effect of C-PC (Fig. 3B). Cellular wound healing assay revealed that lncIAPF overexpression accelerated cell migration and reversed the inhibitory effect of C-PC, which was consistent with the western blot result (Fig. 3C). These data indicated that lncIAPF was the target of C-PC, with the latter attenuating pulmonary fibrosis by down-regulating the former in vitro.

**Fig. 4:**
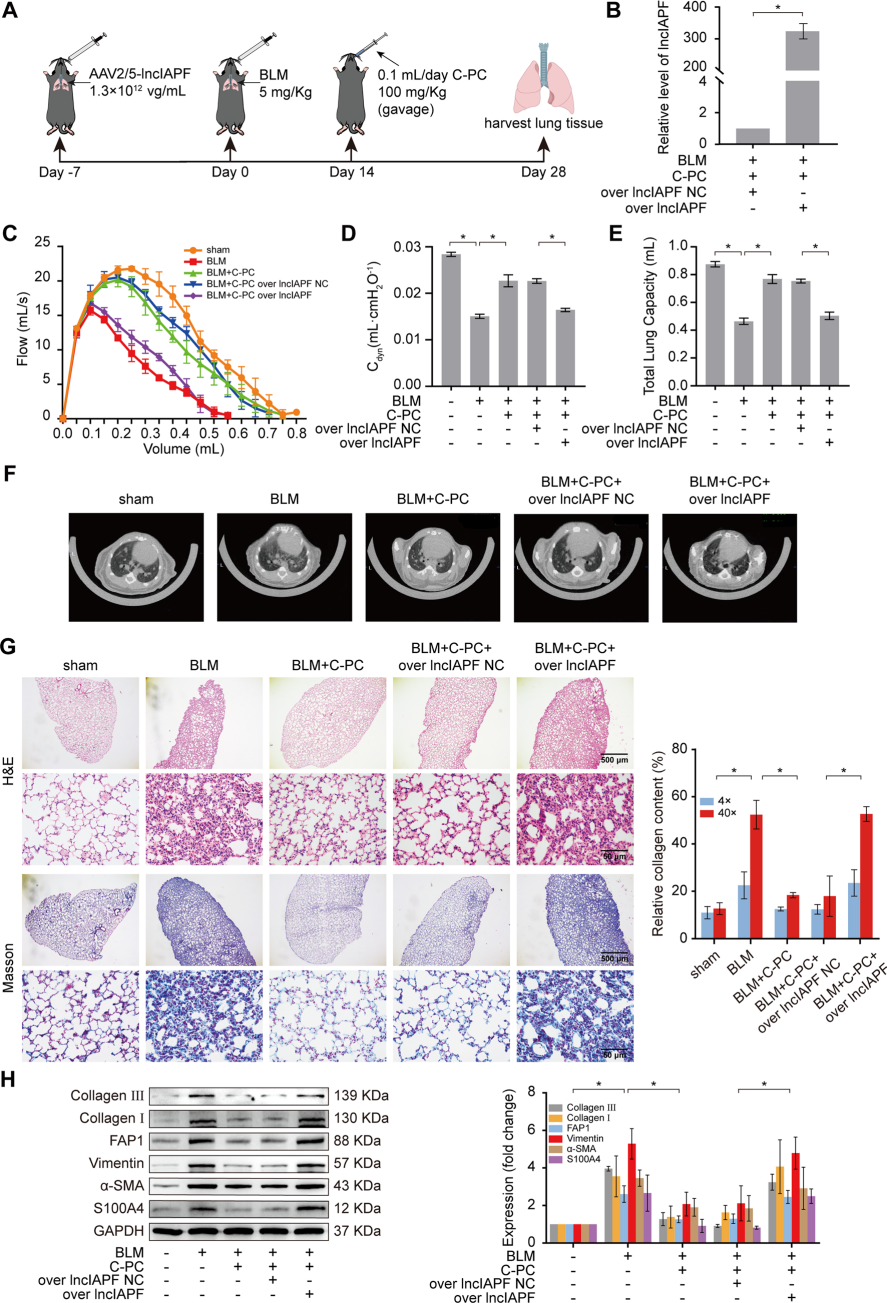
LncIAPF was verified as a target for C-PC action in mice. **A** Schematic of channel, dosage, and time for drug administration. **B** qRT-PCR results showed that the expression of lncIAPF in the lung tissues of mice infected with the adenovirus of overexpressing lncIAPF increased significantly. **C** LncIAPF overexpression aggravated the mouse lung function and reversed the therapeutic effect of C-PC. **D** The results of the rescue experiment showed that overexpression of lncIAPF reduced the lung compliance of mice, reversing the therapeutic effect of C-PC. **E** The rescue experiment showed that lncIAPF overexpression reduced TLC and reversed the therapeutic effect of C-PC. **F** MicroCT images showed that C-PC significantly reduced the symptoms of fibrosis caused by BLM, and lncIAPF overexpression reversed the therapeutic effect of C-PC. **G** H&E and Masson staining showed that lncIAPF overexpression aggravated the degree of pulmonary fibrosis, increased collagen deposition, and disordered alveolar structure compared with those of the C-PC treatment group. **H** Western blot analysis showed that lncIAPF overexpression increased the expression levels of FAP1, S100A4, vimentin, α-SMA, and collagen I and III, reversing the therapeutic effect of C-PC. The data were expressed as the mean ± SD for at least triplicate experiments

Rescue experiments in mice were performed to further confirm that the signal pathway of C-PC treatment is through lncIAPF. Adenovirus vector was used to coat the overexpressed lncIAPF and then sprayed into the lungs of mice through a tracheal spray needle so that the virus infected the mice, thus constructing a mouse model of overexpressed lncIAPF (Fig. 4A). qRT-PCR results showed that compared with that of the control group, the lncIAPF expression in the lung tissues of the mice infected with adenovirus of overexpressing lncIAPF increased significantly, indicating that the animal model used for rescue experiment was successfully established (Fig. 4B). An animal pulmonary function test system was then used to detect the FVC of mice and uncovered that lncIAPF overexpression weakened the lung function and reversed the therapeutic effect of C-PC (Fig. 4C). The results of Cdyn of mice showed that compared to the sham-operated group, the lung compliance of the BLM-treated group decreased. After C-PC treatment, the lung compliance of mice increased. However, in mice with overexpressed lncIAPF, lung compliance decreased, reversing the therapeutic effect of C-PC (Fig. 4D). The TLC result of mice showed that BLM treatment reduced the total lung capacity. After C-PC treatment, the TLC was significantly improved. Overexpressed lncIAPF reduced the TLC of mice, reversing the therapeutic effect of C-PC (Fig. 4E). The lung condition of mice with overexpressed lncIAPF was observed using the MicroCT system. The images showed that compared with the C-PC treatment group, the mice with lncIAPF overexpression had typical honeycomb-like manifestations, ground-glass shadows, and serious lung fibrosis, indicating that the therapeutic effect of C-PC was reversed (Fig. 4F). H&E and Masson staining also depicted that the alveolar structure of overexpressed lncIAPF mice was disordered, the extracellular collagen deposition increased significantly, and the improved effect of C-PC was reversed (Fig. 4G). Western blot analysis detected that the protein levels of FAP1, S100A4, vimentin, α-SMA, and collagen I and III were significantly increased by overexpressed lncIAPF, contrary to the effect of C-PC (Fig. 4H). All the above findings indicated that the signal pathway of C-PC treatment is through lncIAPF, and lncIAPF can be a target of C-PC in vivo and in vitro.

### C-PC inhibited lncIAPF expression by blocking the nuclear translocation of ATF3

**Fig. 5:**
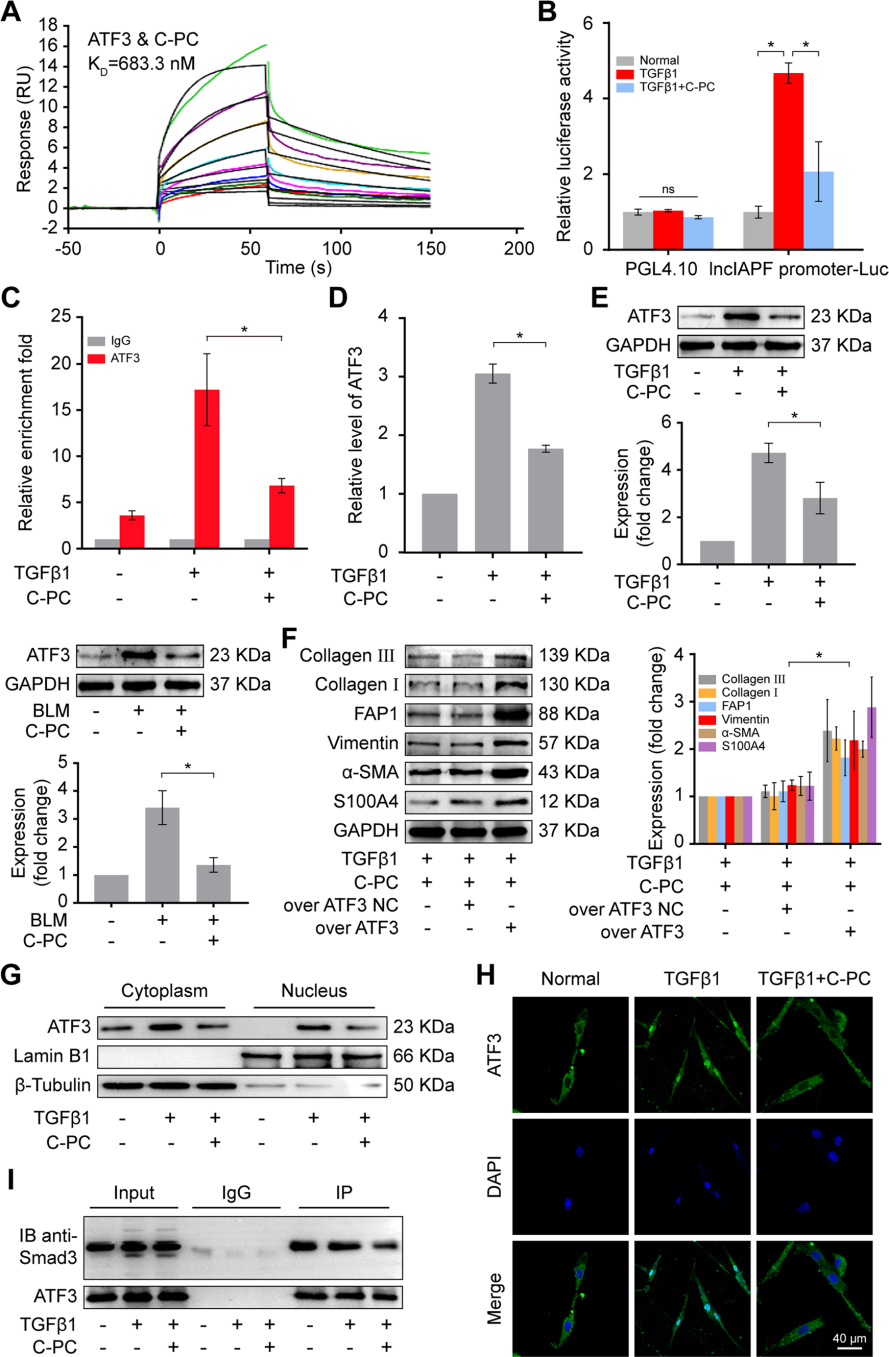
C-PC inhibited lncIAPF transcription by blocking the nuclear translocation of ATF3. **A** SPR results showed that C-PC had good binding affinity with ATF3. **B** Binding activity of ATF3 to lncIAPF detected by double luciferase report method. The results showed that TGFβ1 promoted the binding of ATF3 to lncIAPF, and C-PC inhibited this process. **C** CUT & RUN-PCR results showed that the binding amount between ATF3 and lncIAPF was enhanced by TGFβ1 but decreased by C-PC. **D** qRT-PCR results showed that C-PC reduced ATF3 mRNA expression. **E** Western blot results showed that ATF3 protein expression decreased in vivo and in vitro. **F** A rescue experiment of Western blot showed that the expression levels of FAP1, S100A4, vimentin, α-SMA, and collagen I and III decreased under C-PC treatment and increased under ATF3 overexpression. The overexpressed ATF3 reversed the inhibitory effect of C-PC treatment on these proteins. **G** Western blot results showed that the ATF3 expression in the cytoplasm and nucleus increased after TGFβ1-treated, but C-PC decreased the ATF3 expression and blocked the nuclear translocation of ATF3. β-Tubulin was used as cytoplasm control and Lamin B1 as nuclear control. **H** Immunofluorescence images of a laser confocal microscope depicted that ATF3 was transferred to the nucleus under TGFβ1 action, and C-PC prevented ATF3 from entering the nucleus. The blue fluorescence indicates the nucleus is stained by DAPI. The green fluorescence indicates ATF3. **I** Co-IP experiment results showed that ATF3 had a binding relationship with Smad3. Compared with that in the TGFβ1 group, the binding between Smad3 and ATF3 in the C-PC treatment group was significantly reduced. The data were expressed as the mean ± SD for at least triplicate experiments

Given that ATF3 can enhance lncIAPF transcription, whether C-PC inhibits lncIAPF expression through ATF3 was further studied. Surface plasmon resonance (SPR) results showed that the equilibrium dissociation constant of C-PC and ATF3 was 683.3 nM, indicating they had a good binding affinity (Fig. 5A). To verify whether ATF3 can bind to the promoter region of lncIAPF, and thus activate the transcription of lncIAPF, we constructed the promoter region of lncIAPF into pGL4.10-CXCL10 vector, and carried out a double luciferase activity detection experiment. These data show that ATF3 can bind to the promoter region of lncIAPF and participate in the transcription of lncIAPF, and TGFβ1 promotes the enrichment of ATF3 in the promoter of lncIAPF, while C-PC inhibits this process (Fig. 5B). The primers for the promoter region of lncIAPF were designed and CUT & RUN-PCR experiment. The results unveiled that lncIAPF bound with ATF3; the binding amount of lncIAPF and ATF3 was significantly increased by TGFβ1 but reduced by C-PC (Fig. 5C). qRT-PCR and western blot results confirmed that C-PC significantly reduced the expression of ATF3 at mRNA and protein levels (Fig. 5D, E). A plasmid containing ATF3 (overexpressed ATF3) and a plasmid without ATF3 (ATF3 negative control) were designed, synthesized, and transfected into MRC-5 cells. The rescue experiment revealed that C-PC significantly decreased the expression levels of FAP1, S100A4, vimentin, α-SMA, and collagen I and III, but ATF3 overexpression reversed this inhibitory effect (Fig. 5F), indicating that the therapeutic effect of C-PC depended on ATF3. Given that ATF3 transcriptional activity on lncIAPF is exerted by translocation from cytoplasm to nucleus, a nuclear–cytoplasmic separation experiment was conducted to determine the effect of C-PC on ATF3 nuclear translocation. Western blot results showed that compared with that of the control group, ATF3 expression was significantly increased in the cytoplasm and nucleus of the TGFβ1 treatment group but was significantly decreased in the nucleus of the C-PC treatment group (Fig. 5G). A confocal microscope was used to observe whether the subcellular localization of ATF3 changed under C-PC action. ATF3 expression increased significantly in the nucleus of the TGFβ1 treatment group but decreased significantly in the nucleus of the C-PC treatment group (Fig. 5H). Given that TGFβ1 promotes lung fibrogenesis through Smad3, the Co-IP experiment with ATF3 antibody was further carried out. The results showed that ATF3 is bound with the transcription factor Smad3. Compared with that in the TGFβ1 group, the binding between Smad3 and ATF3 in the C-PC treatment group was reduced (Fig. 5I). The data indicated that TGFβ1 stimulated ATF3 to be recruited into the protein complex containing Smad3 and promoted lncIAPF transcription, but C-PC prevented the binding between ATF3 and Smad3 by blocking ATF3 translocation. All the above data suggested that C-PC inhibits lncIAPF transcription by blocking ATF3 translocation from the cytoplasm to the nucleus, resulting in a decrease in lncIAPF biogenesis.

### C-PC inhibited lncIAPF profibrogenic function by down-regulating its binding protein HuR

**Fig. 6:**
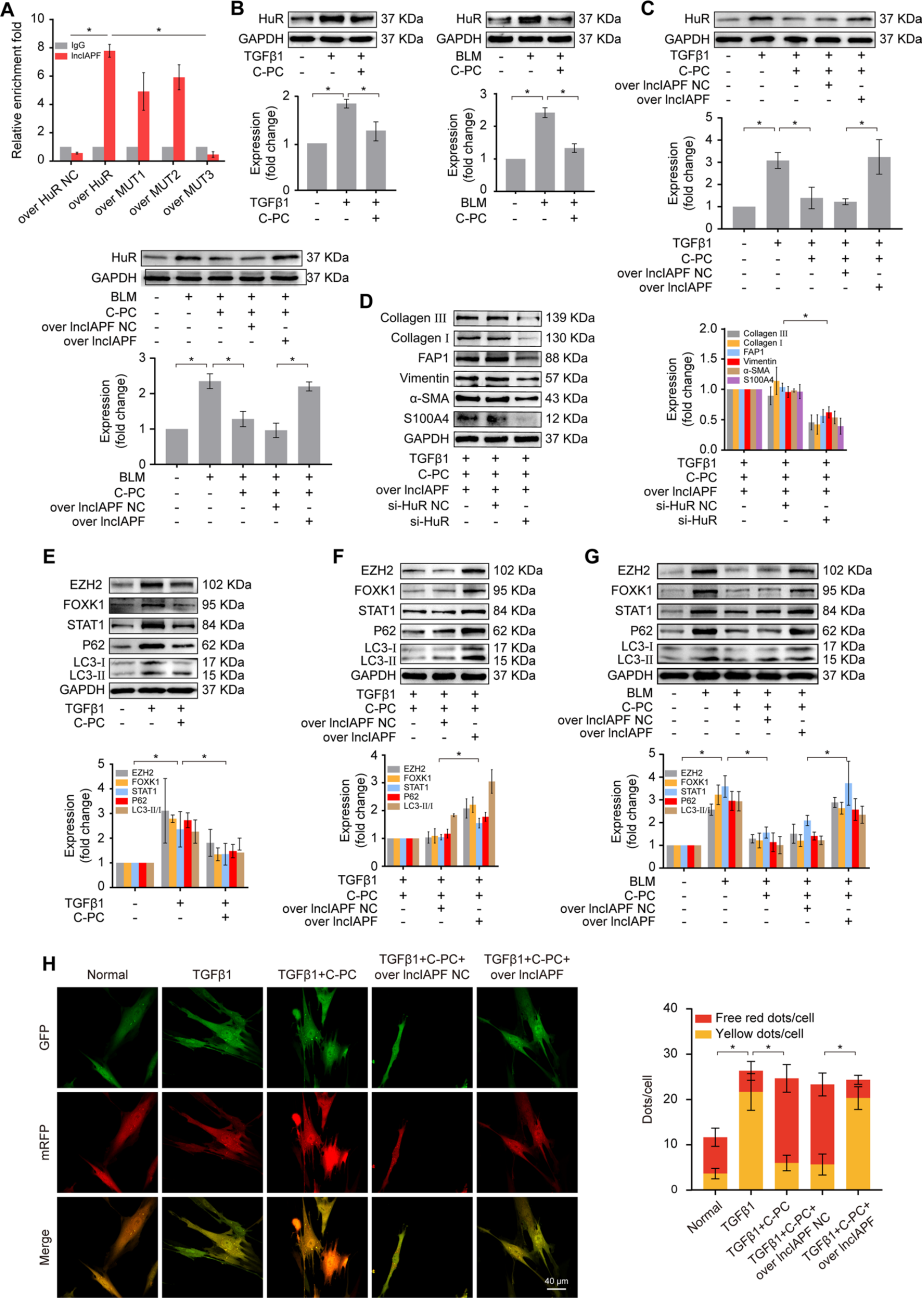
C-PC promoted autophagy to attenuate pulmonary fibrosis via a down-regulating lncIAPF-HuR-mediated signal pathway. **A** RIP results showed that MUT3 residue was the domain of interaction between lncIAPF and HuR. **B** Western blot results showed that C-PC down-regulated HuR expression in vitro and in vivo. **C** A rescue experiment of western blot showed that C-PC reduced HuR expression, and lncIAPF overexpression reversed the downward trend caused by C-PC. **D** Western blot analysis showed that after silencing HuR, the expression levels of FAP1, S100A4, vimentin, α-SMA, and collagen I and III were down-regulated, which reversed the up-regulation caused by lncIAPF overexpression. **E** Western blot results showed that C-PC decreased the expression levels of EZH2, STAT1, FOXK1, P62, and LC3I/II in MRC-5 cells treated with TGFβ1. **F** Rescue experiment results showed that lncIAPF overexpression reversed the enhancement of autophagy by C-PC. **G** In mice treated with overexpressed lncIAPF, the expression of autophagy-related proteins increased. **H** Images of laser confocal microscopy showed that in the TGFβ1 treatment group, the red fluorescence representing normal autophagy is reduced, and the yellow spots representing autophagy abnormalities increase. while in the C-PC treatment group, the yellow fluorescence decreased and the red spots increased, indicating that C-PC promoted autophagy. LncIAPF overexpression reversed the enhancement of C-PC on autophagy. The data were expressed as the mean ± SD for at least a triplicate experiment

A lncRNA exerts its regulatory function through its binding protein. HuR is the binding protein of lncIAPF, but its binding domain is still unknown. Therefore, a HuR truncation experiment was performed to identify the binding domain between lncIAPF and HuR. Four HuR mutant plasmids with Flag tags were constructed according to the amino acid (aa) sequence of HuR, namely, WT HuR, knocked out from 20–98 aa, (MUT1), 106–186 aa (MUT2), and 244–322 aa (MUT3). RIP experiment was performed and revealed that MUT3 reduced the combination of lncIAPF and HuR, suggesting that MUT3 residue is the binding domain between lncIAPF and HuR (Fig. 6A). Whether C-PC inhibits lncIAPF regulatory function through HuR was explored. Western blot results showed that C-PC decreased the HuR protein expression in vivo and in vitro (Fig. 6B). Rescue experiment confirmed that the inhibition of C-PC on HuR was reversed by lncIAPF overexpression (Fig. 6C), indicating that C-PC affected HuR expression through lncIAPF. According to the full-length sequence of HuR, HuR's intelligent silencing RNA (si-HuR) was designed, synthesized, and transfected into MRC-5 cells. lncIAPF overexpression reversed the downward trend of vimentin, α-SMA, type I, type III collagen, FAP1, and S100A4 caused by C-PC treatment. However, si-HuR induced a significant decrease in these proteins and reversed the promoting effect of lncIAPF overexpression on these proteins (Fig. 6D). Western blot showed that the expression levels of EZH2, STAT1, FOXK1, P62 and LC3I/II decreased in C-PC group compared with TGFβ1 group (Fig. 6E). The rescue experiment result of Western blot showed that compared with those in the C-PC treatment group, the expression levels of autophagy marker proteins such as EZH2, STAT1 and FOXK1, P62, and LC3I and II increased in the overexpression lncIAPF group, indicating that the enhancement of autophagy flux by C-PC treatment depended on lncIAPF (Fig. 6F). The rescue experiment showed that the overexpressed lncIAPF reversed the downward trend of autophagy-related proteins caused by C-PC, indicating that the enhancement of autophagy flux by C-PC treatment depended on lncIAPF (Fig. 6G). Furthermore, the double fluorescent mRFP-GFP-LC3 adenovirus was used to detect the effect of C-PC on autophagy. Red fluorescence represents autophagy-lysosome, which means autophagy is normal, and yellow fluorescence means autophagy is partially blocked. Images of laser confocal microscopy showed that the red fluorescence of the TGFβ1 group was weakened and the yellow spots were increased. Compared with the TGFβ1 group, the red spots in the C-PC treatment group were increased, which indicated that C-PC promoted autophagy. The yellow spots in the lncIAPF overexpression group increased obviously, indicating that the over-expressed lncIAPF reversed the autophagy enhancement induced by C-PC (Fig. 6H). All the above data suggested that C-PC promotes autophagy to attenuate pulmonary fibrosis via down-regulating the lncIAPF-HuR-mediated signal pathway.

## Discussion

**Fig. 7:**
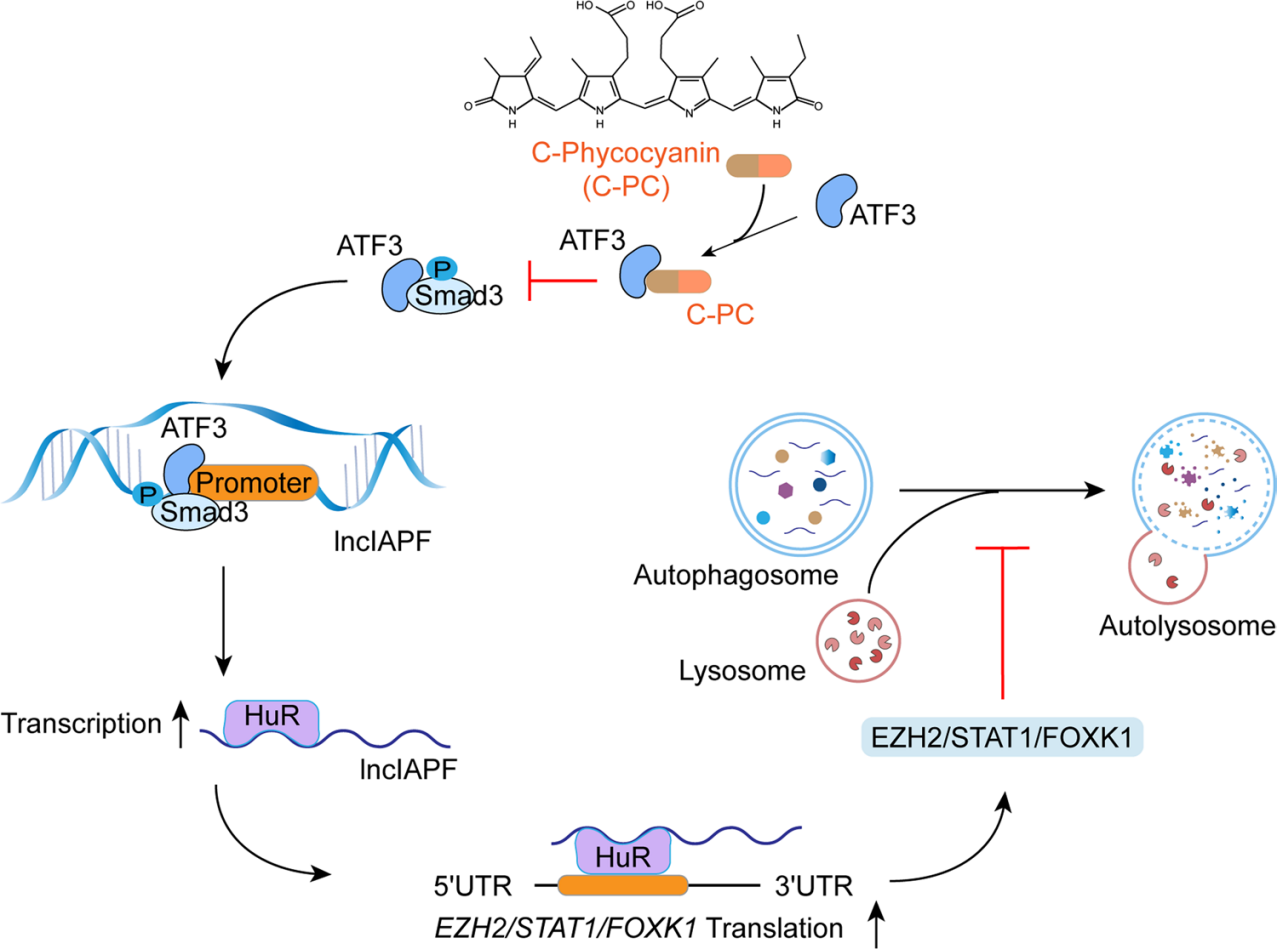
C-PC reinforced autophagy to block pulmonary fibrogenesis by inhibiting lncIAPF biogenesis

This study revealed that C-PC inhibited fibroblast–myofibroblast differentiation to block pulmonary fibrogenesis by inhibiting lncIAPF biogenesis. The experiments such as SPR, CUT & RUN-PCR, Co-IP, and nuclear–cytoplasm separation clarified that C-PC prevented the binding of ATF3 and Smad3 via blocking the nuclear translocation of ATF3, thereby hindering lncIAPF transcription. HuR truncation construction, RIP, and other experiments unveiled that lncIAPF exerted its profibrogenic function through its binding protein HuR, a negative regulator of autophagy, and the binding domain of HuR was in the 244–322 amino acid region. C-PC promoted autophagy via down-regulating lncIAPF-HuR, consequently attenuating pulmonary fibrosis. In summary, C-PC alleviates pulmonary fibrosis through the ATF3/Smad3-lncIAPF-HuR signal pathway that targets autophagy (Fig. 7).

Although the pathogenesis of pulmonary fibrosis has not been fully clarified, the activation of fibroblast–myofibroblast transition is considered the most common pathological feature. Therefore, targeting fibroblast activation to develop drugs is a strategy for treating pulmonary fibrosis (Zhang et al. 2019a, b). For example, the activation of phospho-src homology-2 domain-containing phosphotase2 (SHP2) can induce interferon-γ (IFN-γ) resistance, thus inhibiting the antifibrosis gene regulated by IFN-γ. SHP2 inhibitor PHPS1 or BIBF 1120 can block the activation of SHP2, thus overcoming the drug resistance of IFN-γ and promoting the antifibrosis effect. Hence, the combination therapy of PHPS1 + IFN-γ or BIBF 1120 + IFN-γ significantly inhibits fibroblast–myofibroblast transformation to mitigate pulmonary fibrosis (Chang et al. 2021). Platelet-rich plasma reversed the decreased expression of vascular endothelial growth factor (VEGF)-A and VEGF receptor (VEGFR)-1 induced by TGFβ1, thus inhibiting the TGFβ1/Smad3 signaling pathway mediated by VEGF-A/VEGFR-1 and preventing the transformation of fibroblasts to myofibroblasts (Chellini et al. 2018). This study discovered that C-PC inhibited fibroblast–myofibroblast differentiation to block pulmonary fibrogenesis through the ATF3/Smad3-lncIAPF-HuR signal pathway.

ATF3 is a stress-induced transcription factor that can establish appropriate cellular responses by regulating the expression of target genes (Ku and Cheng 2020). ATF3 is involved in the pathological process of many diseases and regulates metabolism, immunity, inflammation, and tumorigenesis (Liu et al. 2020; Wang et al. 2020a, b; Basak et al. 2023). For example, ATF3 promotes p65 deacetylation by recruiting histone deacetylase 1, thus blocking the NF-κB signaling pathway and the transcription of inflammatory response genes (Kwon et al. 2015). ATF3 not only plays a vital role in inducing NAFLD and type 2 diabetes mediated by oxidative stress but also can lead to liver fibrosis by activating hepatic stellate cells (Kim et al. 2017; Shi et al. 2020). However, the expression and function of ATF3 in pulmonary fibrosis have not been widely explored. Our findings illustrated that ATF3 can interact with transcription factor Smad3 to promote lncIAPF transcription. C-PC can inhibit the nuclear translocation of ATF3 to block its binding with Smad3, resulting in reduced lncIAPF transcription.

Autophagy is a normal cell homeostasis process, and autophagy deficiency is related to many diseases (Levine and Kroemer 2019). Decreased autophagy activity can cause the accumulation of damaged macromolecules and organelles to promote pulmonary fibrogenesis (López-Otín et al. 2013; Hill et al. 2019). As a negative autophagy regulator, HuR is involved in various types of fibrosis. For example, HuR promotes the production of BECN1/Beclin1 by binding to AREs of BECN1 mRNA, activates the degradation of autophagy ferritin, and finally leads to iron-dependent siderosis in liver fibrosis (Zhang et al. 2018). lncRNA-Safe plays a crucial role in cardiac fibrosis by promoting the stability of Sfrp2 mRNA and the ability of protein expression mediated by the Safe-Sfrp2-HuR complex (Hao et al. 2019). As a fibrotic factor, lncIAPF interacts with HuR to promote pulmonary fibrosis by blocking autophagy. This study showed that C-PC inhibited the fibrotic function of lncIAPF by weakening the stability of HuR, thus promoting autophagy and attenuating pulmonary fibrosis.

In summary, C-PC alleviates pulmonary fibrosis through the ATF3/Smad3-lncIAPF-HuR signal pathway that targets autophagy and can be a potential drug for treating pulmonary fibrosis. The exploration of C-PC with biological molecules will help us understand the mechanism of this drug on the molecular level and provide valuable information for designing new drugs.

### Supplementary Information

Below is the link to the electronic supplementary material.

Supplementary file1 (PDF 16066 kb)

Supplementary file2 (PDF 940 kb)

## Data Availability

The data that supports the findings of this study are available in the supplementary material of this article.
