# Supplementary material for: C-phycocyanin reinforces autophagy to block pulmonary fibrogenesis by inhibiting lncIAPF biogenesis
Source: Arch Pharm Res. 2024 Jul 22;47(7):659–74. doi: 10.1007/s12272-024-01508-y (PMC11300487; doi:10.1007/s12272-024-01508-y)
Supplement: Supplementary file 1 — Supplementary file1 (PDF 16066 kb) [file 12272_2024_1508_MOESM1_ESM.pdf]

Figure 1G-1:

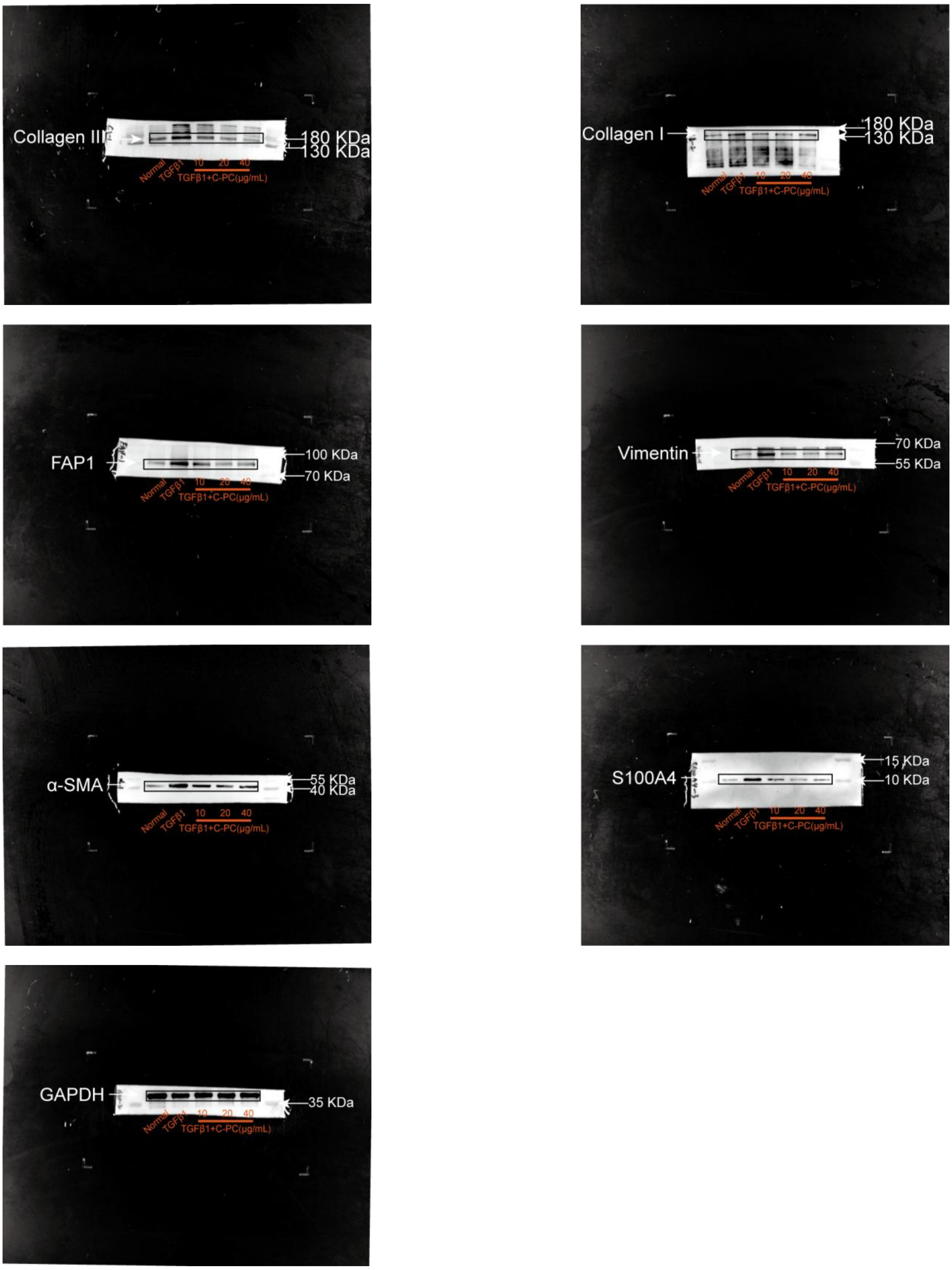

Collagen III
180 KDa
130 KDa
Normal
TGFβ1
10
20
40
TGFβ1+C-PC(μg/mL)
Collagen I
180 KDa
130 KDa
Normal
TGFβ1
10
20
40
TGFβ1+C-PC(μg/mL)
FAP1
100 KDa
70 KDa
Normal
TGFβ1
10
20
40
TGFβ1+C-PC(μg/mL)
Vimentin
70 KDa
55 KDa
Normal
TGFβ1
10
20
40
TGFβ1+C-PC(μg/mL)
α-SMA
55 KDa
40 KDa
Normal
TGFβ1
10
20
40
TGFβ1+C-PC(μg/mL)
S100A4
15 KDa
10 KDa
Normal
TGFβ1
10
20
40
TGFβ1+C-PC(μg/mL)
GAPDH
35 KDa
Normal
TGFβ1
10
20
40
TGFβ1+C-PC(μg/mL)

Figure 1G-2:

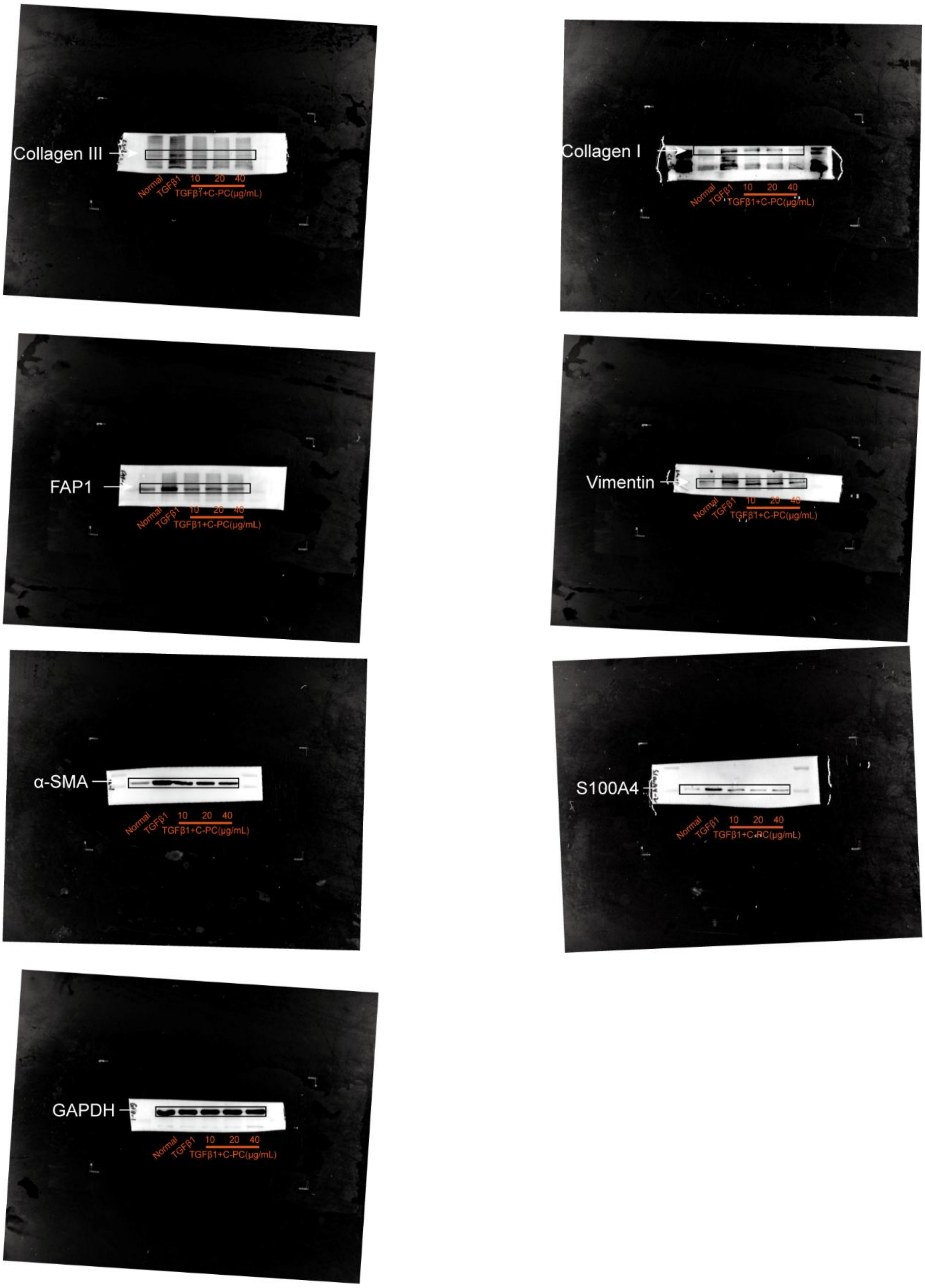
Collagen III
Normal
TGFβ1
10 20 40
TGFβ1+C-PC(μg/mL)
Collagen I
Normal
TGFβ1
10 20 40
TGFβ1+C-PC(μg/mL)
FAP1
Normal
TGFβ1
10 20 40
TGFβ1+C-PC(μg/mL)
Vimentin
Normal
TGFβ1
10 20 40
TGFβ1+C-PC(μg/mL)
α-SMA
Normal
TGFβ1
10 20 40
TGFβ1+C-PC(μg/mL)
S100A4
Normal
TGFβ1
10 20 40
TGFβ1+C-PC(μg/mL)
GAPDH
Normal
TGFβ1
10 20 40
TGFβ1+C-PC(μg/mL)

Figure 1G-3:

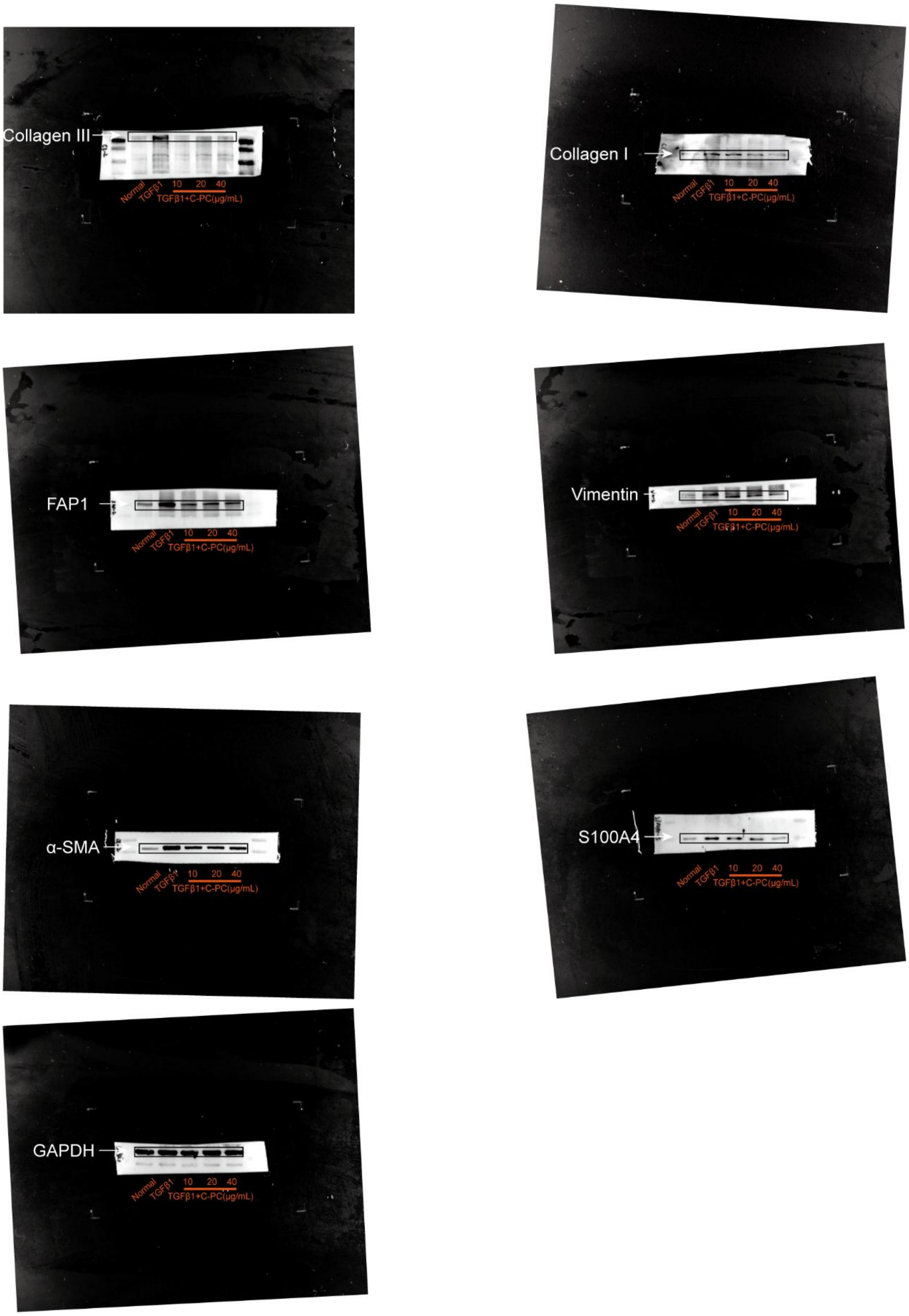
Collagen III
Normal
TGFβ1
10 20 40
TGFβ1+C-PC(μg/mL)
Collagen I
Normal
TGFβ1
10 20 40
TGFβ1+C-PC(μg/mL)
FAP1
Normal
TGFβ1
10 20 40
TGFβ1+C-PC(μg/mL)
Vimentin
Normal
TGFβ1
10 20 40
TGFβ1+C-PC(μg/mL)
α-SMA
Normal
TGFβ1
10 20 40
TGFβ1+C-PC(μg/mL)
S100A4
Normal
TGFβ1
10 20 40
TGFβ1+C-PC(μg/mL)
GAPDH
Normal
TGFβ1
10 20 40
TGFβ1+C-PC(μg/mL)

Figure 2G-1:

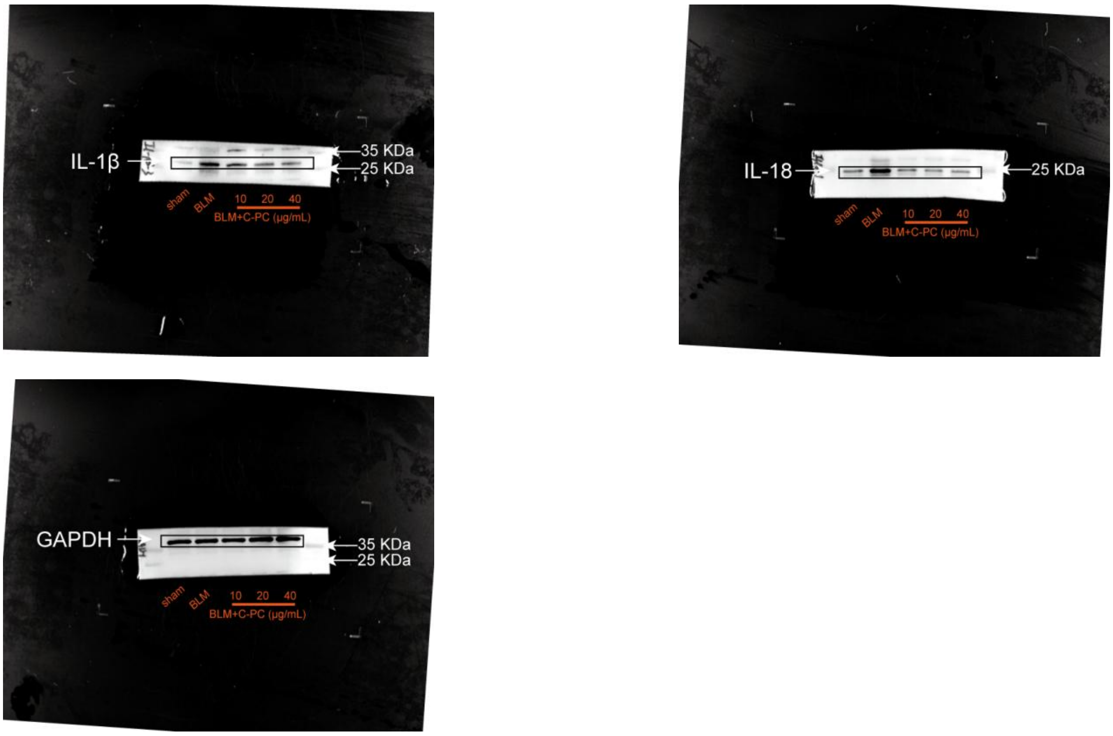

Figure 2G-2:

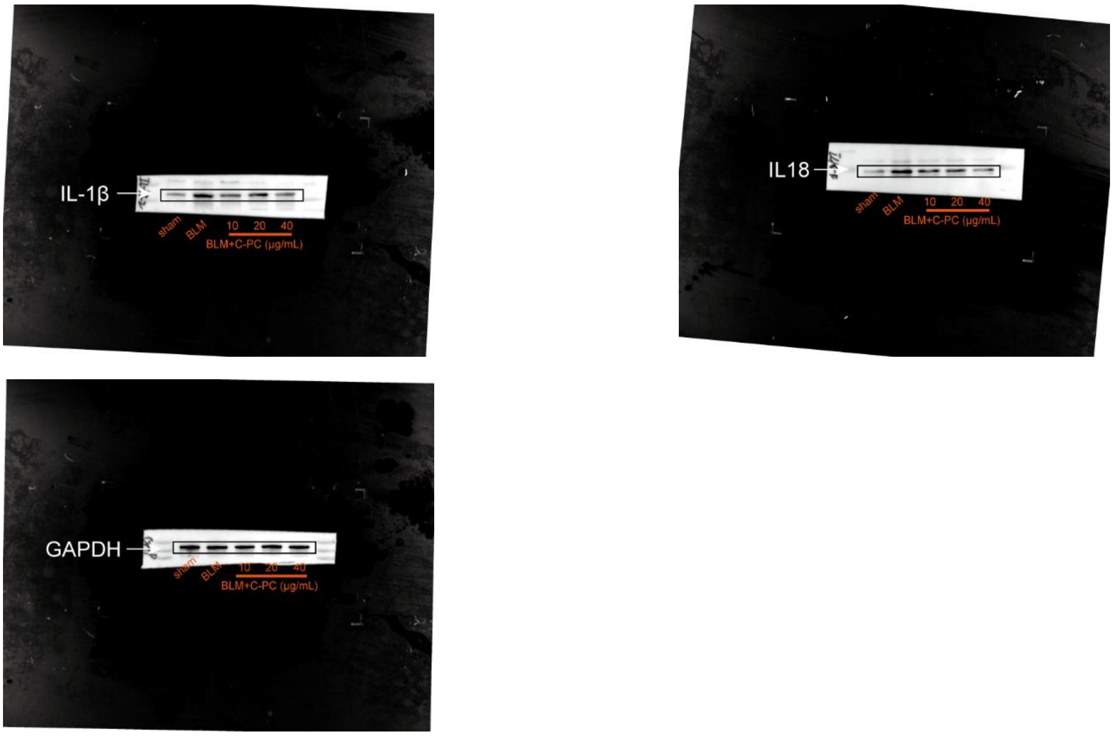

Figure 2H-1:

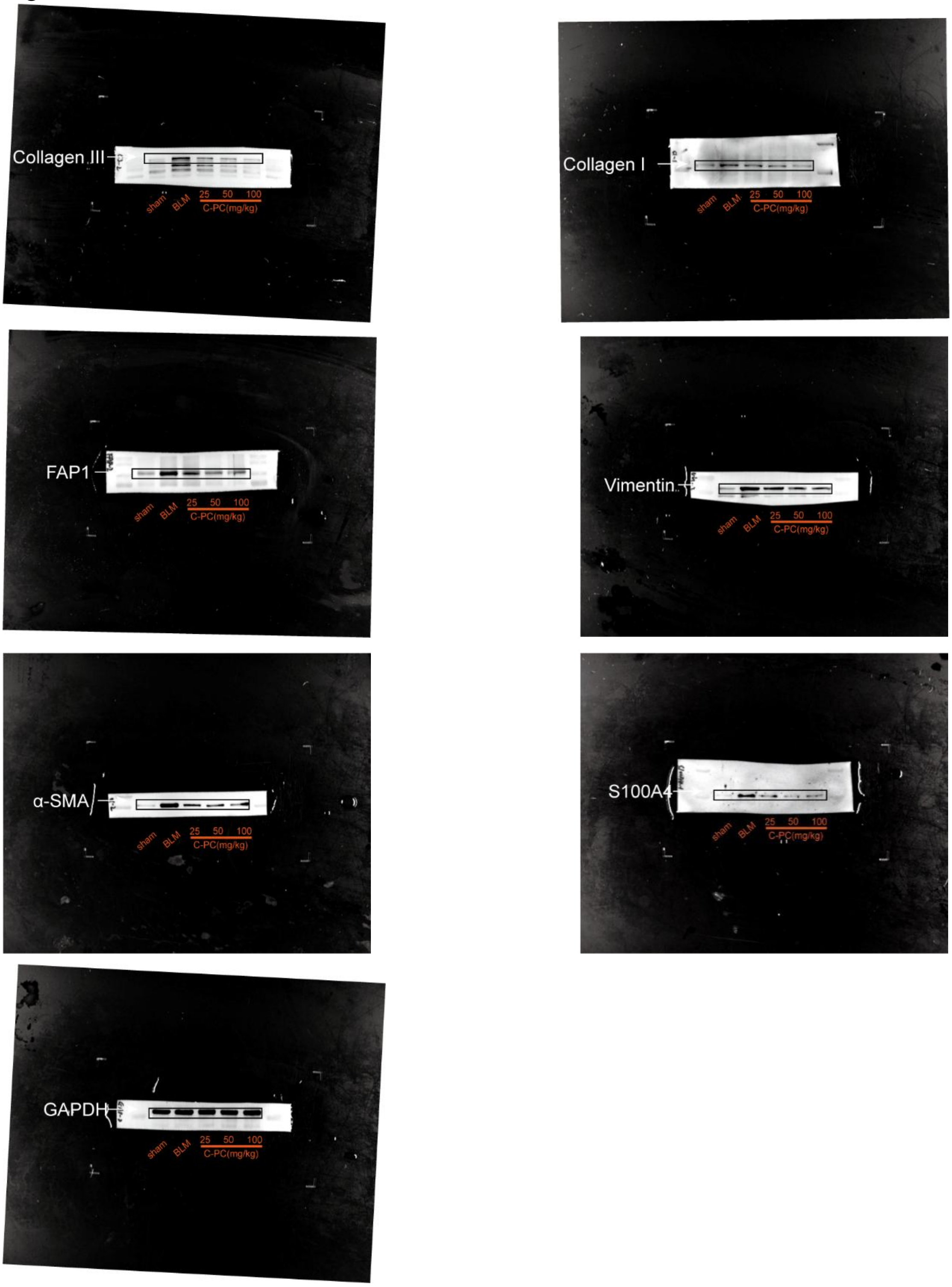
Collagen III
sham
BLM
25 50 100
C-PC(mg/kg)
Collagen I
sham
BLM
25 50 100
C-PC(mg/kg)
FAP1
sham
BLM
25 50 100
C-PC(mg/kg)
Vimentin
sham
BLM
25 50 100
C-PC(mg/kg)
α-SMA
sham
BLM
25 50 100
C-PC(mg/kg)
S100A4
sham
BLM
25 50 100
C-PC(mg/kg)
GAPDH
sham
BLM
25 50 100
C-PC(mg/kg)

Figure 2H-2:

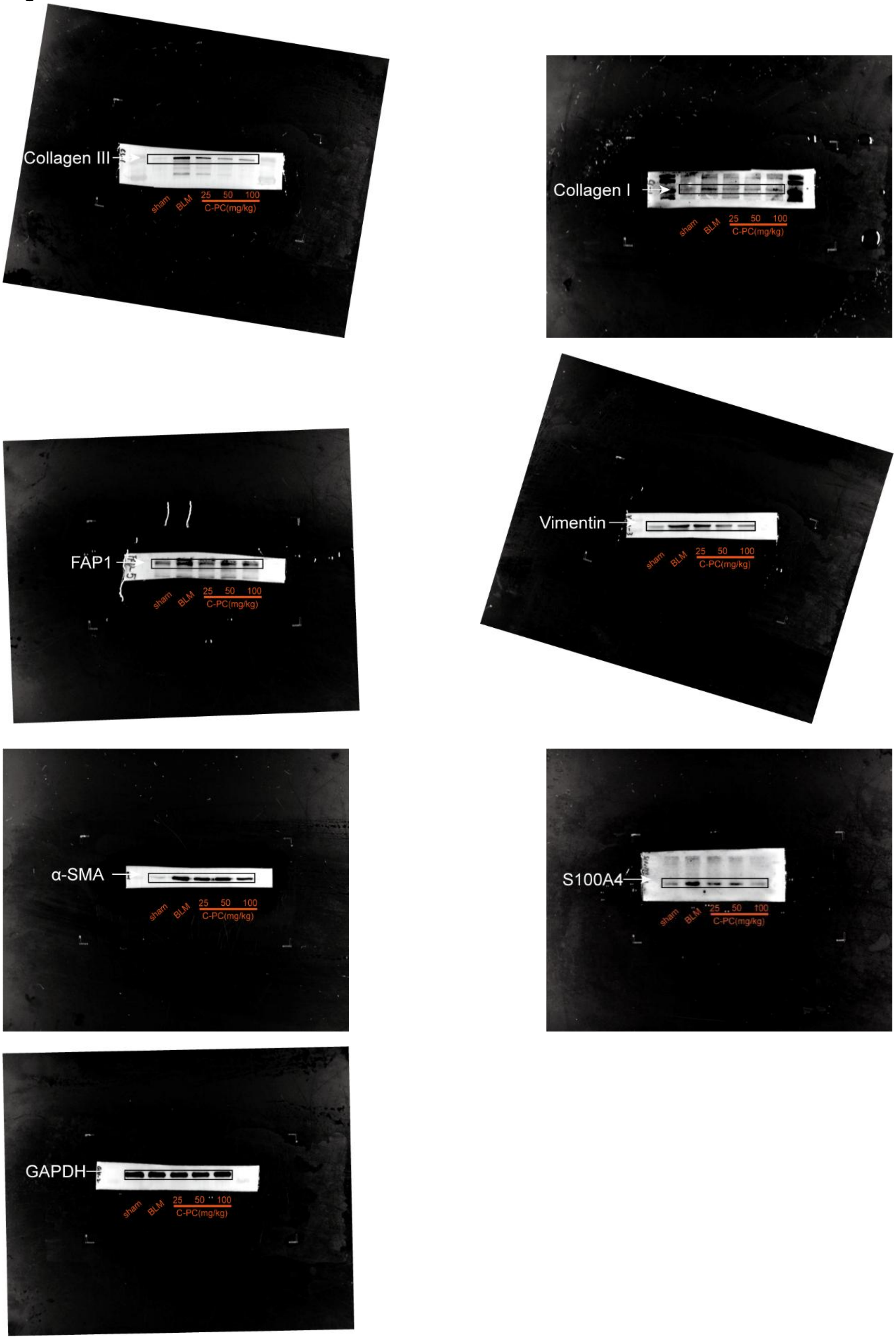
Collagen III
sham
BLM
25 50 100
C-PC(mg/kg)
Collagen I
sham
BLM
25 50 100
C-PC(mg/kg)
FAP1
sham
BLM
25 50 100
C-PC(mg/kg)
Vimentin
sham
BLM
25 50 100
C-PC(mg/kg)
α-SMA
sham
BLM
25 50 100
C-PC(mg/kg)
S100A4
sham
BLM
25 50 100
C-PC(mg/kg)
GAPDH
sham
BLM
25 50 100
C-PC(mg/kg)

Figure 2H-3:

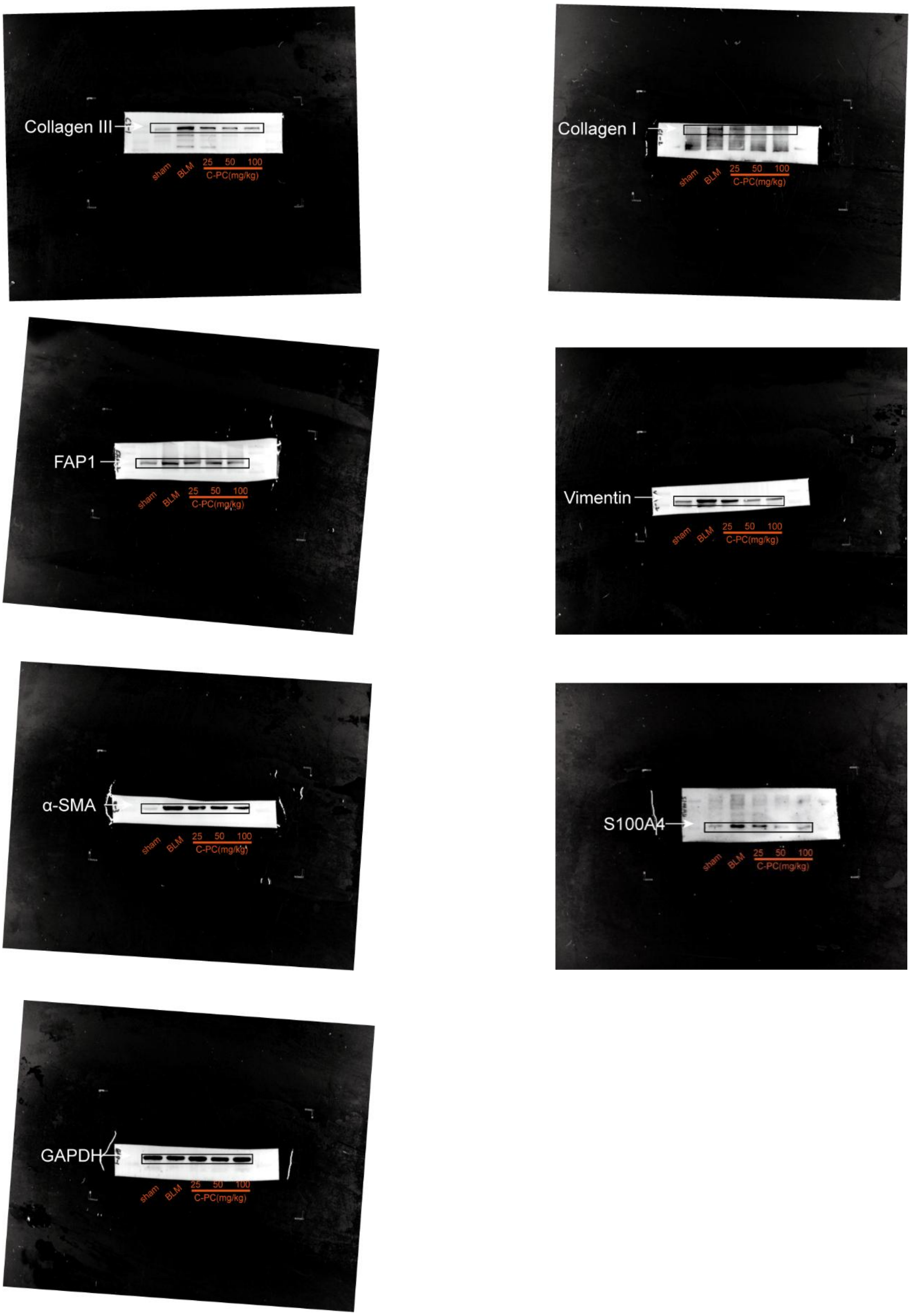
Collagen III
sham
BLM
25 50 100
C-PC(mg/kg)
Collagen I
sham
BLM
25 50 100
C-PC(mg/kg)
FAP1
sham
BLM
25 50 100
C-PC(mg/kg)
Vimentin
sham
BLM
25 50 100
C-PC(mg/kg)
α-SMA
sham
BLM
25 50 100
C-PC(mg/kg)
S100A4
sham
BLM
25 50 100
C-PC(mg/kg)
GAPDH
sham
BLM
25 50 100
C-PC(mg/kg)

Figure 3B-1：

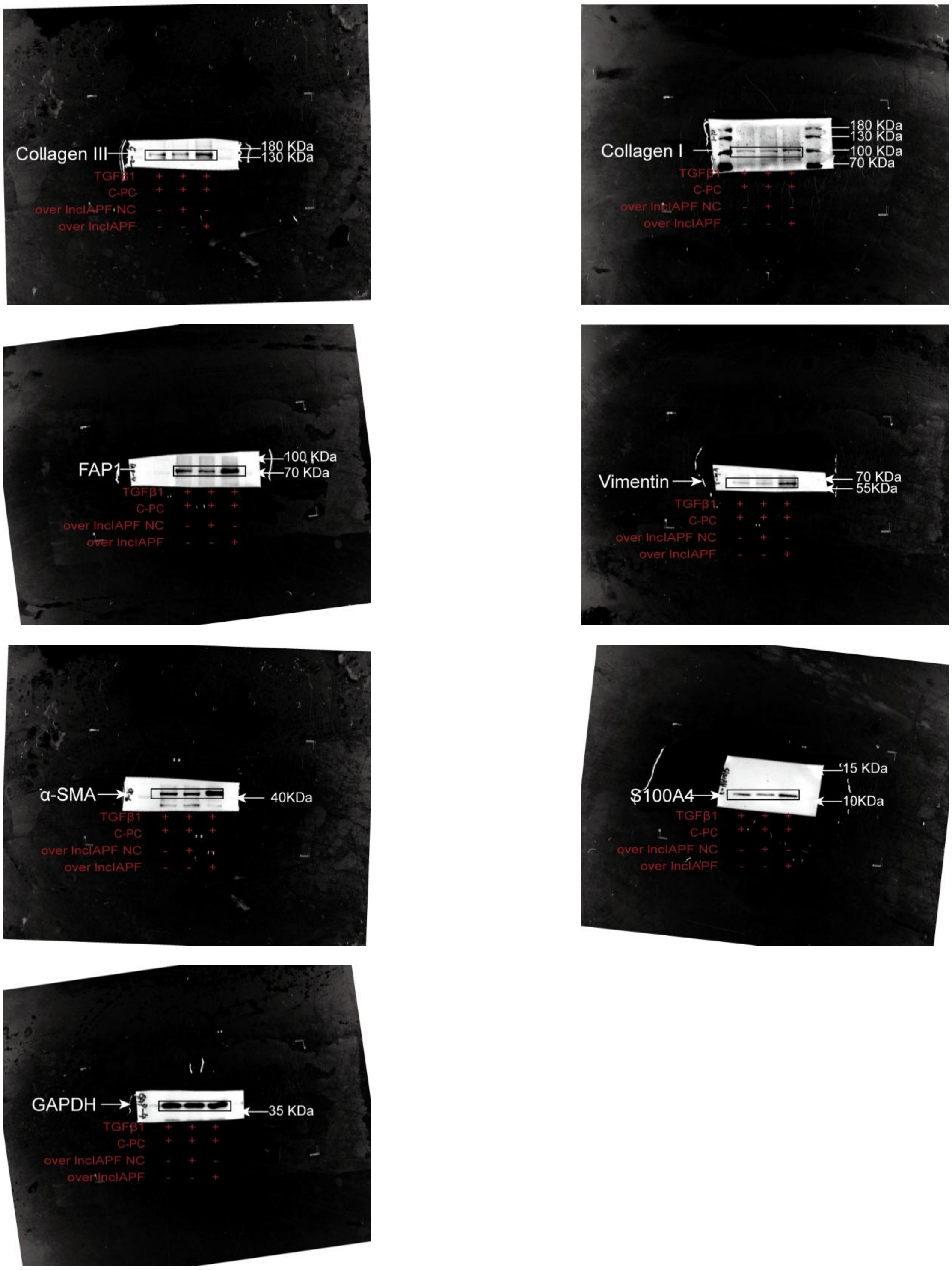

Collagen III
180 KDa
130 KDa
TGFβ1
C-PC
over lnclAPF NC
over lnclAPF
Collagen I
180 KDa
130 KDa
100 KDa
70 KDa
FAP1
100 KDa
70 KDa
Vimentin
70 KDa
55KDa
α-SMA
40KDa
S100A4
15 KDa
10KDa
GAPDH
35 KDa

Figure 3B-2：

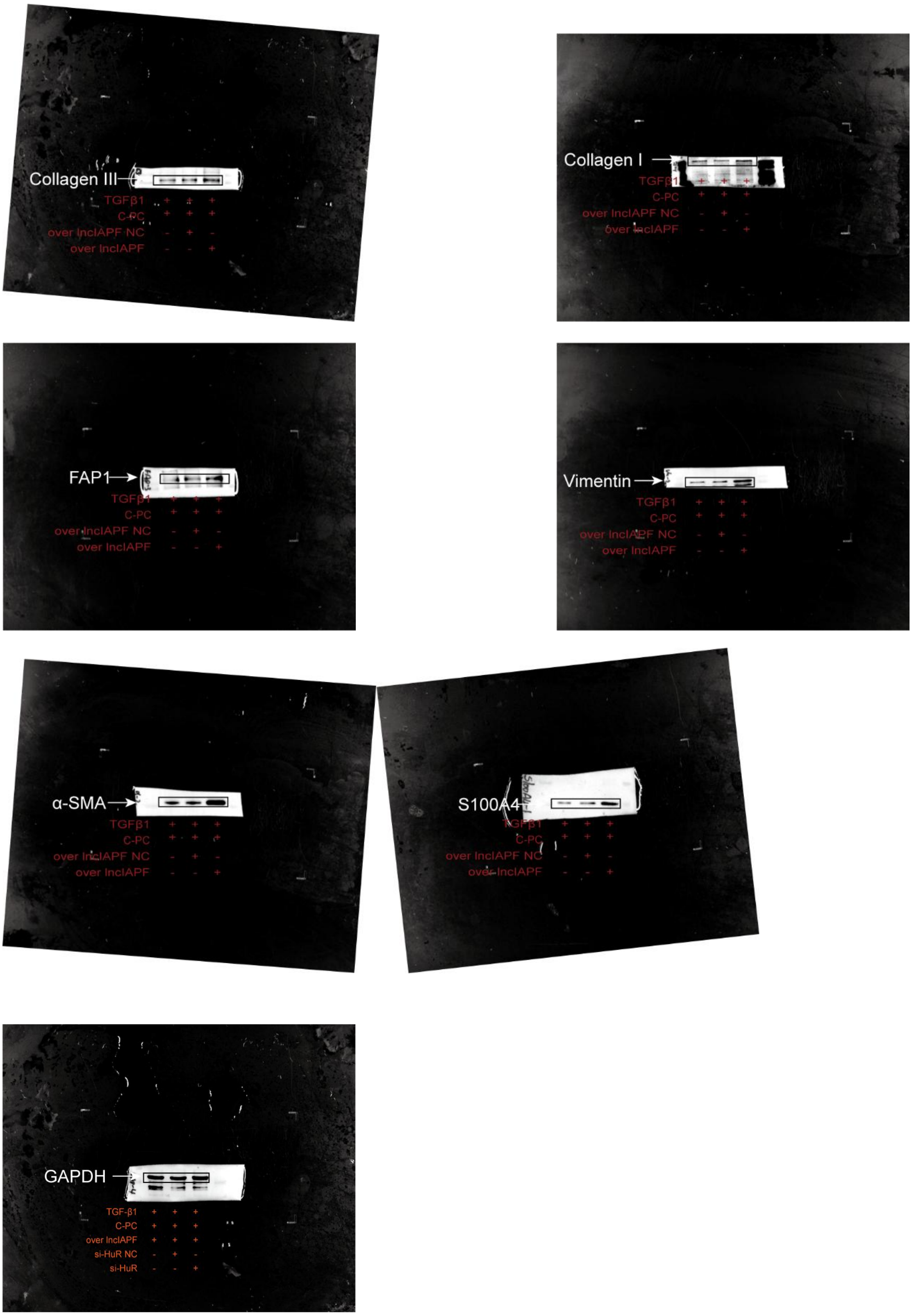
Collagen III
TGFβ1 + + +
C-PC + + +
over lnclAPF NC - + -
over lnclAPF - - +
Collagen I
TGFβ1 + + +
C-PC + + +
over lnclAPF NC - + -
over lnclAPF - - +
FAP1
TGFβ1 + + +
C-PC + + +
over lnclAPF NC - + -
over lnclAPF - - +
Vimentin
TGFβ1 + + +
C-PC + + +
over lnclAPF NC - + -
over lnclAPF - - +
α-SMA
TGFβ1 + + +
C-PC + + +
over lnclAPF NC - + -
over lnclAPF - - +
S100A4
TGFβ1 + + +
C-PC + + +
over lnclAPF NC - + -
over lnclAPF - - +
GAPDH
TGF-β1 + + +
C-PC + + +
over lnclAPF + + +
si-HuR NC - + -
si-HuR - - +

Figure 3B-3：

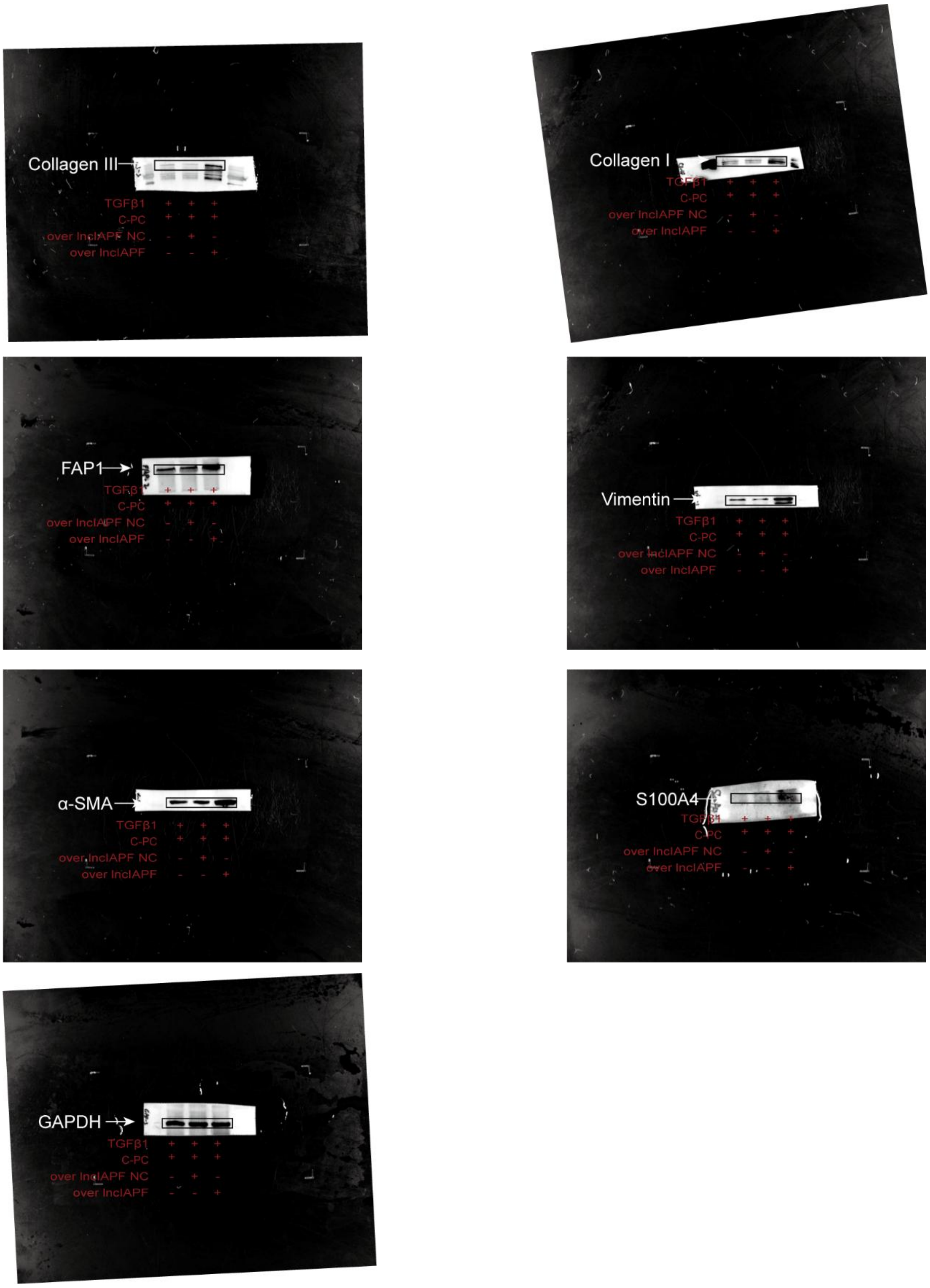
Collagen III
TGFβ1 + + +
C-PC + + +
over lncIAPF NC - + -
over lncIAPF - - +
Collagen I
TGFβ1 + + +
C-PC + + +
over lncIAPF NC - + -
over lncIAPF - - +
FAP1
TGFβ1 + + +
C-PC + + +
over lncIAPF NC - + -
over lncIAPF - - +
Vimentin
TGFβ1 + + +
C-PC + + +
over lncIAPF NC - + -
over lncIAPF - - +
α-SMA
TGFβ1 + + +
C-PC + + +
over lncIAPF NC - + -
over lncIAPF - - +
S100A4
TGFβ1 + + +
C-PC + + +
over lncIAPF NC - + -
over lncIAPF - - +
GAPDH
TGFβ1 + + +
C-PC + + +
over lncIAPF NC - + -
over lncIAPF - - +

Figure 4H-1:

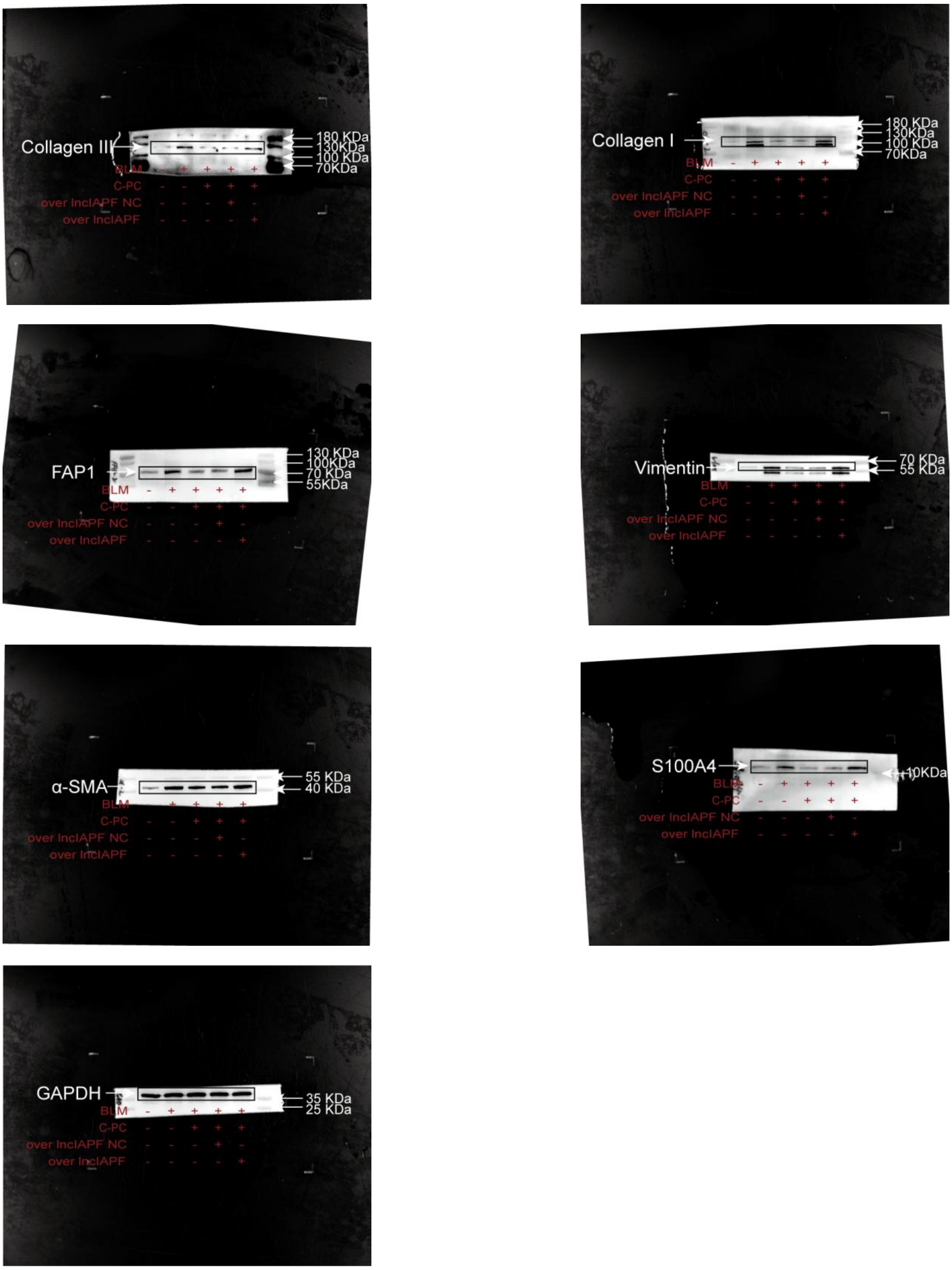
Collagen III
180 KDa
130KDa
100 KDa
70KDa
BLM
C-PC
over lncIAPF NC
over lncIAPF
Collagen I
180 KDa
130KDa
100 KDa
70KDa
BLM
C-PC
over lncIAPF NC
over lncIAPF
FAP1
130 KDa
100KDa
70 KDa
55KDa
BLM
C-PC
over lncIAPF NC
over lncIAPF
Vimentin
70 KDa
55 KDa
BLM
C-PC
over lncIAPF NC
over lncIAPF
α-SMA
55 KDa
40 KDa
BLM
C-PC
over lncIAPF NC
over lncIAPF
S100A4
10KDa
BLM
C-PC
over lncIAPF NC
over lncIAPF
GAPDH
35 KDa
25 KDa
BLM
C-PC
over lncIAPF NC
over lncIAPF

Figure 4H-2:

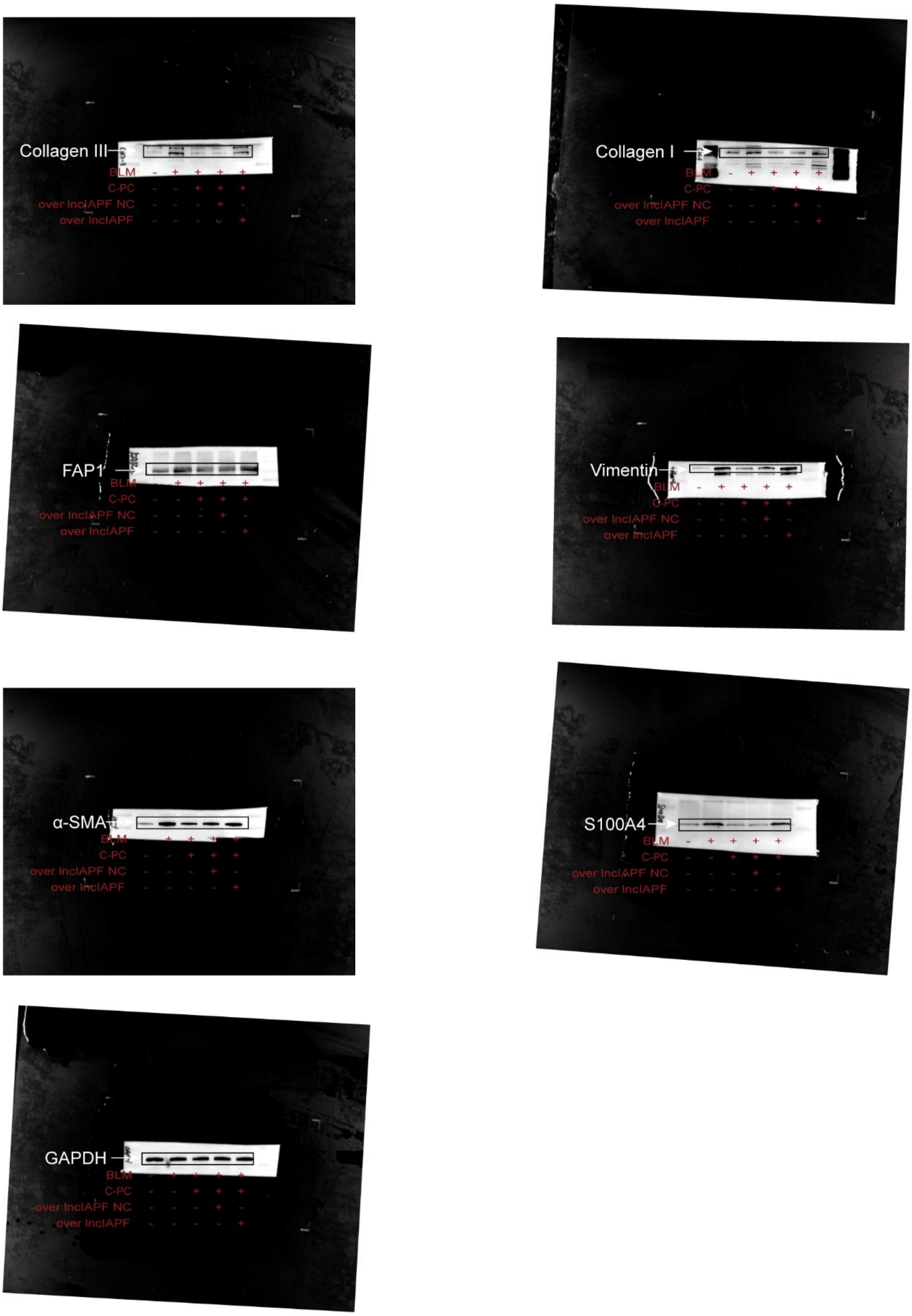
Collagen III
BLM - + + + +
C-PC - - + + +
over lnclAPF NC - - - + -
over lnclAPF - - - - +
Collagen I
BLM - + + + +
C-PC - - + + +
over lnclAPF NC - - - + -
over lnclAPF - - - - +
FAP1
BLM - + + + +
C-PC - - + + +
over lnclAPF NC - - - + -
over lnclAPF - - - - +
Vimentin
BLM - + + + +
C-PC - - + + +
over lnclAPF NC - - - + -
over lnclAPF - - - - +
α-SMA
BLM - + + + +
C-PC - - + + +
over lnclAPF NC - - - + -
over lnclAPF - - - - +
S100A4
BLM - + + + +
C-PC - - + + +
over lnclAPF NC - - - + -
over lnclAPF - - - - +
GAPDH
BLM - + + + +
C-PC - - + + +
over lnclAPF NC - - - + -
over lnclAPF - - - - +

Figure 4H-3:

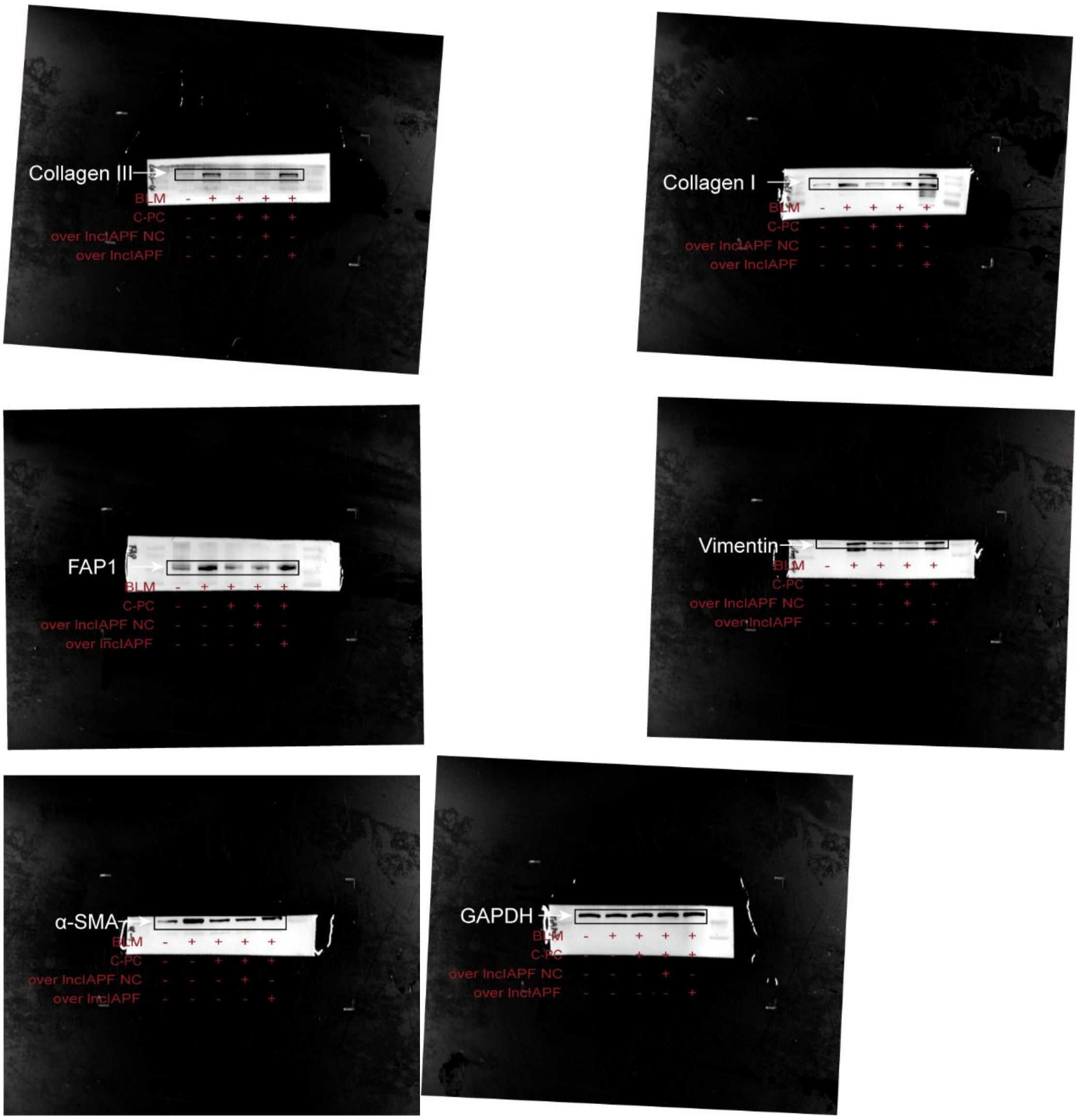
Collagen III
BLM - + + + +
C-PC - - + + +
over lnclAPF NC - - - + -
over lnclAPF - - - - +
Collagen I
BLM - + + + +
C-PC - - + + +
over lnclAPF NC - - - + -
over lnclAPF - - - - +
FAP1
BLM - + + + +
C-PC - - + + +
over lnclAPF NC - - - + -
over lnclAPF - - - - +
Vimentin
BLM - + + + +
C-PC - - + + +
over lnclAPF NC - - - + -
over lnclAPF - - - - +
α-SMA
BLM - + + + +
C-PC - - + + +
over lnclAPF NC - - - + -
over lnclAPF - - - - +
GAPDH
BLM - + + + +
C-PC - - + + +
over lnclAPF NC - - - + -
over lnclAPF - - - - +

Figure 5 E-1:

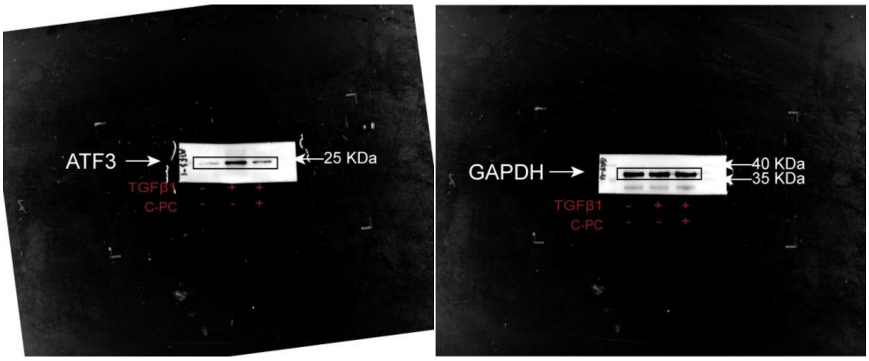

Figure 5 E-2:

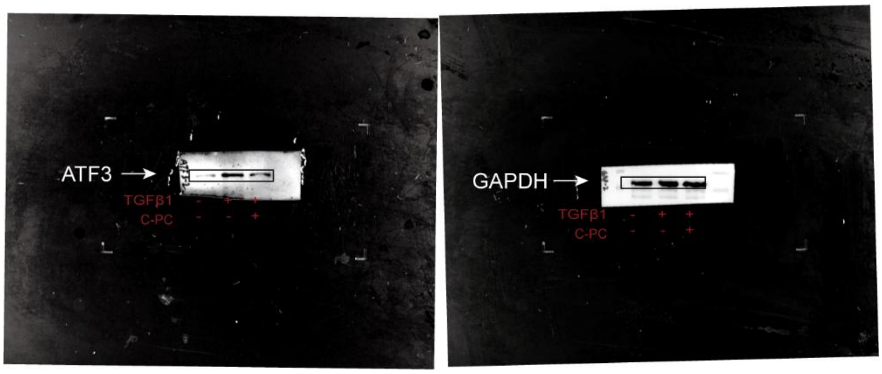

Figure 5 E-3:

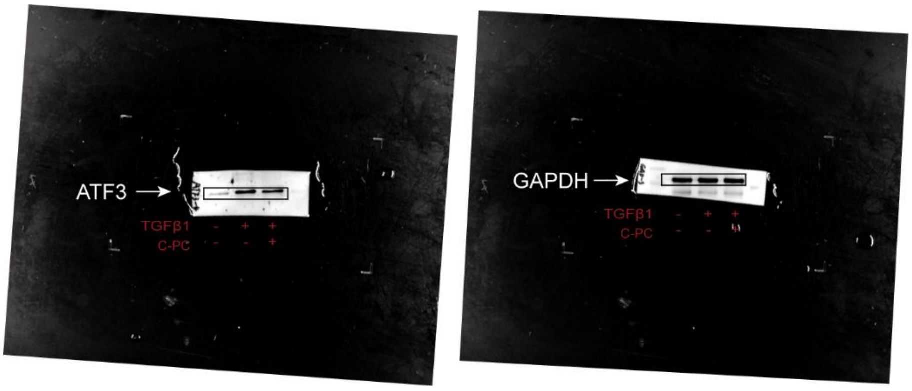

Figure 5 E-1:

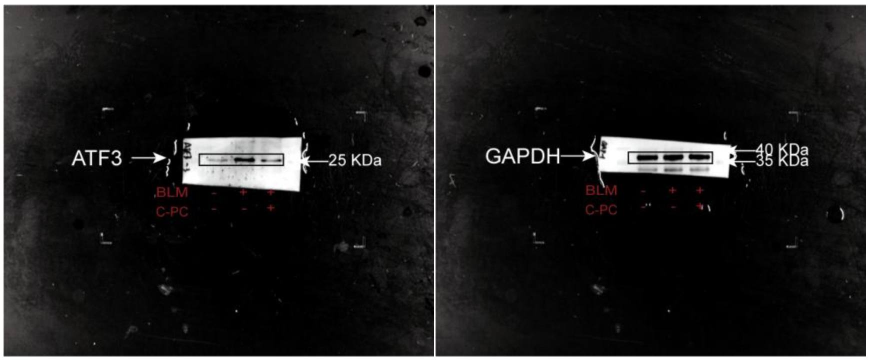

Figure 5 E-2:

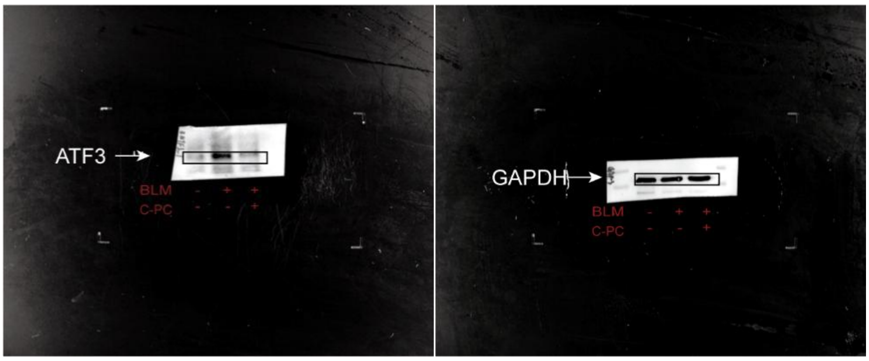

Figure 5 E-3:

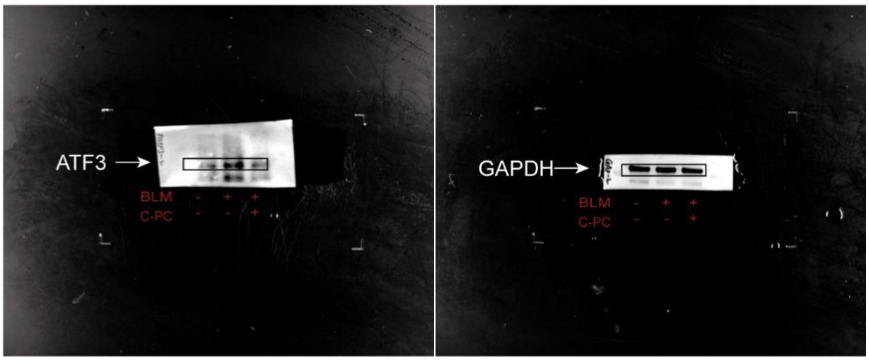

Figure 5F-1：

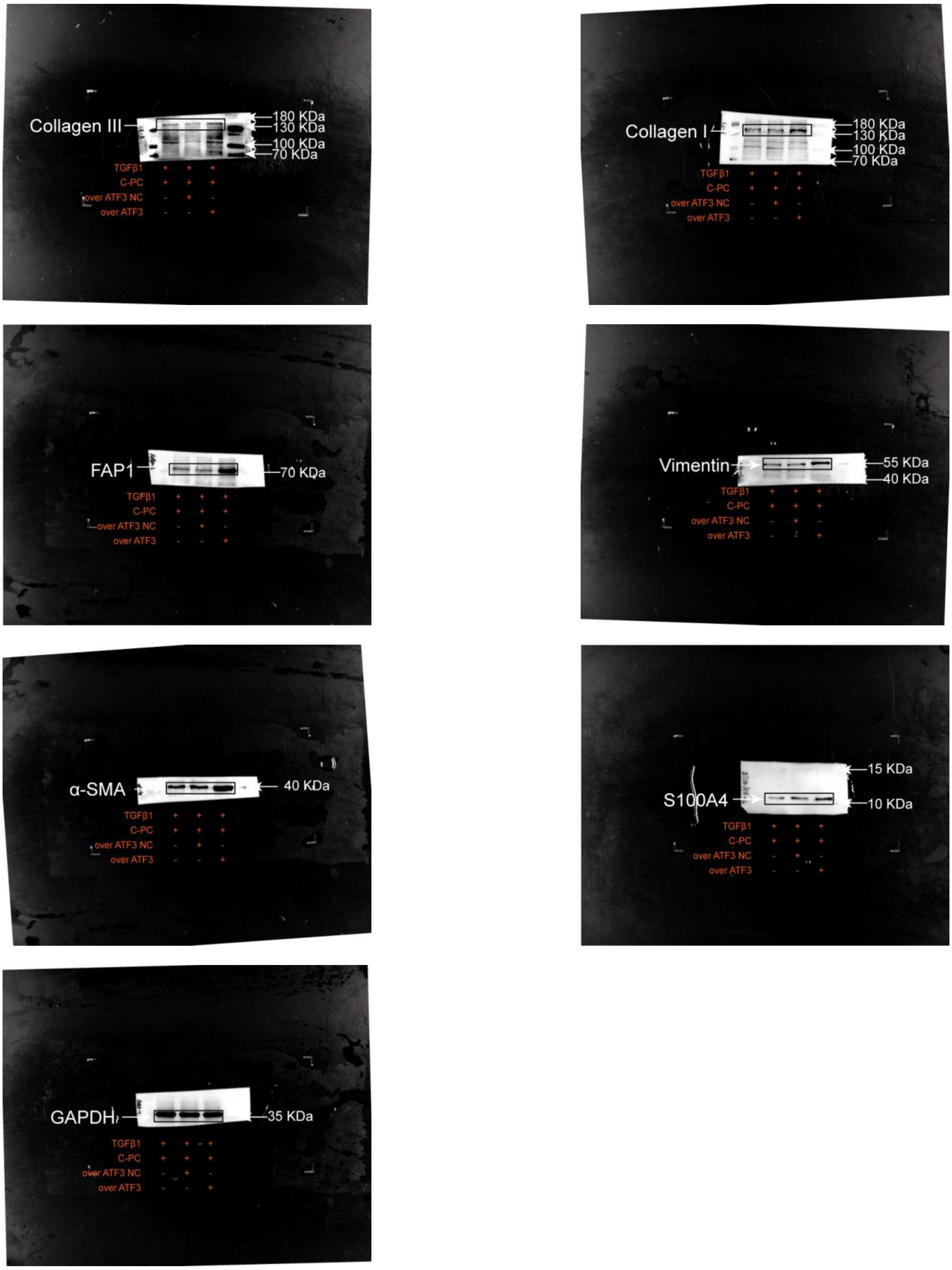
Collagen III
180 KDa
130 KDa
100 KDa
70 KDa
TGFβ1 + + +
C-PC + + +
over ATF3 NC - + -
over ATF3 - - +
Collagen I
180 KDa
130 KDa
100 KDa
70 KDa
TGFβ1 + + +
C-PC + + +
over ATF3 NC - + -
over ATF3 - - +
FAP1
70 KDa
TGFβ1 + + +
C-PC + + +
over ATF3 NC - + -
over ATF3 - - +
Vimentin
55 KDa
40 KDa
TGFβ1 + + +
C-PC + + +
over ATF3 NC - + -
over ATF3 - - +
α-SMA
40 KDa
TGFβ1 + + +
C-PC + + +
over ATF3 NC - + -
over ATF3 - - +
S100A4
15 KDa
10 KDa
TGFβ1 + + +
C-PC + + +
over ATF3 NC - + -
over ATF3 - - +
GAPDH
35 KDa
TGFβ1 + + +
C-PC + + +
over ATF3 NC - + -
over ATF3 - - +

Figure 5F-2:

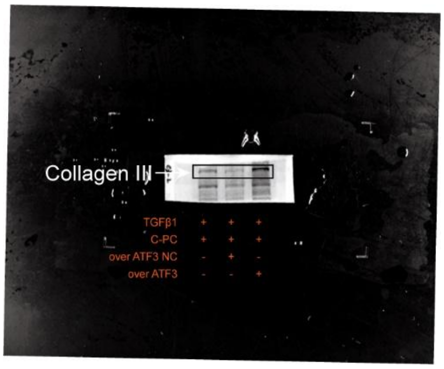
Collagen III
TGFβ1 + + +
C-PC + + +
over ATF3 NC - + -
over ATF3 - - +

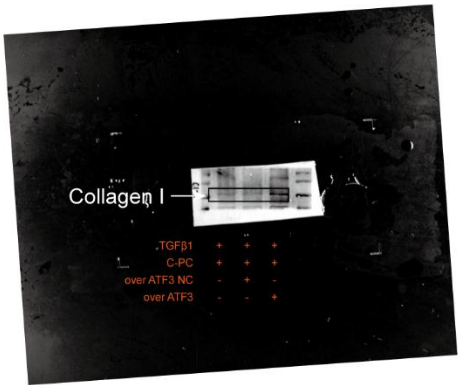
Collagen I
TGFβ1 + + +
C-PC + + +
over ATF3 NC - + -
over ATF3 - - +

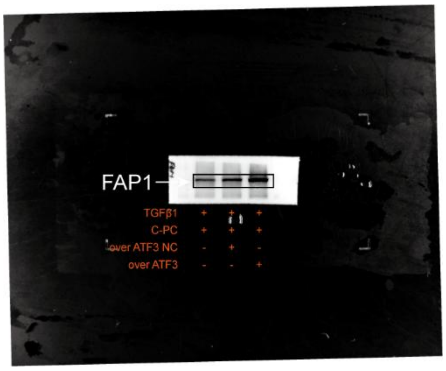
FAP1
TGFβ1 + + +
C-PC + + +
over ATF3 NC - + -
over ATF3 - - +

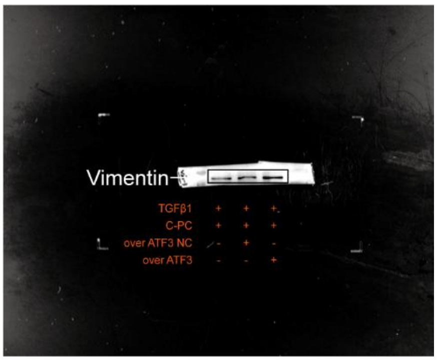
Vimentin
TGFβ1 + + +
C-PC + + +
over ATF3 NC - + -
over ATF3 - - +

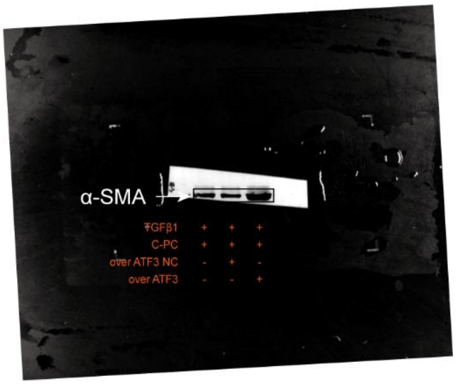
α-SMA
TGFβ1 + + +
C-PC + + +
over ATF3 NC - + -
over ATF3 - - +

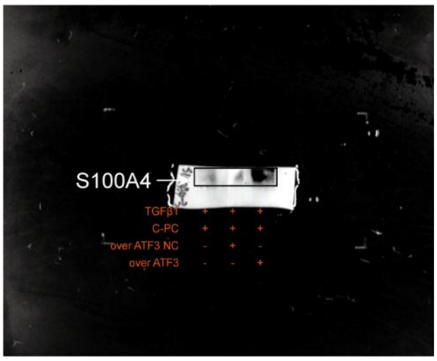
S100A4
TGFβ1 + + +
C-PC + + +
over ATF3 NC - + -
over ATF3 - - +

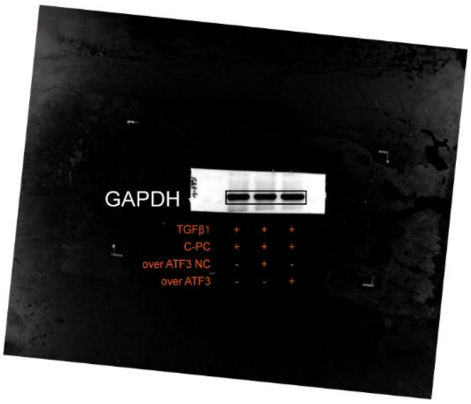
GAPDH
TGFβ1 + + +
C-PC + + +
over ATF3 NC - + -
over ATF3 - - +

Figure 5F-3：

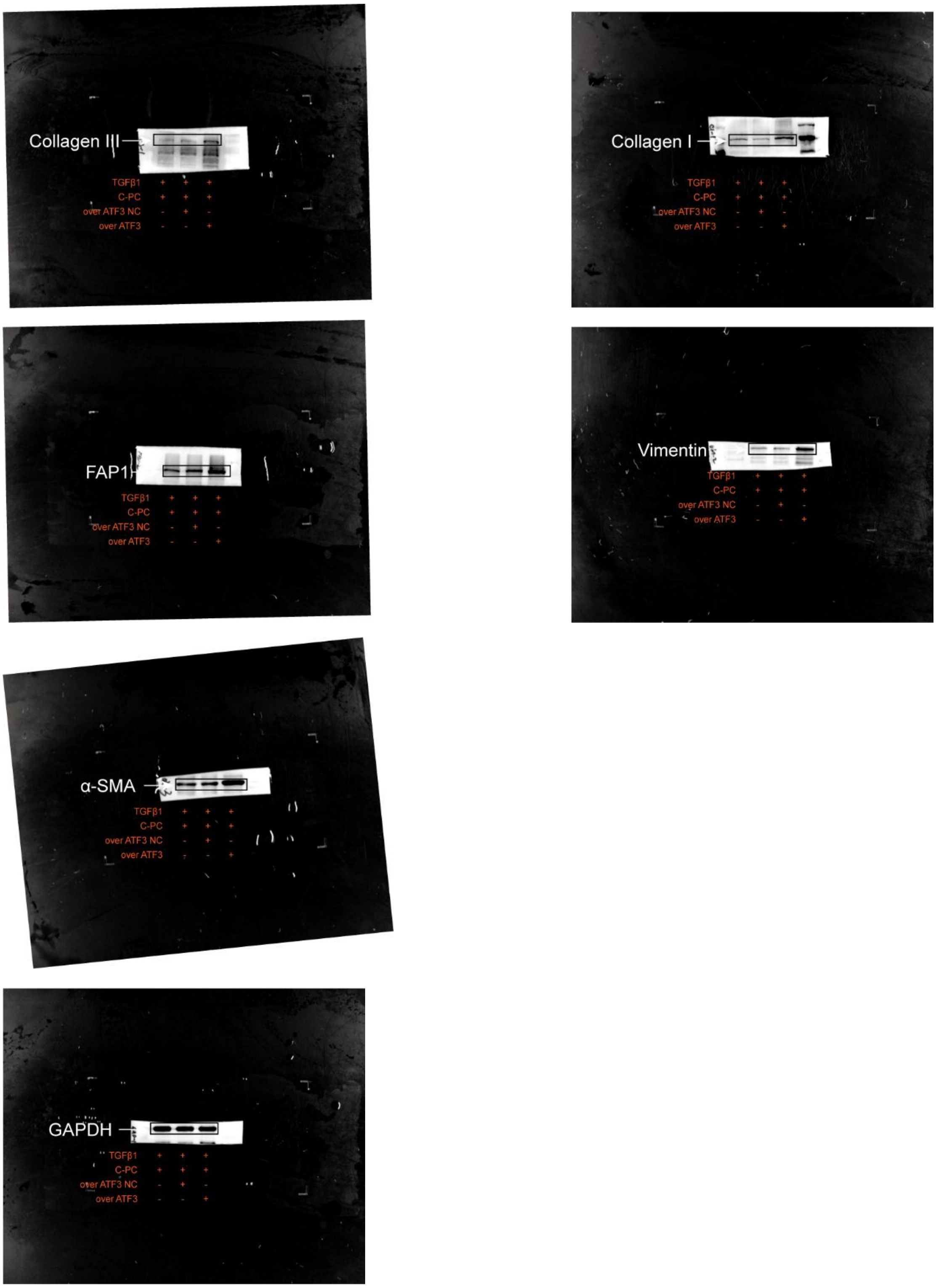
Collagen III
TGFβ1
C-PC
over ATF3 NC
over ATF3
Collagen I
TGFβ1
C-PC
over ATF3 NC
over ATF3
FAP1
TGFβ1
C-PC
over ATF3 NC
over ATF3
Vimentin
TGFβ1
C-PC
over ATF3 NC
over ATF3
α-SMA
TGFβ1
C-PC
over ATF3 NC
over ATF3
GAPDH
TGFβ1
C-PC
over ATF3 NC
over ATF3

Figure 5G-1:

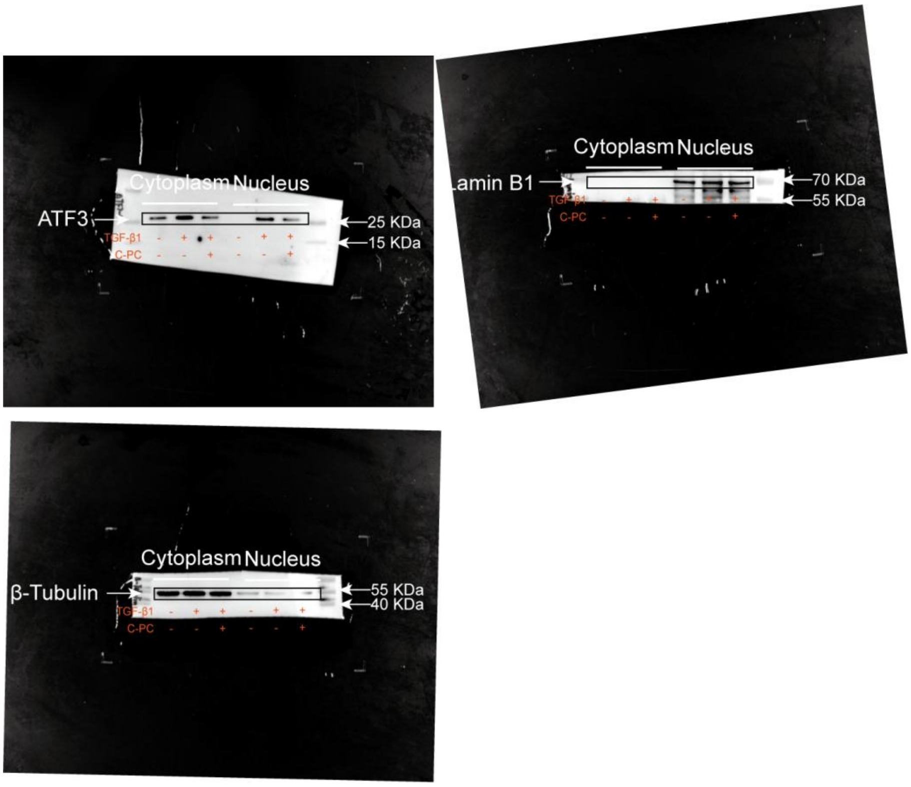

Figure 5G-2:

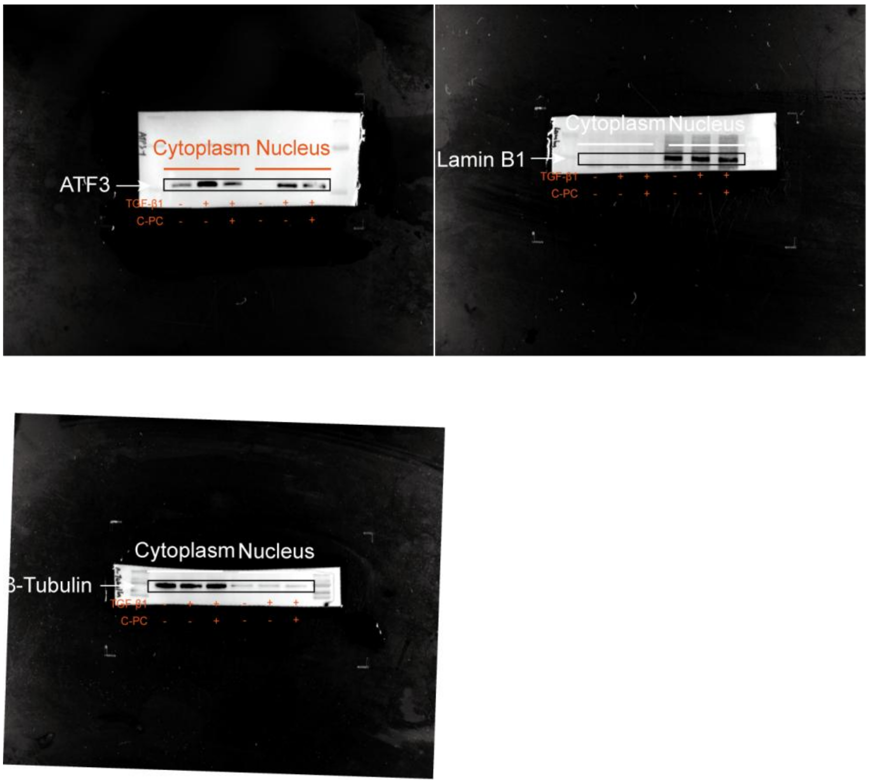

Figure 5I-1:

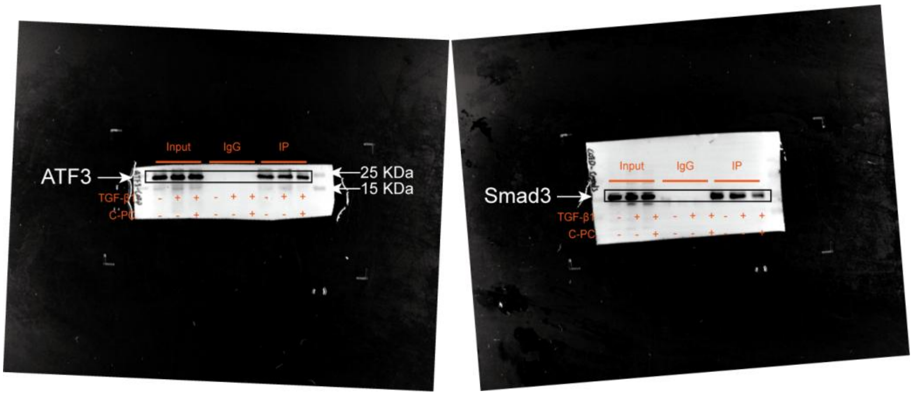

Figure 6B-1:

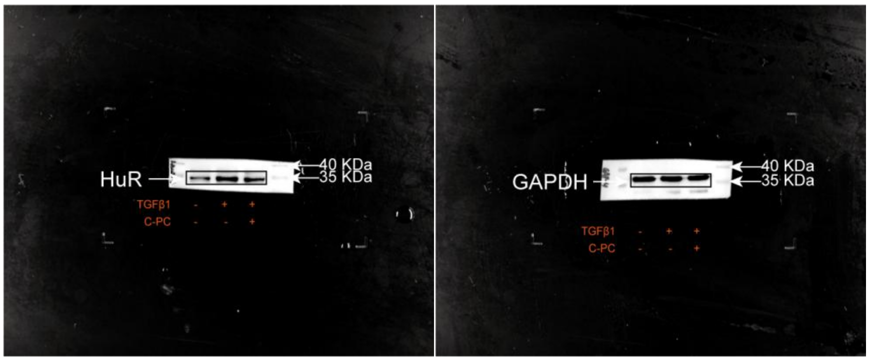

Figure 6B-2:

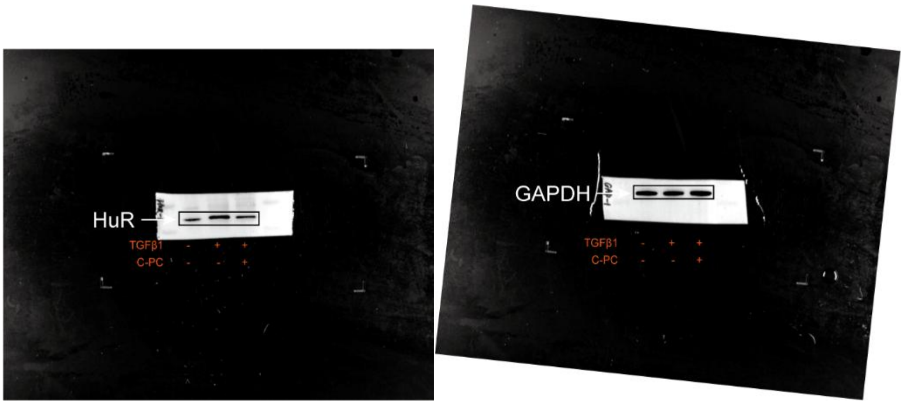

Figure 6B-3:

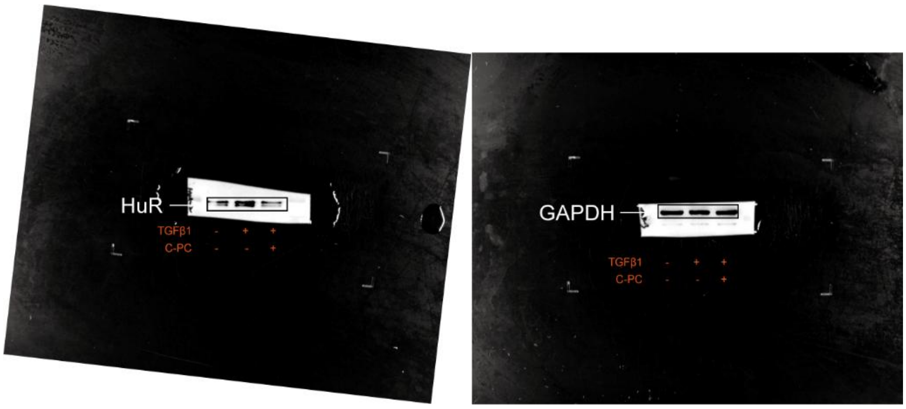

Figure 6B-1:

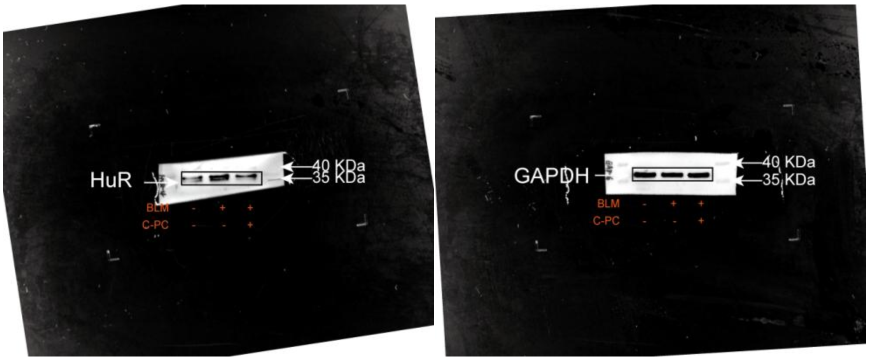

Figure 6B-2:

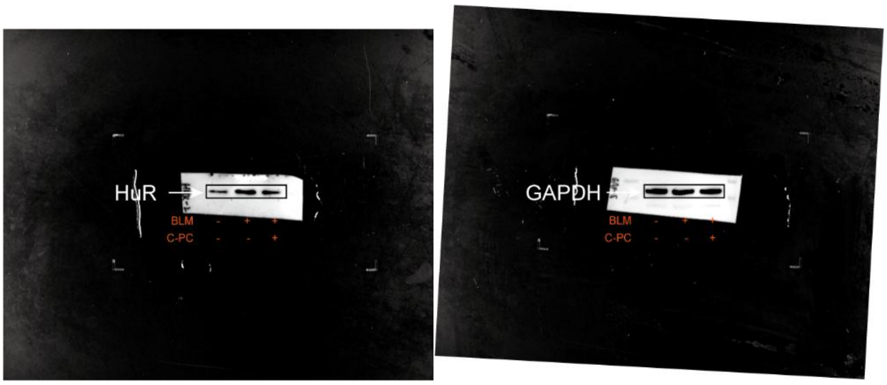

Figure 6B-3:

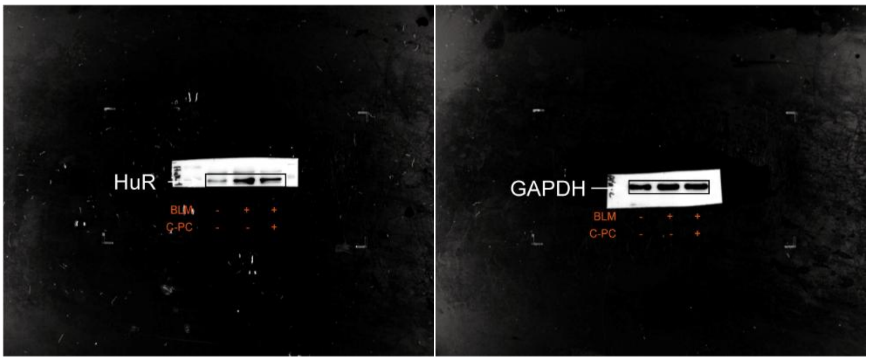

Figure 6C-1:

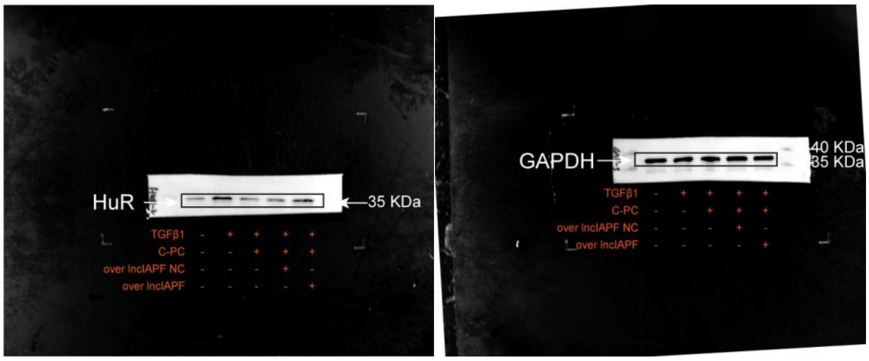

Figure 6C-2:

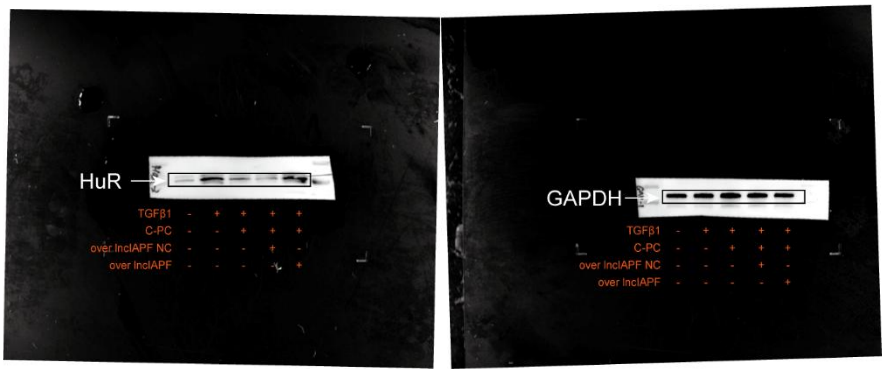

Figure 6C-3:

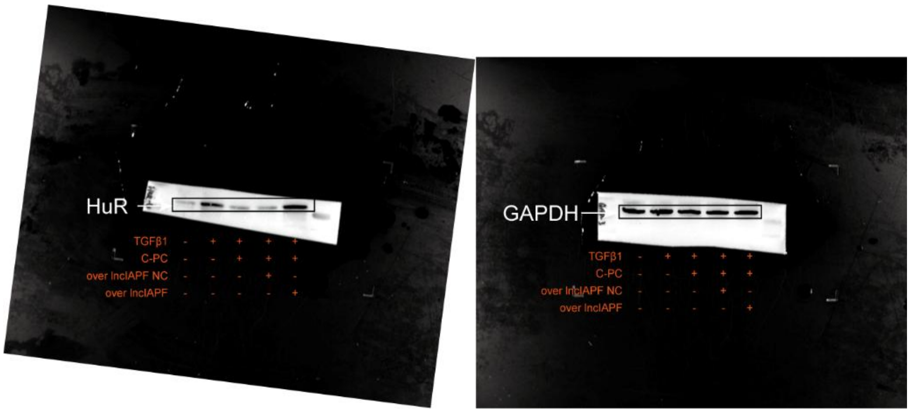

Figure 6C-1:

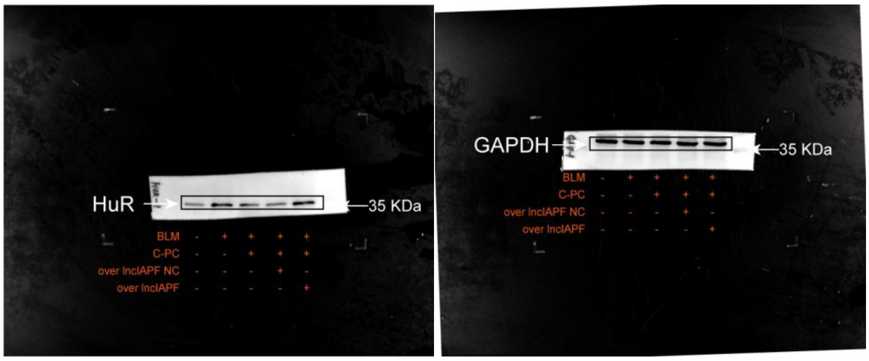

Figure 6C-2:

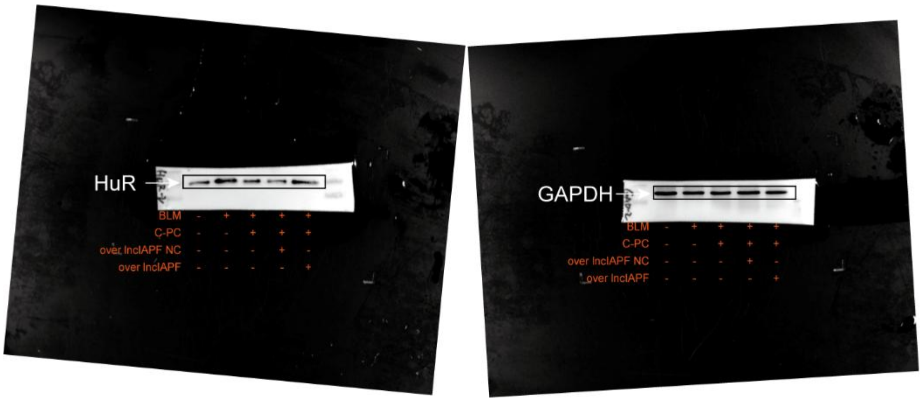

Figure 6C-3:

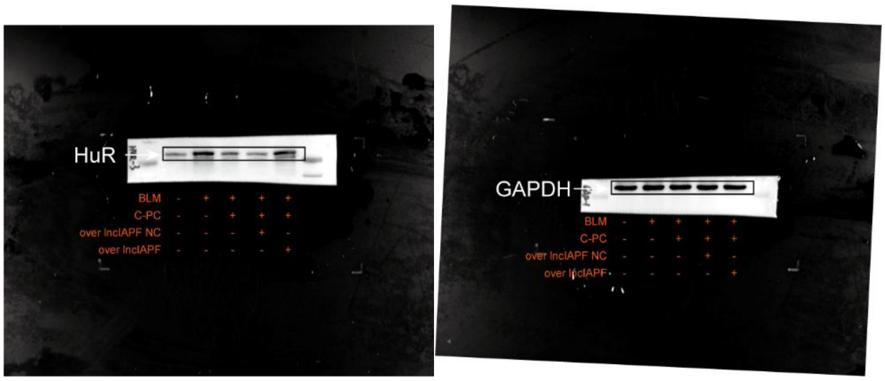

Figure 6D-1：

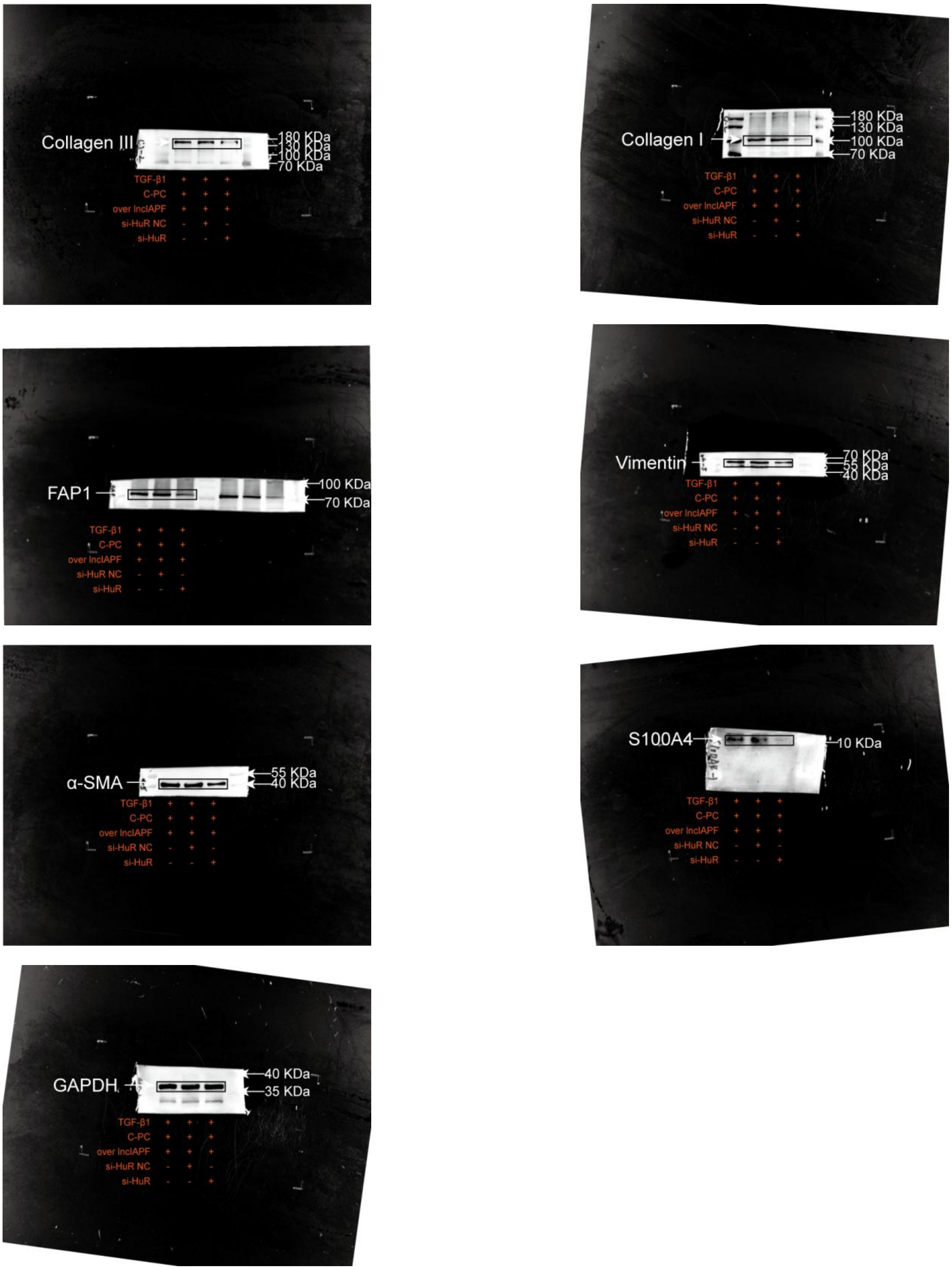
Collagen III
180 KDa
130 KDa
100 KDa
70 KDa
TGF-β1
C-PC
over lncIAPF
si-HuR NC
si-HuR
Collagen I
180 KDa
130 KDa
100 KDa
70 KDa
TGF-β1
C-PC
over lncIAPF
si-HuR NC
si-HuR
FAP1
100 KDa
70 KDa
TGF-β1
C-PC
over lncIAPF
si-HuR NC
si-HuR
Vimentin
70 KDa
55 KDa
40 KDa
TGF-β1
C-PC
over lncIAPF
si-HuR NC
si-HuR
α-SMA
55 KDa
40 KDa
TGF-β1
C-PC
over lncIAPF
si-HuR NC
si-HuR
S100A4
10 KDa
TGF-β1
C-PC
over lncIAPF
si-HuR NC
si-HuR
GAPDH
40 KDa
35 KDa
TGF-β1
C-PC
over lncIAPF
si-HuR NC
si-HuR

Figure 6D-2：

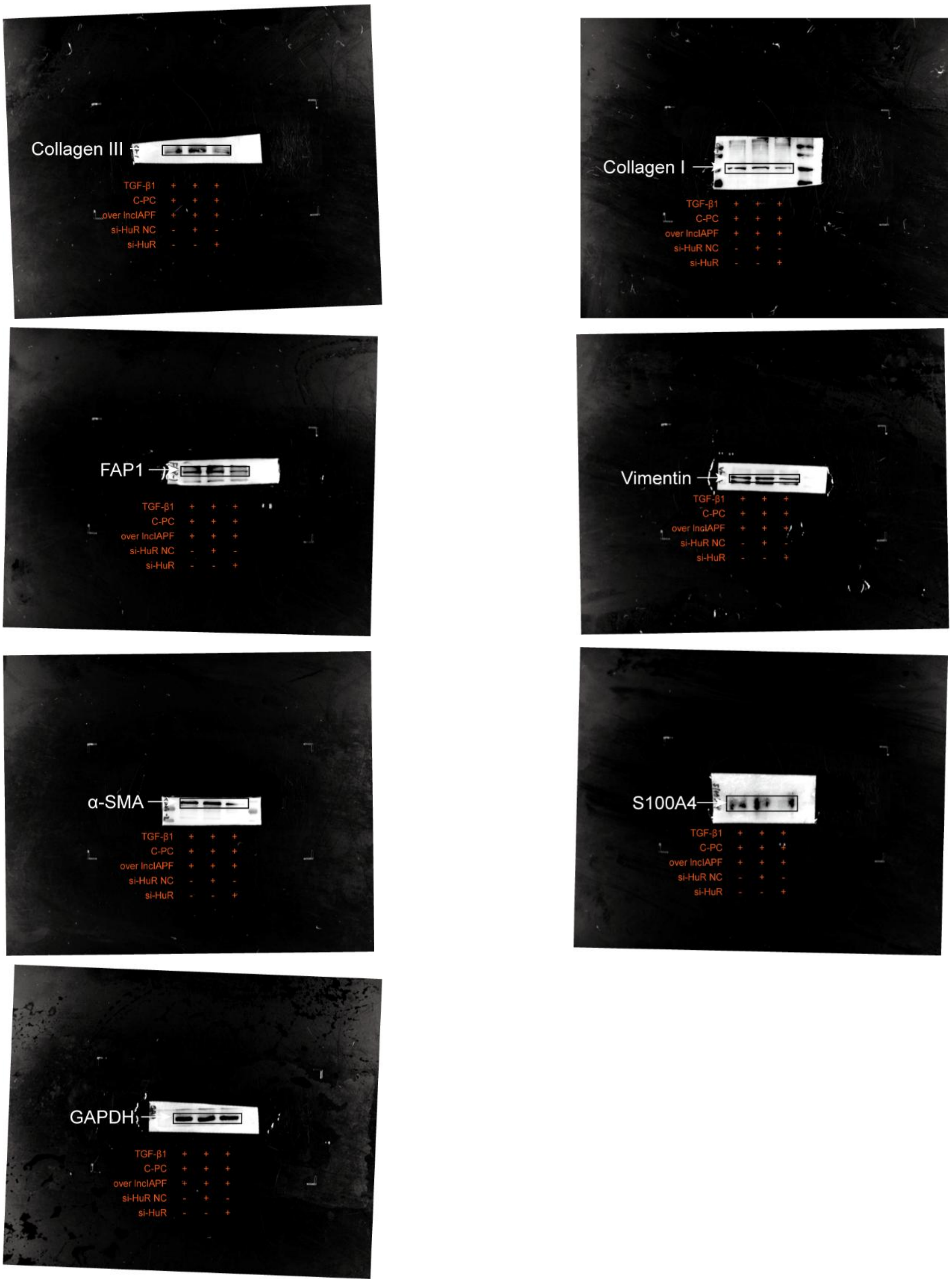
Collagen III
TGF-β1 + + +
C-PC + + +
over lnclAPF + + +
si-HuR NC - + -
si-HuR - - +
Collagen I
TGF-β1 + + +
C-PC + + +
over lnclAPF + + +
si-HuR NC - + -
si-HuR - - +
FAP1
TGF-β1 + + +
C-PC + + +
over lnclAPF + + +
si-HuR NC - + -
si-HuR - - +
Vimentin
TGF-β1 + + +
C-PC + + +
over lnclAPF + + +
si-HuR NC - + -
si-HuR - - +
α-SMA
TGF-β1 + + +
C-PC + + +
over lnclAPF + + +
si-HuR NC - + -
si-HuR - - +
S100A4
TGF-β1 + + +
C-PC + + +
over lnclAPF + + +
si-HuR NC - + -
si-HuR - - +
GAPDH
TGF-β1 + + +
C-PC + + +
over lnclAPF + + +
si-HuR NC - + -
si-HuR - - +

Figure 6D-3:

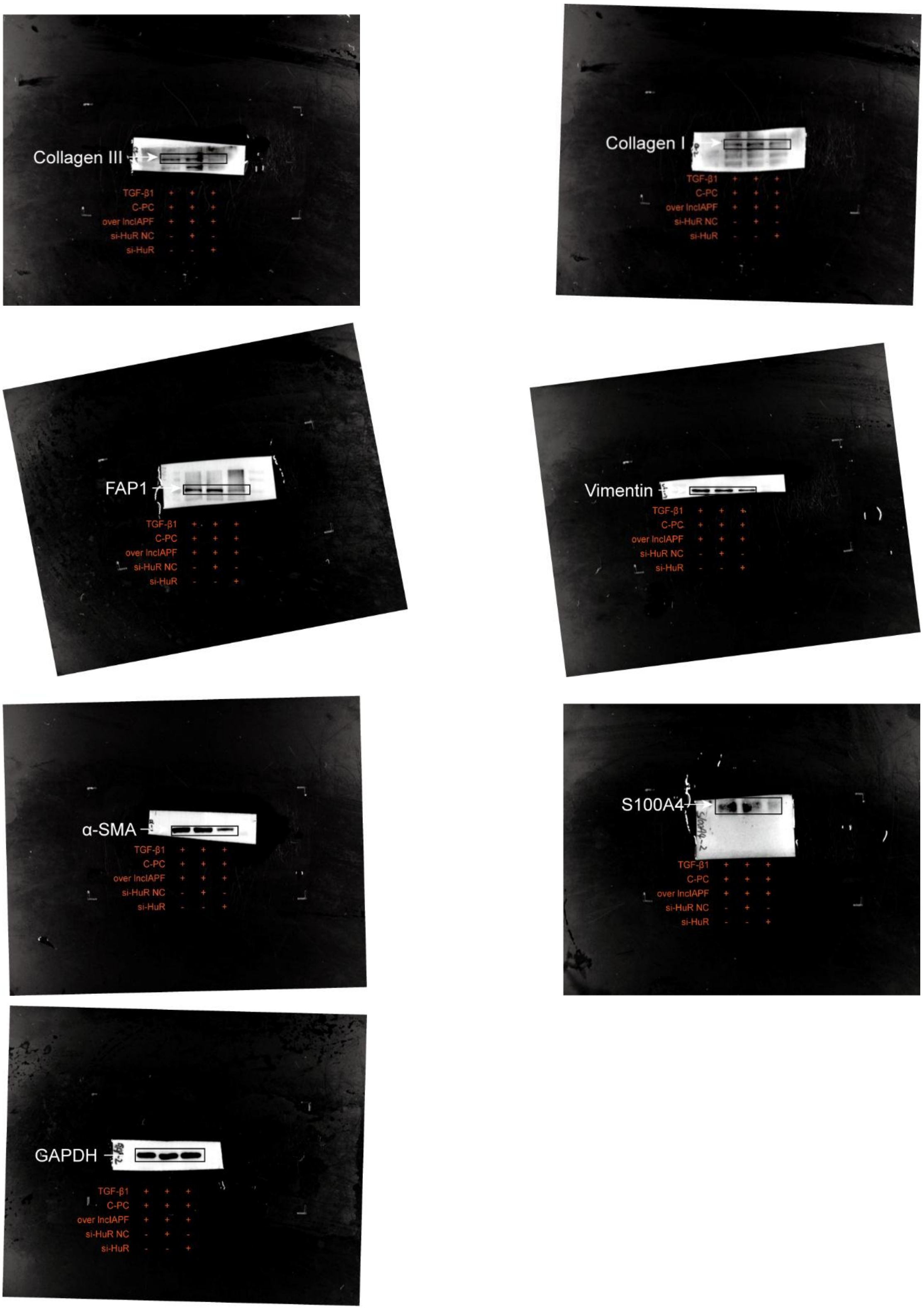
Collagen III
TGF-β1 + + +
C-PC + + +
over lncIAPF + + +
si-HuR NC - + -
si-HuR - - +
Collagen I
TGF-β1 + + +
C-PC + + +
over lncIAPF + + +
si-HuR NC - + -
si-HuR - - +
FAP1
TGF-β1 + + +
C-PC + + +
over lncIAPF + + +
si-HuR NC - + -
si-HuR - - +
Vimentin
TGF-β1 + + +
C-PC + + +
over lncIAPF + + +
si-HuR NC - + -
si-HuR - - +
α-SMA
TGF-β1 + + +
C-PC + + +
over lncIAPF + + +
si-HuR NC - + -
si-HuR - - +
S100A4
TGF-β1 + + +
C-PC + + +
over lncIAPF + + +
si-HuR NC - + -
si-HuR - - +
GAPDH
TGF-β1 + + +
C-PC + + +
over lncIAPF + + +
si-HuR NC - + -
si-HuR - - +

Figure 6E-1：

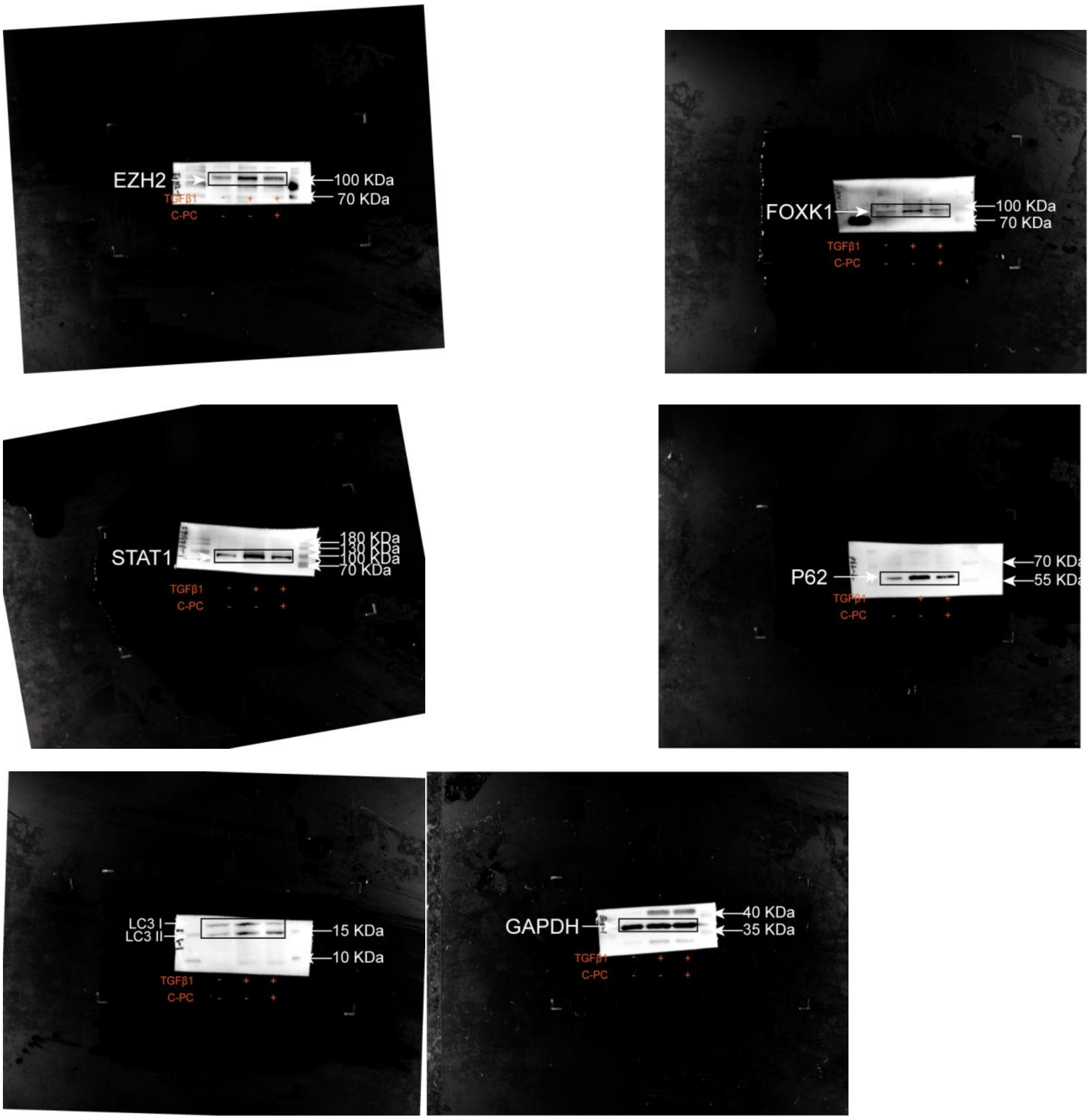

EZH2
100 KDa
70 KDa
TGFβ1 - + +
C-PC - - +
FOXK1
100 KDa
70 KDa
TGFβ1 - + +
C-PC - - +
STAT1
180 KDa
130 KDa
100 KDa
70 KDa
TGFβ1 - + +
C-PC - - +
P62
70 KDa
55 KDa
TGFβ1 - + +
C-PC - - +
LC3 I
LC3 II
15 KDa
10 KDa
TGFβ1 - + +
C-PC - - +
GAPDH
40 KDa
35 KDa
TGFβ1 - + +
C-PC - - +

Figure 6E-2：

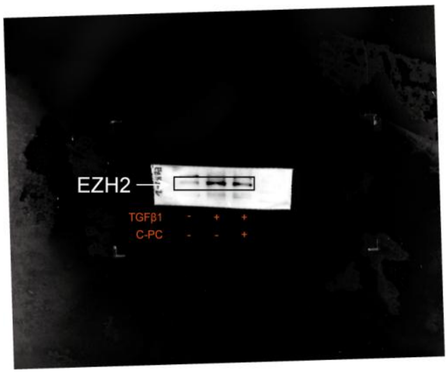
EZH2
TGFβ1 - + +
C-PC - - +

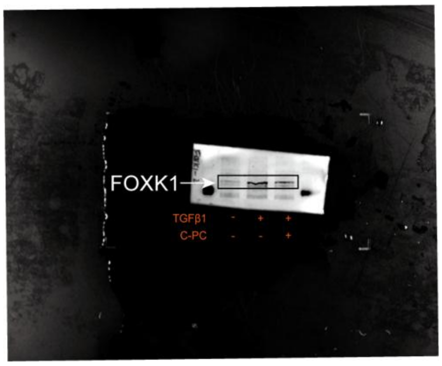
FOXK1
TGFβ1 - + +
C-PC - - +

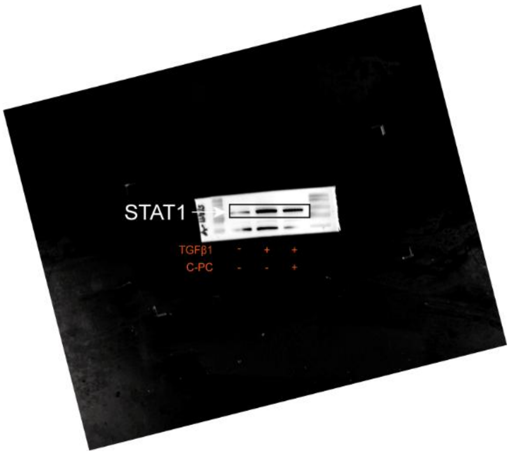
STAT1
TGFβ1 - + +
C-PC - - +

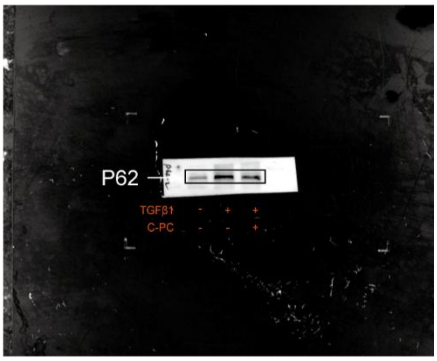
P62
TGFβ1 - + +
C-PC - - +

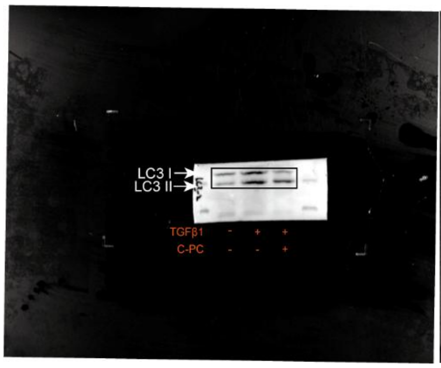
LC3 I
LC3 II
TGFβ1 - + +
C-PC - - +

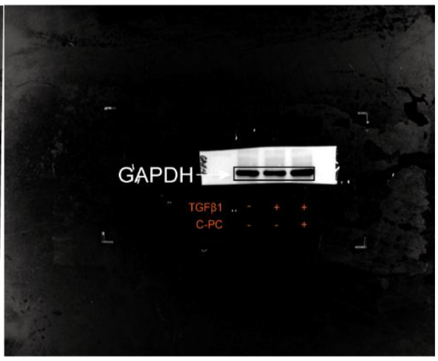
GAPDH
TGFβ1 - + +
C-PC - - +

Figure 6E-3：

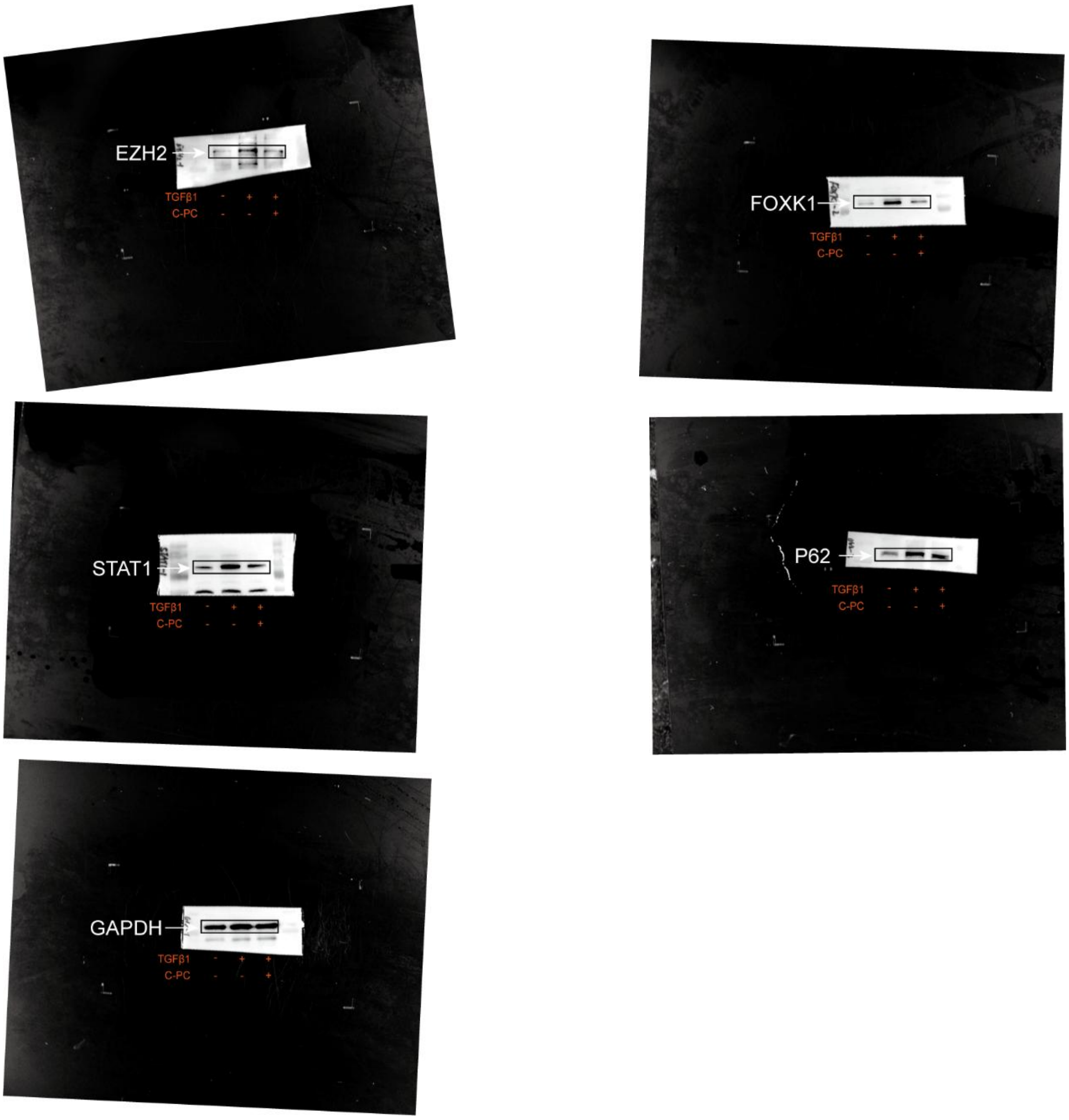
EZH2
TGFβ1 - + +
C-PC - - +
FOXK1
TGFβ1 - + +
C-PC - - +
STAT1
TGFβ1 - + +
C-PC - - +
P62
TGFβ1 - + +
C-PC - - +
GAPDH
TGFβ1 - + +
C-PC - - +

Figure 6F-1：

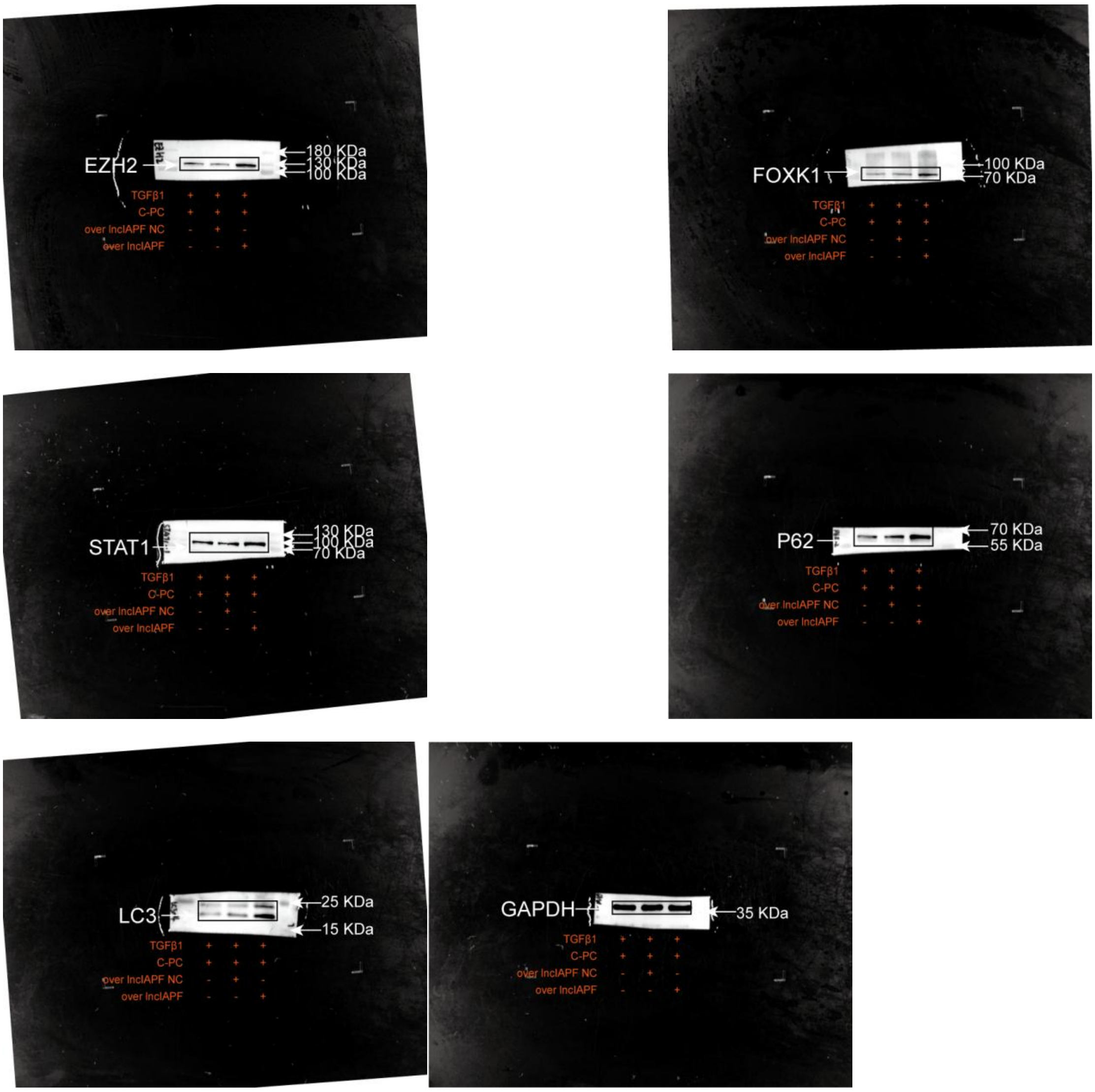
EZH2
180 KDa
130 KDa
100 KDa
TGFβ1 + + +
C-PC + + +
over lnclAPF NC - + -
over lnclAPF - - +
FOXK1
100 KDa
70 KDa
TGFβ1 + + +
C-PC + + +
over lnclAPF NC - + -
over lnclAPF - - +
STAT1
130 KDa
100 KDa
70 KDa
TGFβ1 + + +
C-PC + + +
over lnclAPF NC - + -
over lnclAPF - - +
P62
70 KDa
55 KDa
TGFβ1 + + +
C-PC + + +
over lnclAPF NC - + -
over lnclAPF - - +
LC3
25 KDa
15 KDa
TGFβ1 + + +
C-PC + + +
over lnclAPF NC - + -
over lnclAPF - - +
GAPDH
35 KDa
TGFβ1 + + +
C-PC + + +
over lnclAPF NC - + -
over lnclAPF - - +

Figure 6F-2：

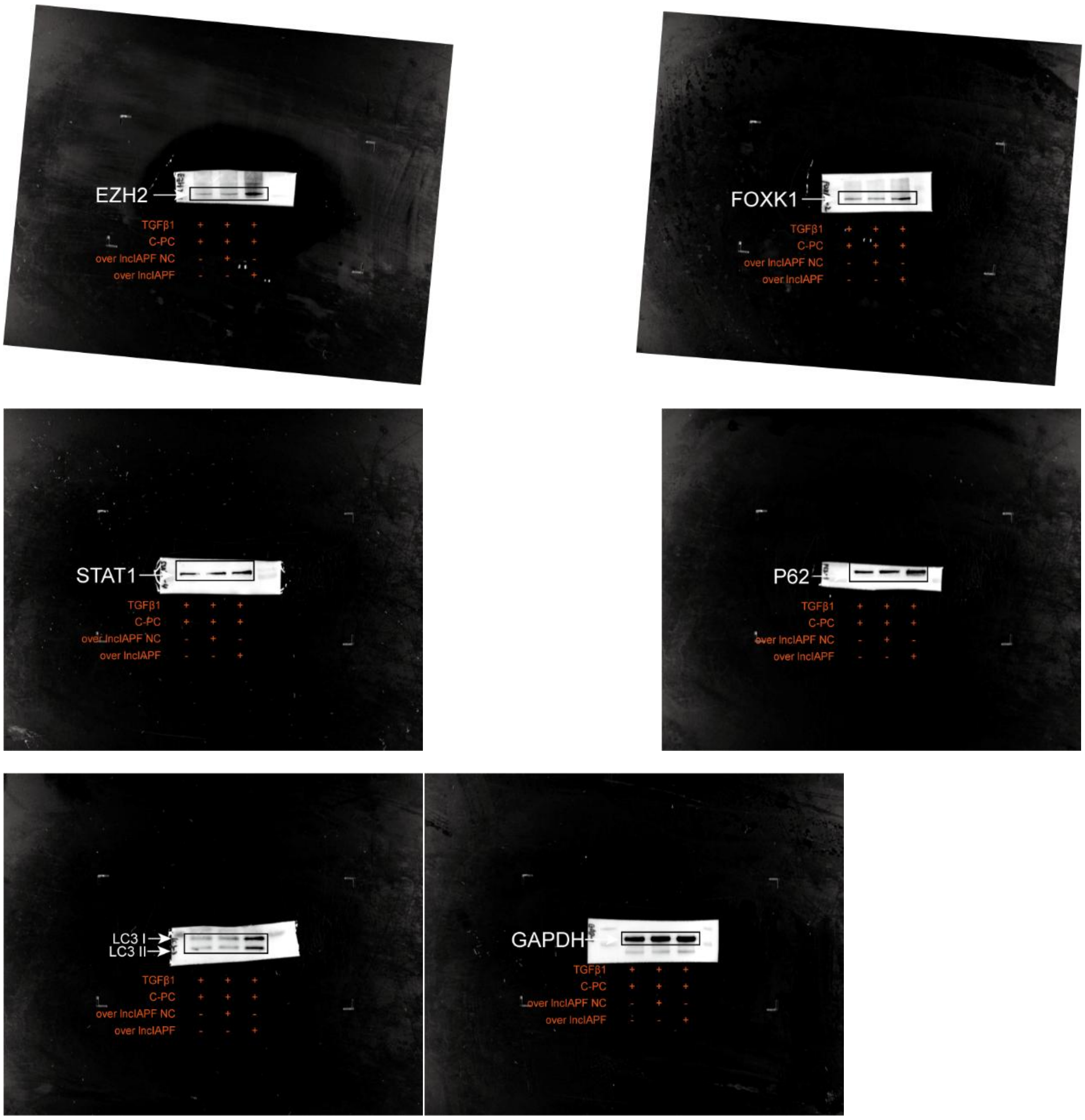
EZH2
FOXK1
STAT1
P62
LC3 I
LC3 II
GAPDH
TGFβ1
C-PC
over lncIAPF NC
over lncIAPF

Figure 6F-3：

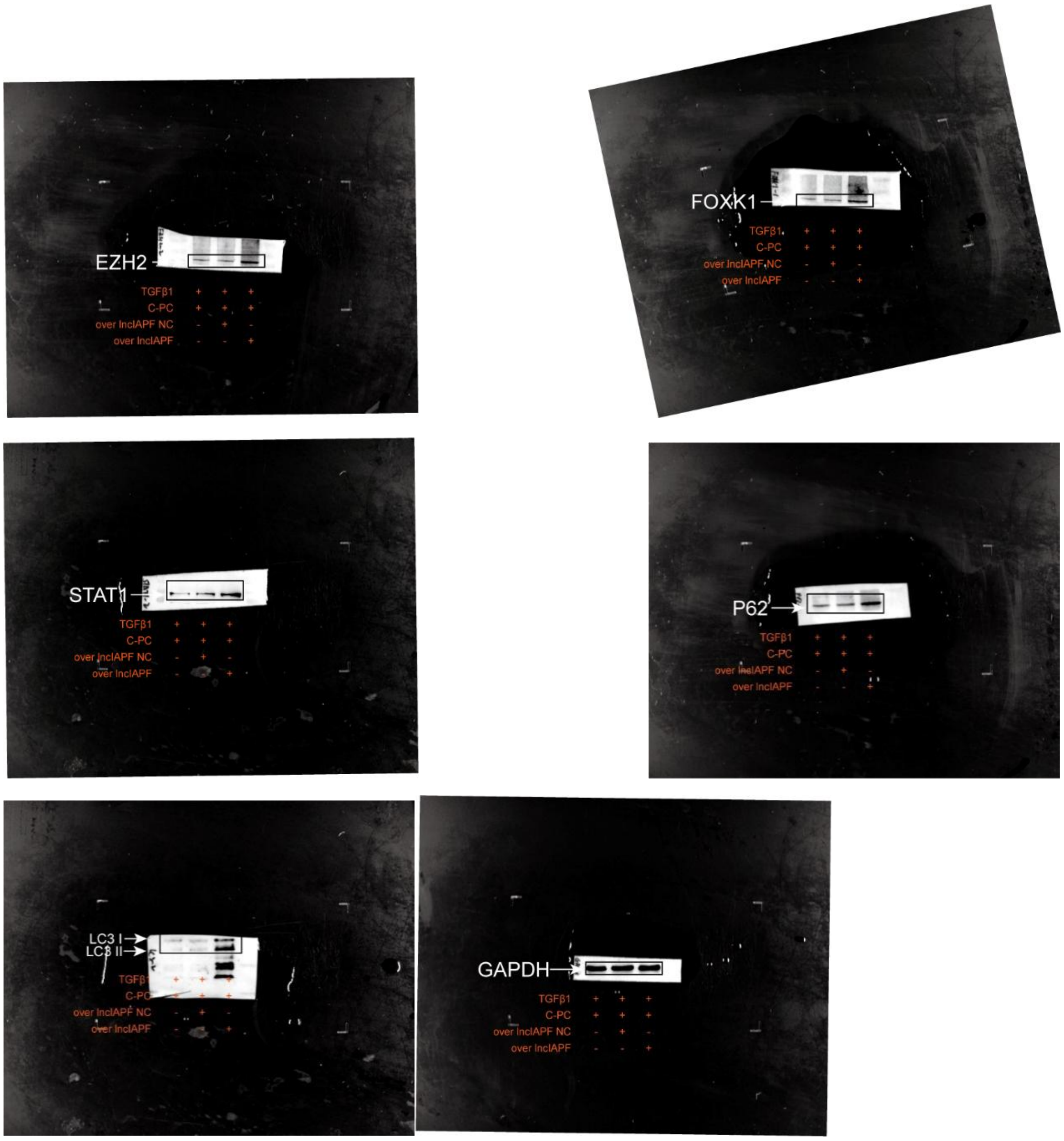
EZH2
TGFβ1
C-PC
over lncIAPF NC
over lncIAPF
FOXK1
STAT1
P62
LC3 I
LC3 II
GAPDH

Figure 6G-1：

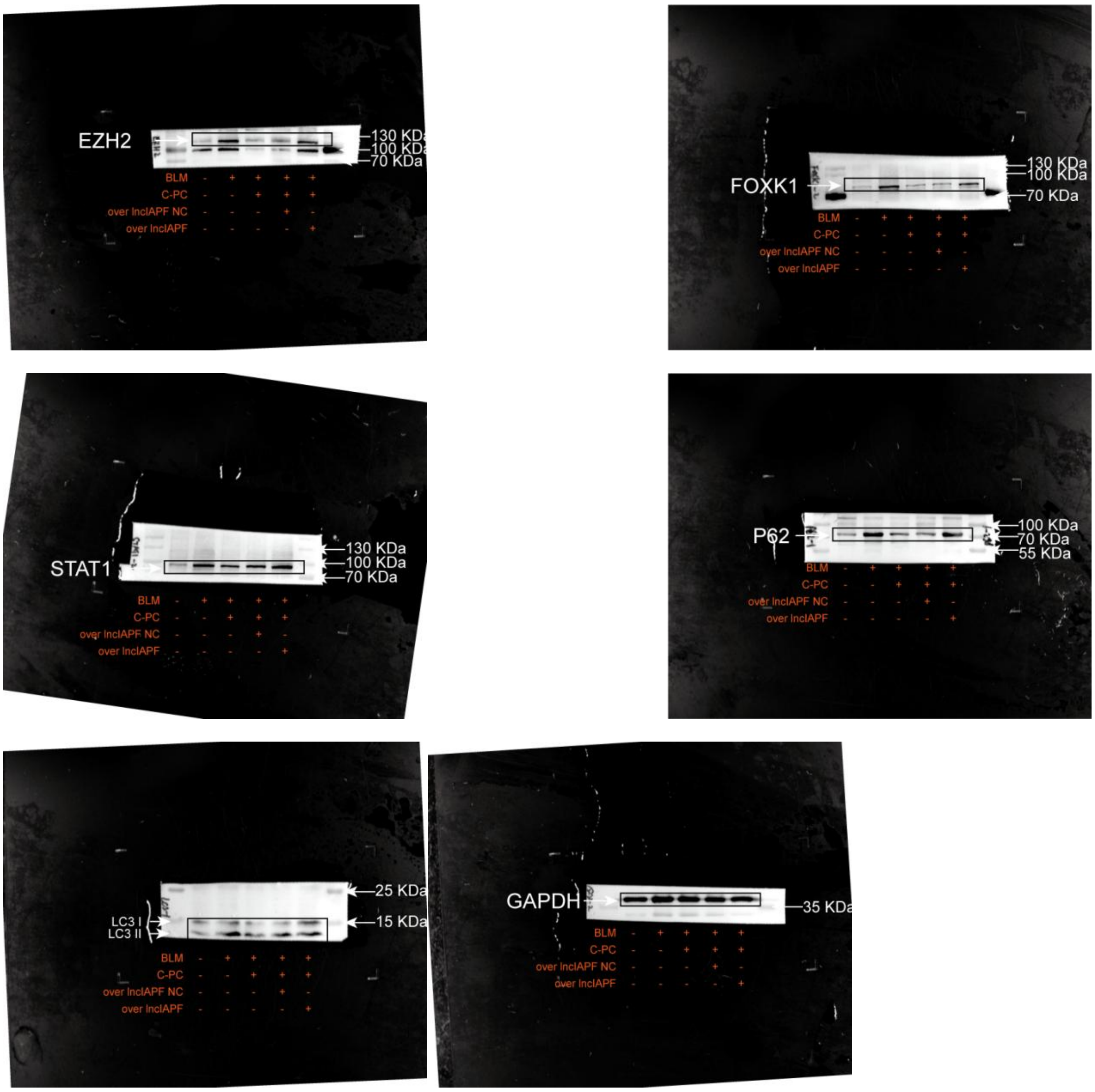
EZH2
130 KDa
100 KDa
70 KDa
BLM - + + + +
C-PC - - + + +
over lncIAPF NC - - - + -
over lncIAPF - - - - +
FOXK1
130 KDa
100 KDa
70 KDa
BLM - + + + +
C-PC - - + + +
over lncIAPF NC - - - + -
over lncIAPF - - - - +
STAT1
130 KDa
100 KDa
70 KDa
BLM - + + + +
C-PC - - + + +
over lncIAPF NC - - - + -
over lncIAPF - - - - +
P62
100 KDa
70 KDa
55 KDa
BLM - + + + +
C-PC - - + + +
over lncIAPF NC - - - + -
over lncIAPF - - - - +
LC3 I
LC3 II
25 KDa
15 KDa
BLM - + + + +
C-PC - - + + +
over lncIAPF NC - - - + -
over lncIAPF - - - - +
GAPDH
35 KDa
BLM - + + + +
C-PC - - + + +
over lncIAPF NC - - - + -
over lncIAPF - - - - +

Figure 6G-2:

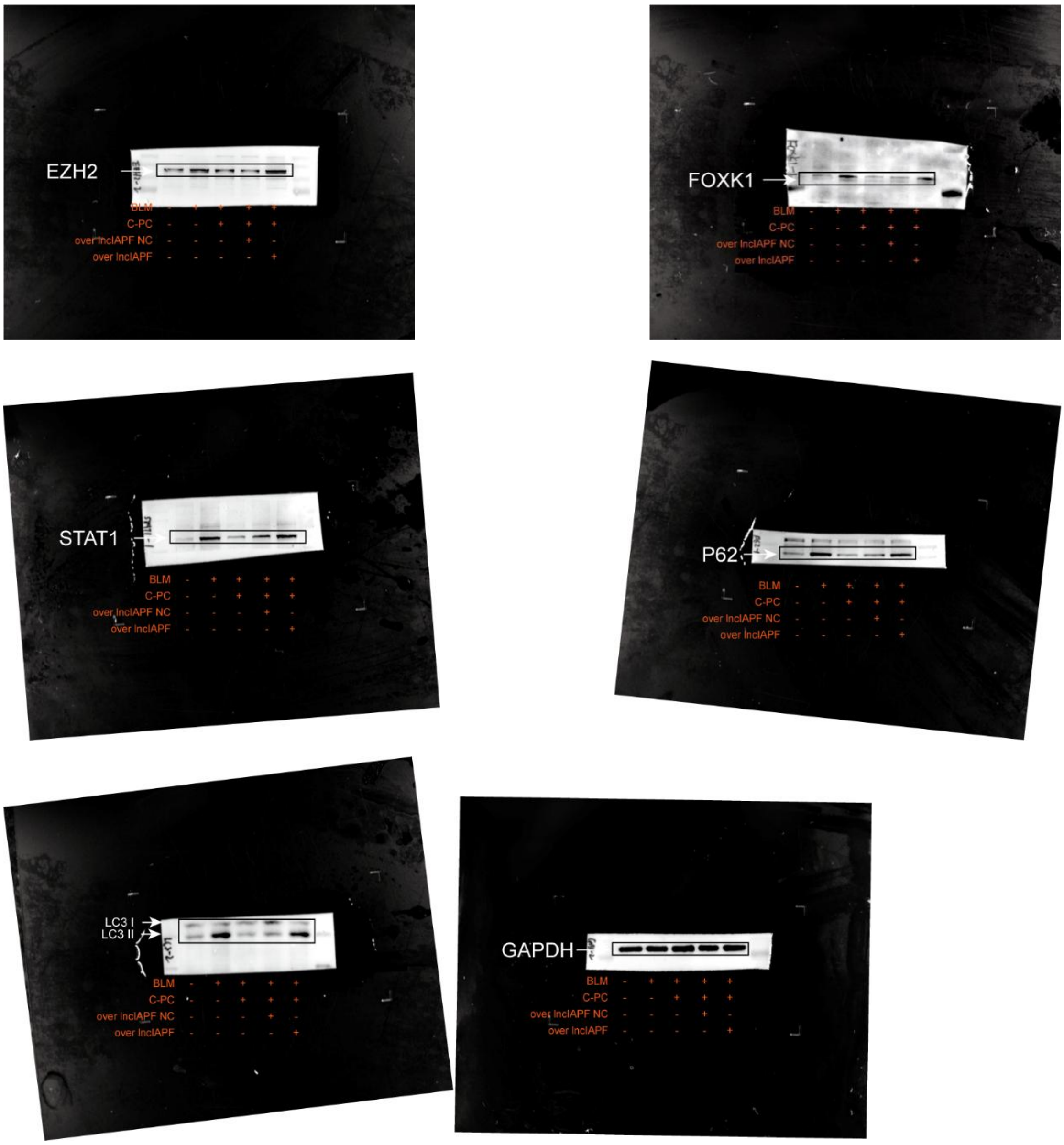
EZH2
BLM
C-PC
over lncIAPF NC
over lncIAPF
FOXK1
BLM
C-PC
over lncIAPF NC
over lncIAPF
STAT1
BLM
C-PC
over lncIAPF NC
over lncIAPF
P62
BLM
C-PC
over lncIAPF NC
over lncIAPF
LC3 I
LC3 II
BLM
C-PC
over lncIAPF NC
over lncIAPF
GAPDH
BLM
C-PC
over lncIAPF NC
over lncIAPF

Figure 6G-3:

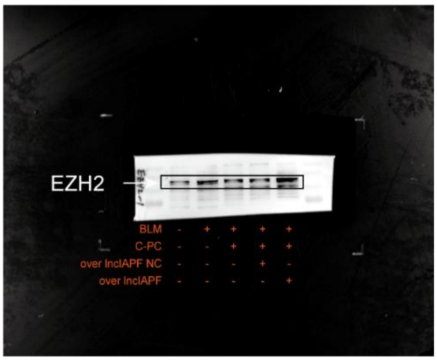
EZH2
BLM
C-PC
over lncIAPF NC
over lncIAPF

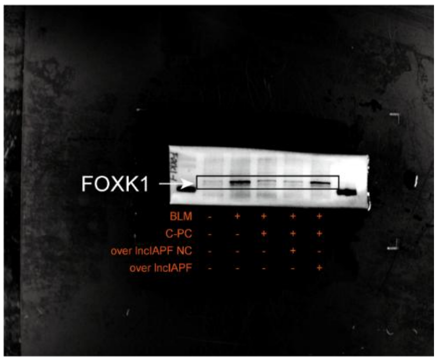
FOXK1
BLM
C-PC
over lncIAPF NC
over lncIAPF

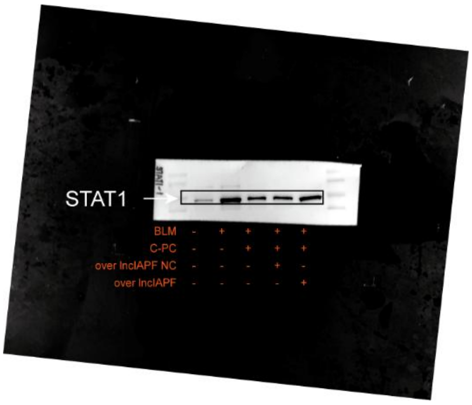
STAT1
BLM
C-PC
over lncIAPF NC
over lncIAPF

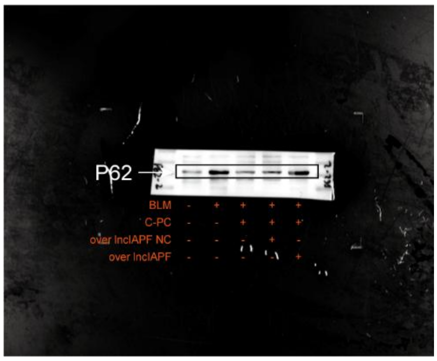
P62
BLM
C-PC
over lncIAPF NC
over lncIAPF

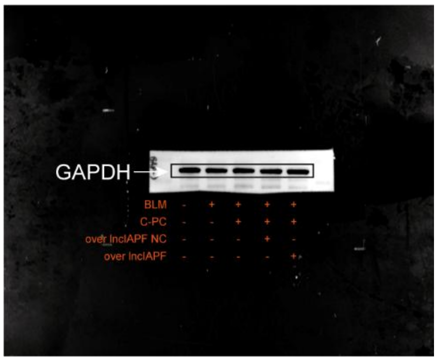
GAPDH
BLM
C-PC
over lncIAPF NC
over lncIAPF

Figure 2F-01:

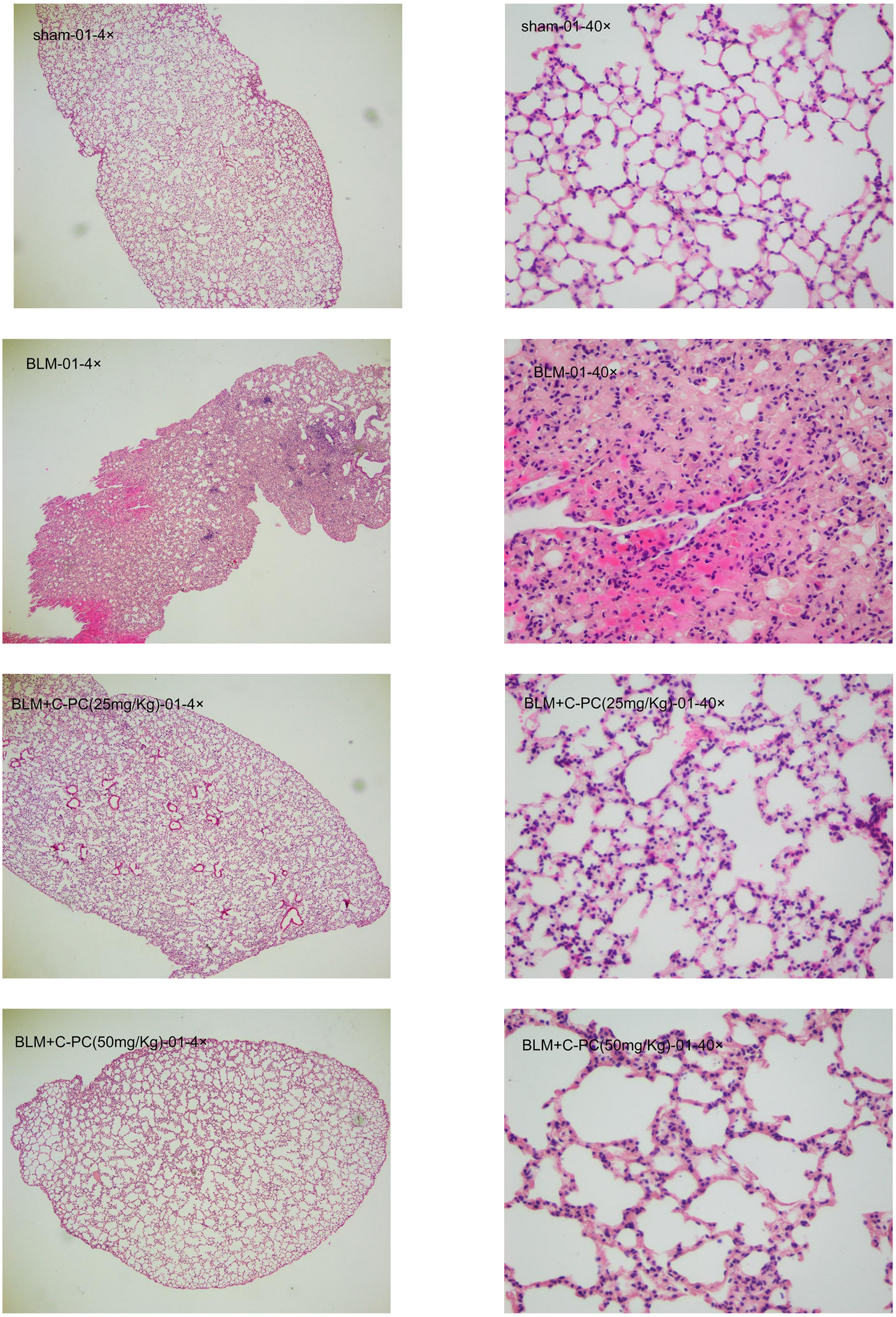
sham-01-4×
sham-01-40×
BLM-01-4×
BLM-01-40×
BLM+C-PC(25mg/Kg)-01-4×
BLM+C-PC(25mg/Kg)-01-40×
BLM+C-PC(50mg/Kg)-01-4×
BLM+C-PC(50mg/Kg)-01-40×

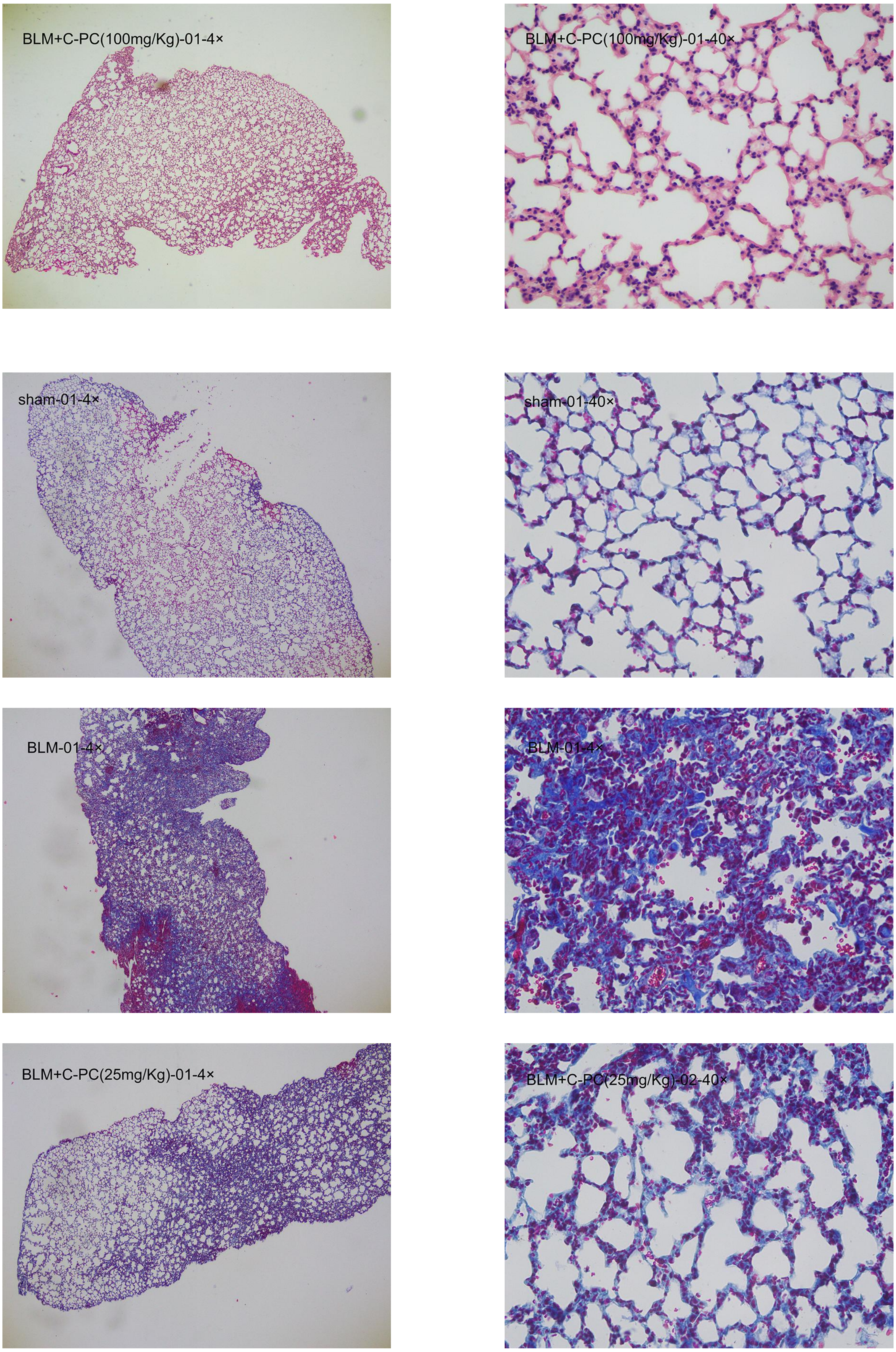
BLM+C-PC(100mg/Kg)-01-4×
BLM+C-PC(100mg/Kg)-01-40×
sham-01-4×
sham-01-40×
BLM-01-4×
BLM-01-4×
BLM+C-PC(25mg/Kg)-01-4×
BLM+C-PC(25mg/Kg)-02-40×

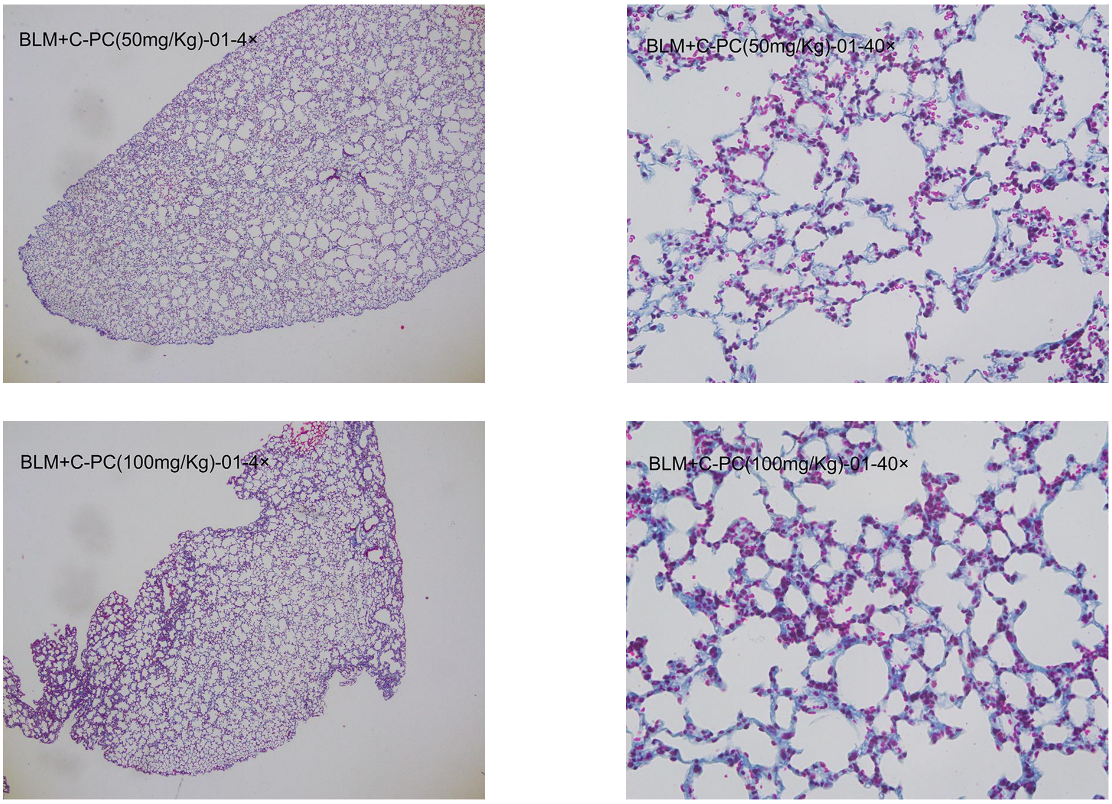
BLM+C-PC(50mg/Kg)-01-4×
BLM+C-PC(50mg/Kg)-01-40×
BLM+C-PC(100mg/Kg)-01-4×
BLM+C-PC(100mg/Kg)-01-40×

Figure 2F-02：

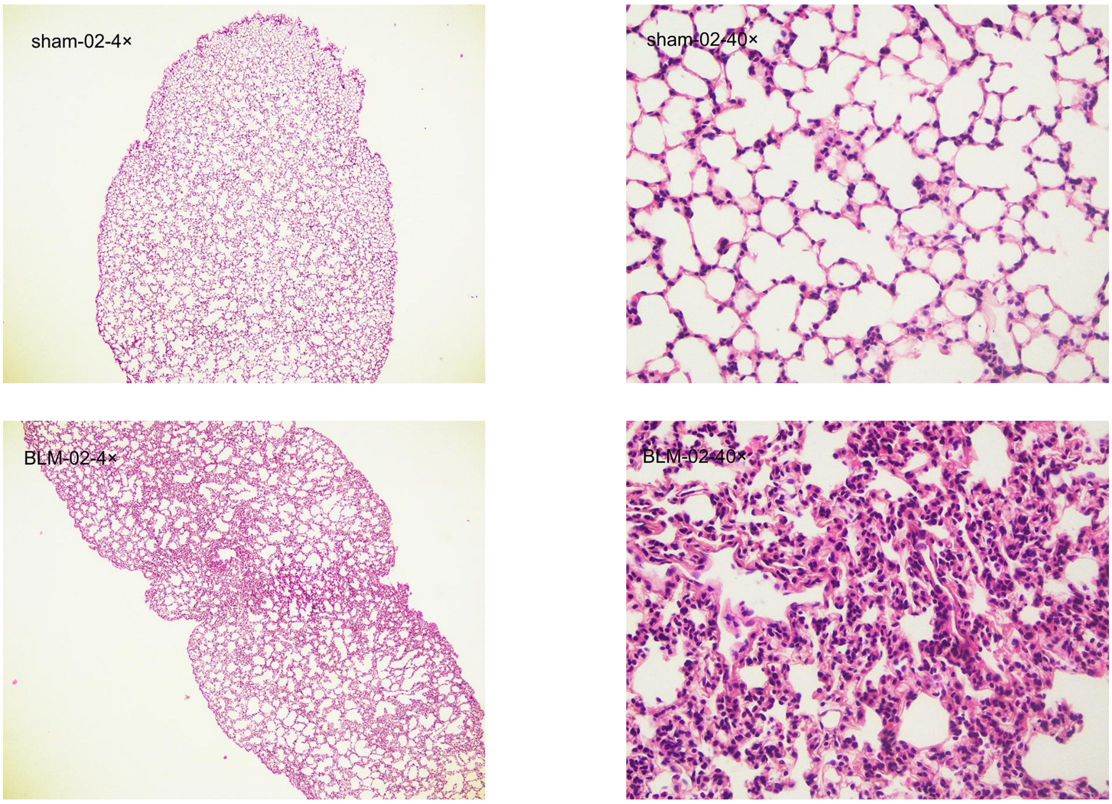
sham-02-4×
sham-02-40×
BLM-02-4×
BLM-02-40×

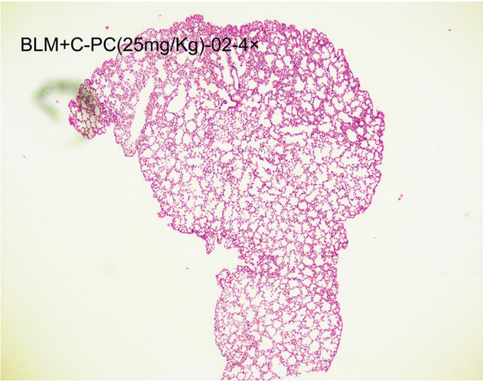
BLM+C-PC(25mg/Kg)-02-4×

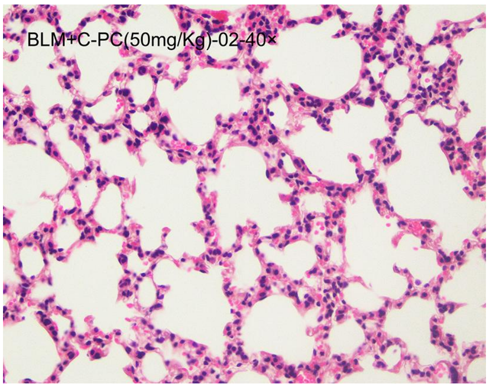
BLM+C-PC(50mg/Kg)-02-40×

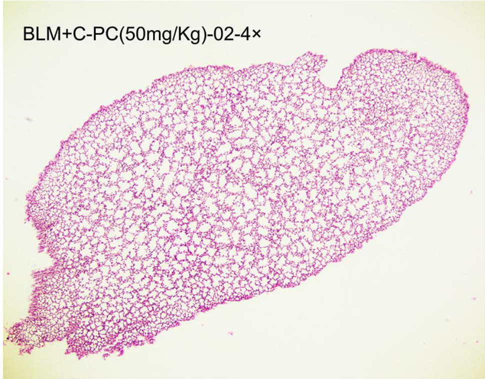
BLM+C-PC(50mg/Kg)-02-4×

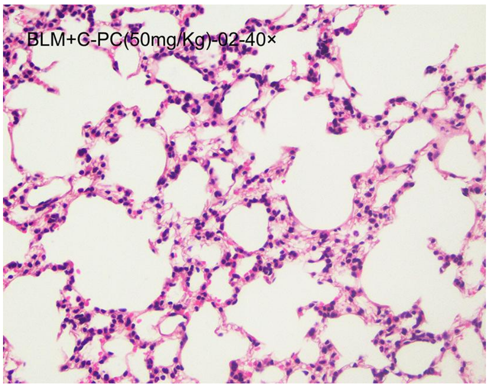
BLM+C-PC(50mg/Kg)-02-40×

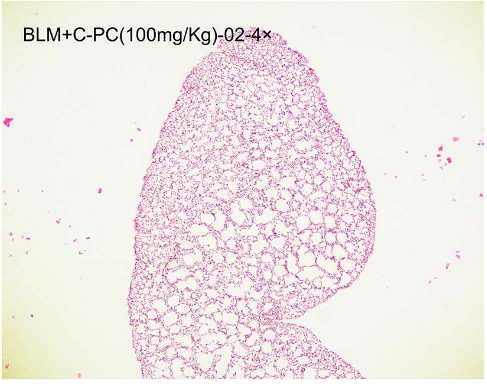
BLM+C-PC(100mg/Kg)-02-4×

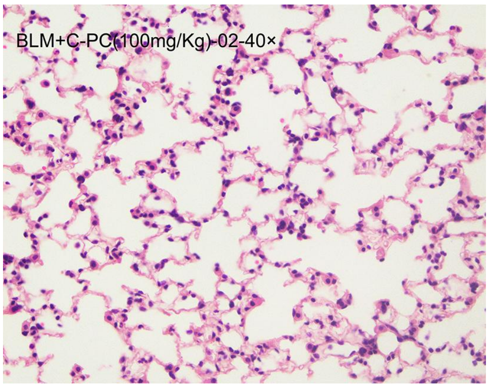
BLM+C-PC(100mg/Kg)-02-40×

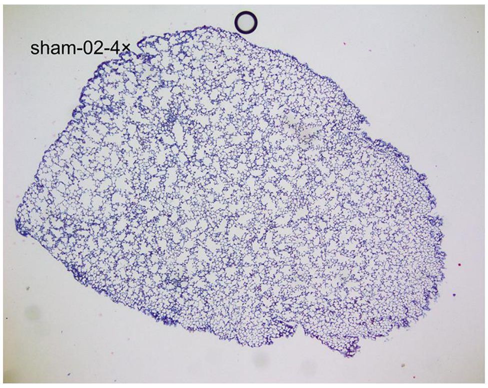
sham-02-4×

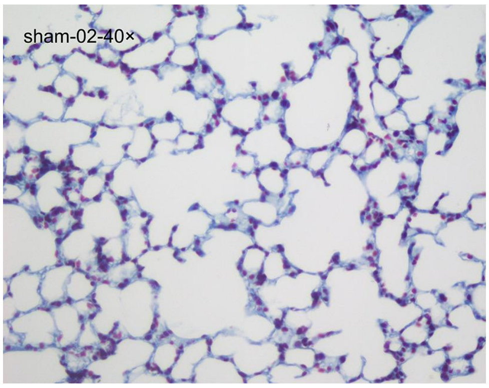
sham-02-40×

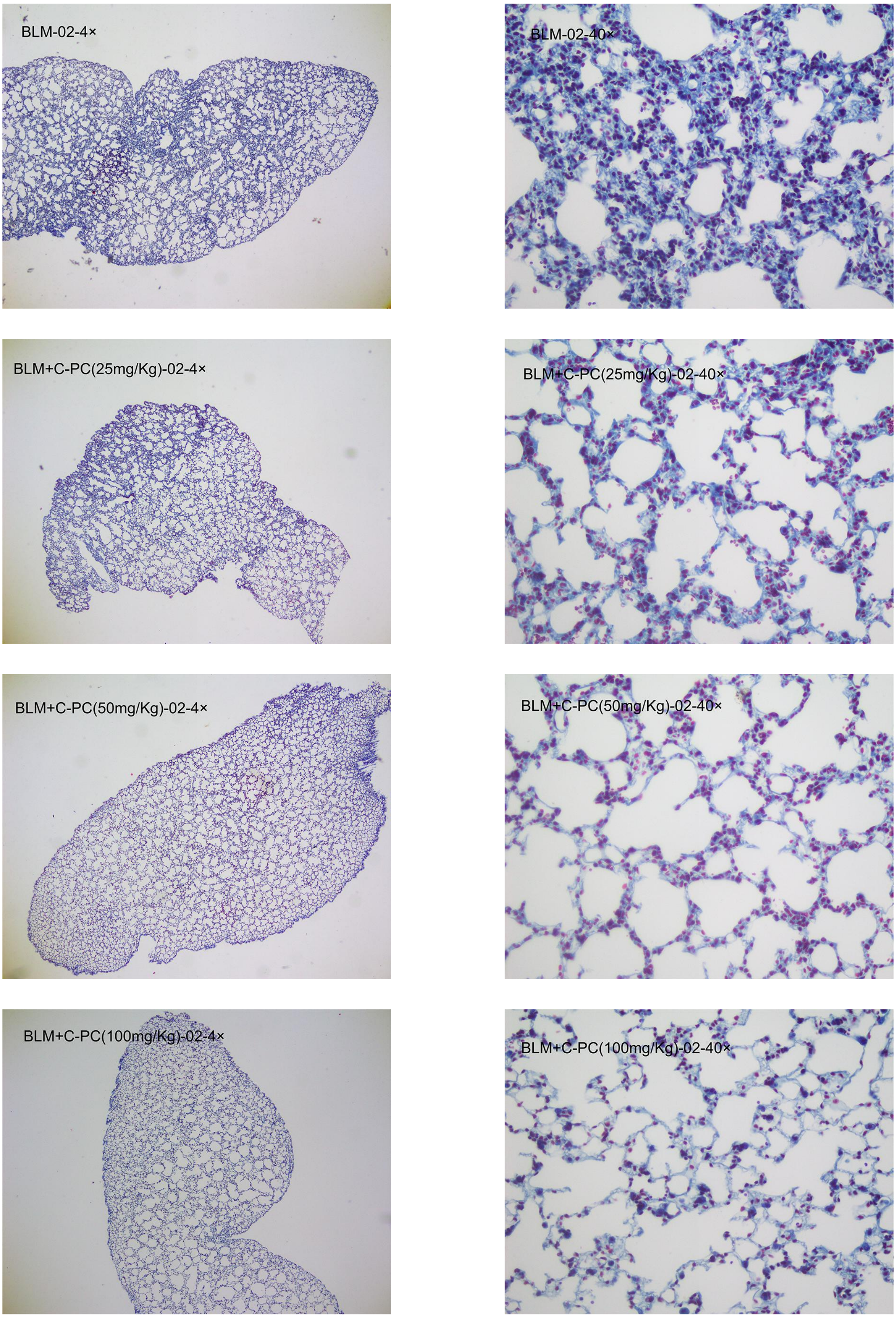
BLM-02-4×
BLM-02-40×
BLM+C-PC(25mg/Kg)-02-4×
BLM+C-PC(25mg/Kg)-02-40×
BLM+C-PC(50mg/Kg)-02-4×
BLM+C-PC(50mg/Kg)-02-40×
BLM+C-PC(100mg/Kg)-02-4×
BLM+C-PC(100mg/Kg)-02-40×

Figure 4G-01

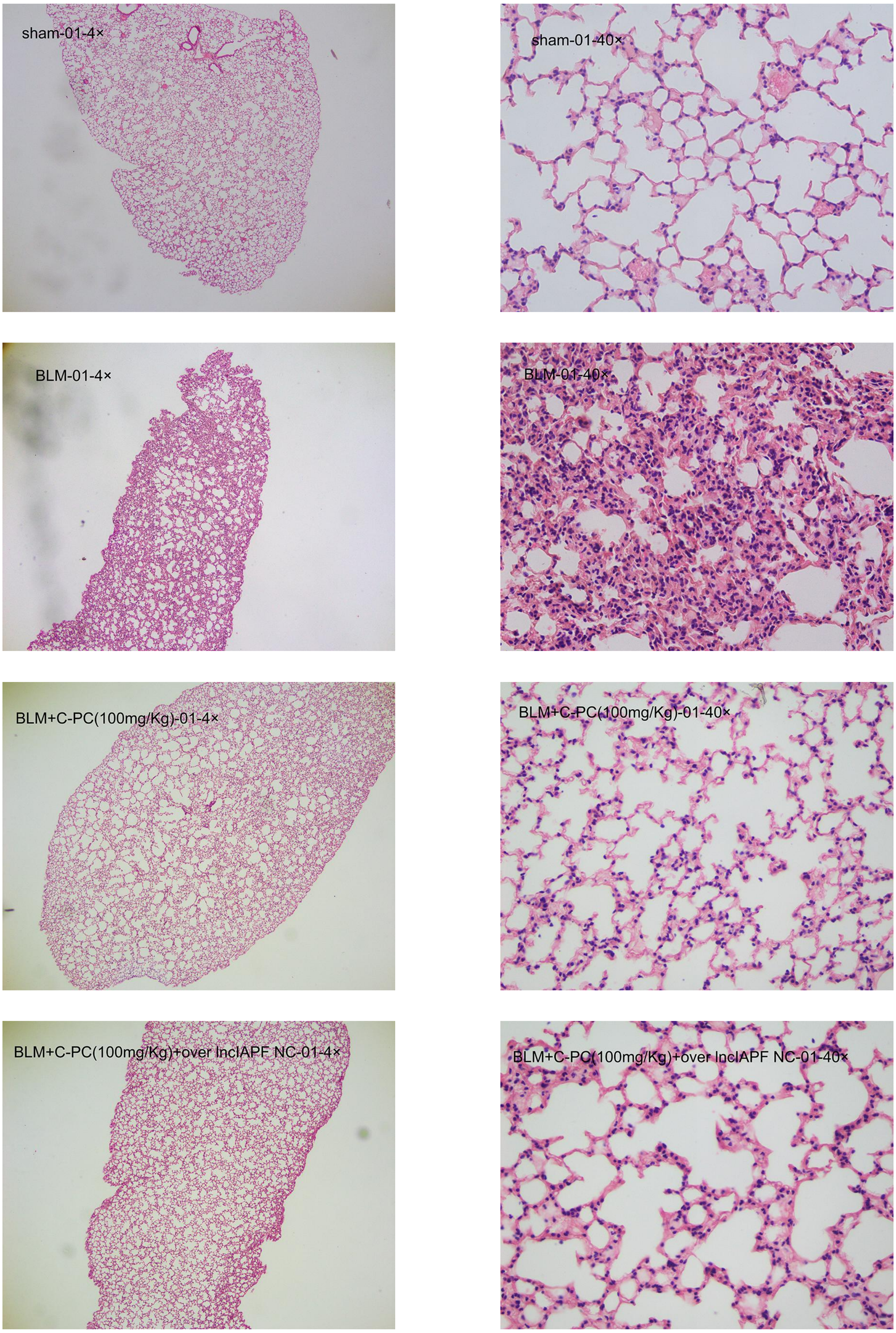
sham-01-4×
sham-01-40×
BLM-01-4×
BLM-01-40×
BLM+C-PC(100mg/Kg)-01-4×
BLM+C-PC(100mg/Kg)-01-40×
BLM+C-PC(100mg/Kg)+over lncIAPF NC-01-4×
BLM+C-PC(100mg/Kg)+over lncIAPF NC-01-40×

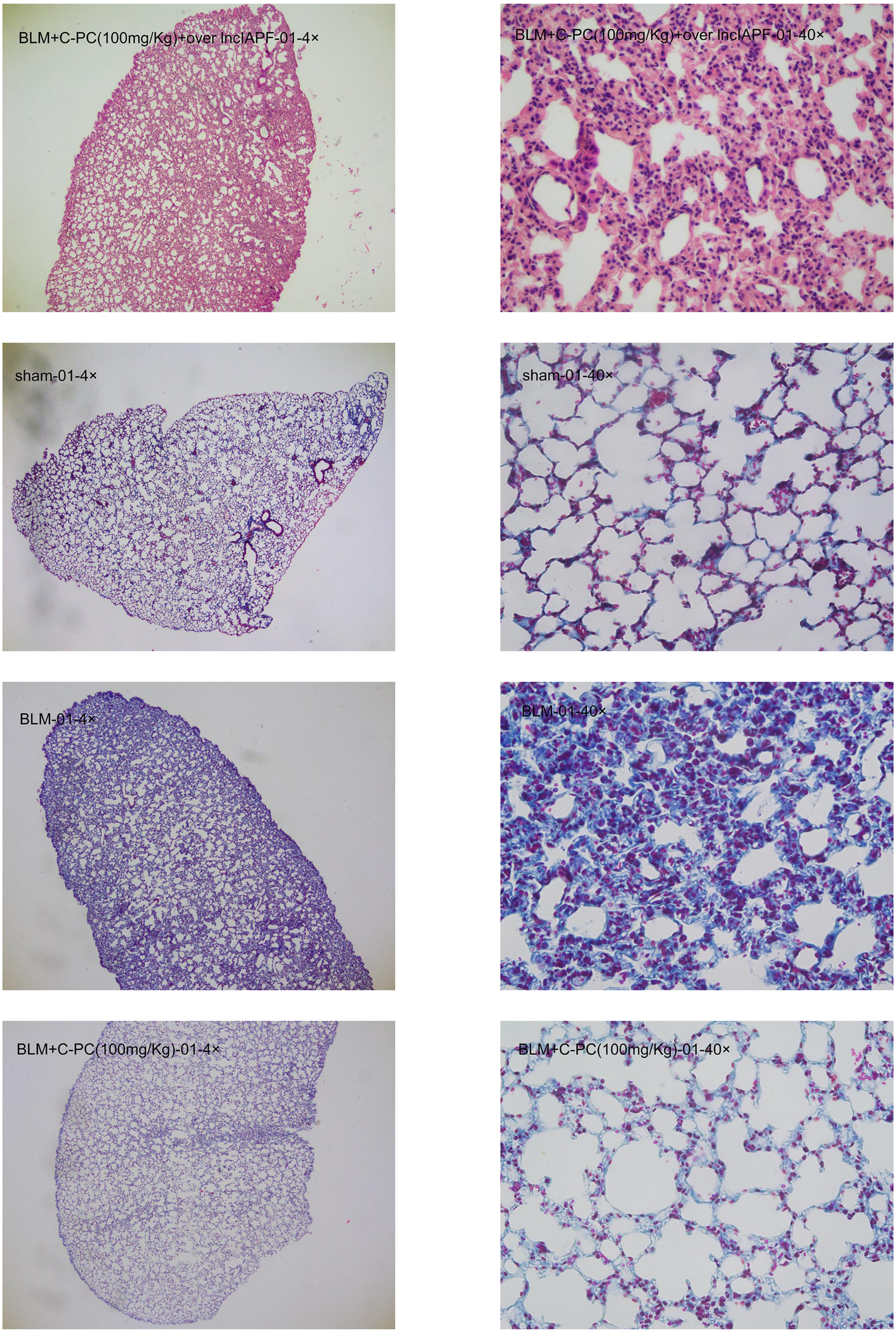
BLM+C-PC(100mg/Kg)+over lncIAPF-01-4×
BLM+C-PC(100mg/Kg)+over lncIAPF-01-40×
sham-01-4×
sham-01-40×
BLM-01-4×
BLM-01-40×
BLM+C-PC(100mg/Kg)-01-4×
BLM+C-PC(100mg/Kg)-01-40×

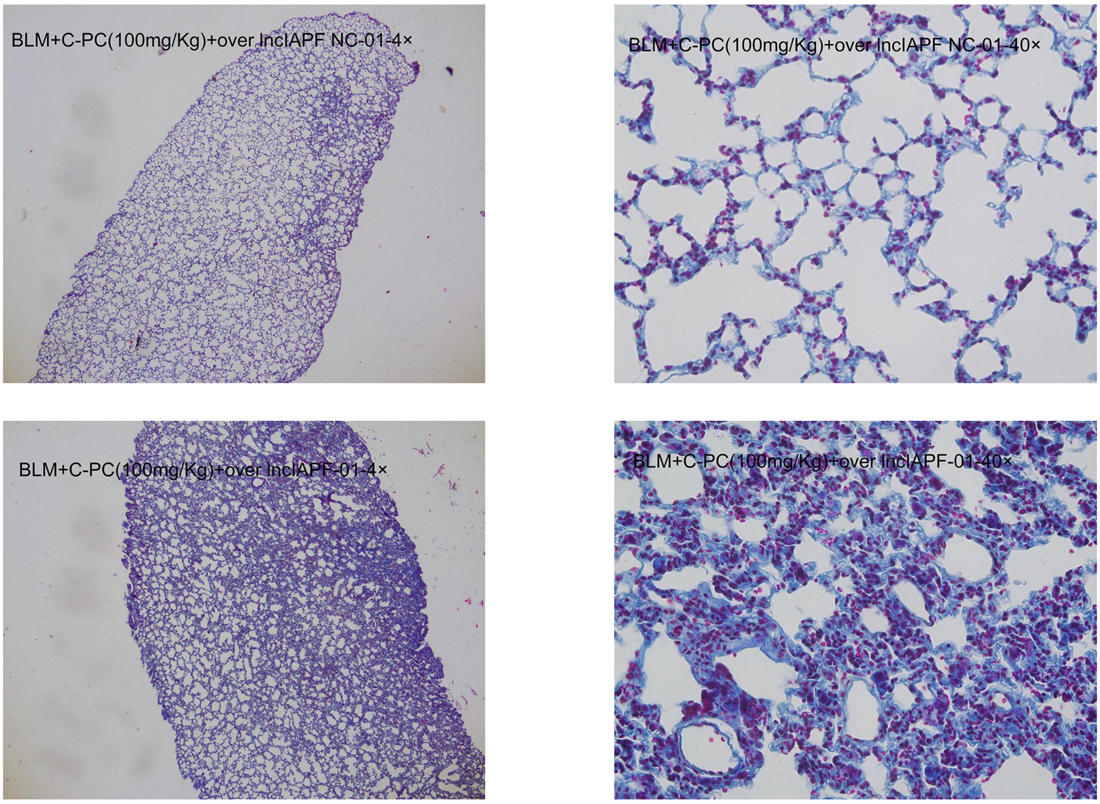
BLM+C-PC(100mg/Kg)+over lnclAPF NC-01-4×
BLM+C-PC(100mg/Kg)+over lnclAPF NC-01-40×
BLM+C-PC(100mg/Kg)+over lnclAPF-01-4×
BLM+C-PC(100mg/Kg)+over lnclAPF-01-40×

Figure 4G-02

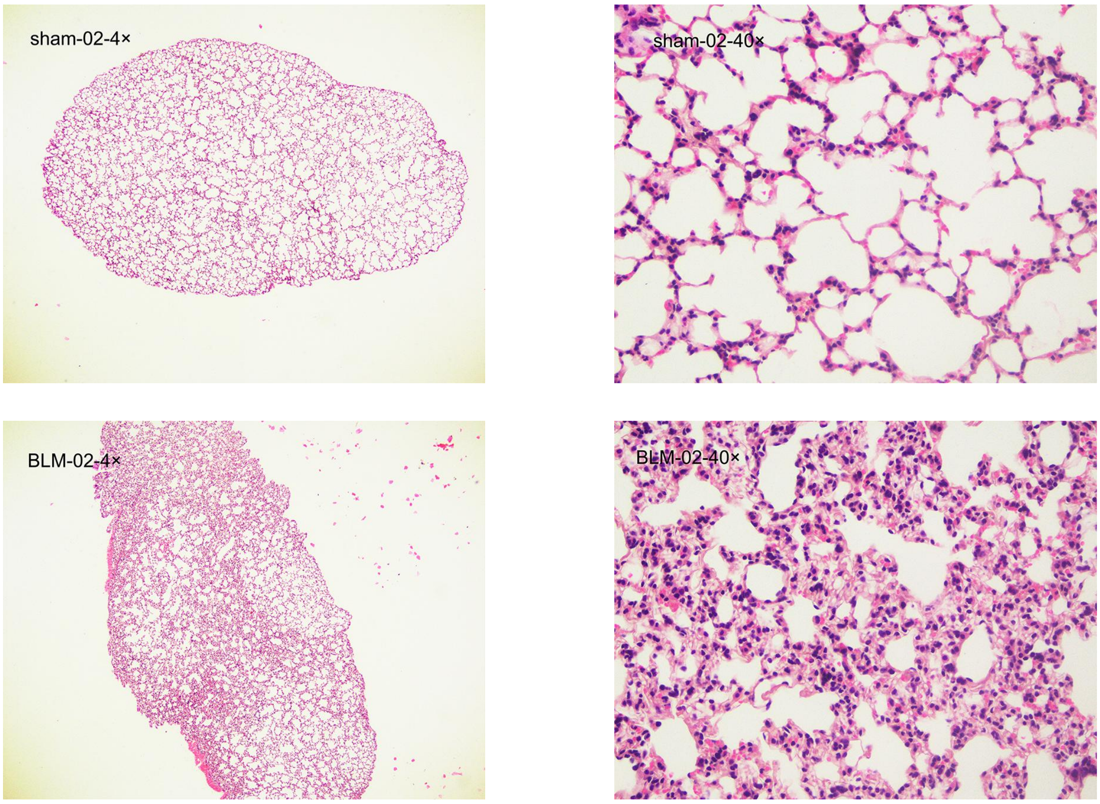
sham-02-4×
sham-02-40×
BLM-02-4×
BLM-02-40×

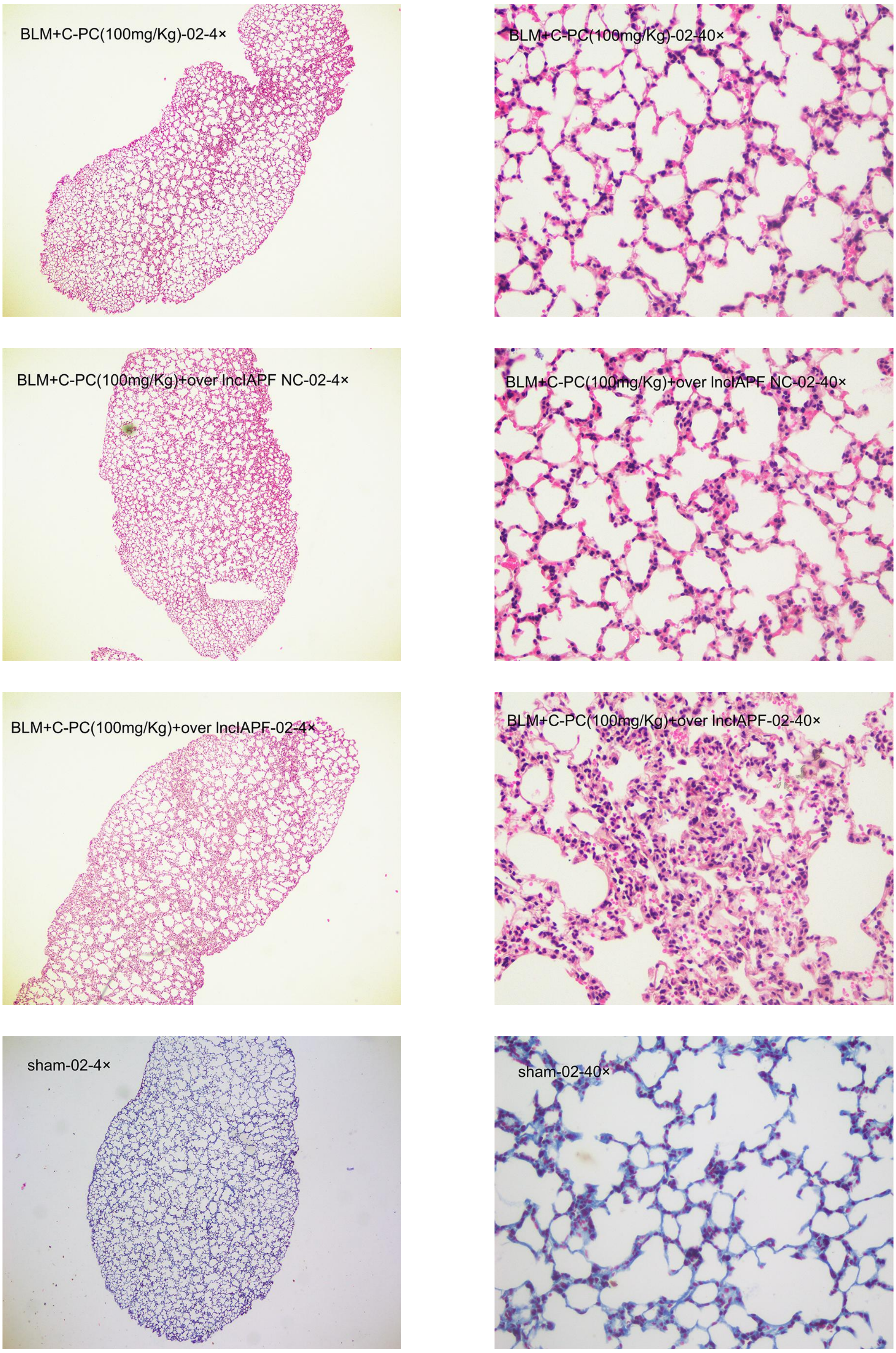
BLM+C-PC(100mg/Kg)-02-4×
BLM+C-PC(100mg/Kg)-02-40×
BLM+C-PC(100mg/Kg)+over lncIAPF NC-02-4×
BLM+C-PC(100mg/Kg)+over lncIAPF NC-02-40×
BLM+C-PC(100mg/Kg)+over lncIAPF-02-4×
BLM+C-PC(100mg/Kg)+over lncIAPF-02-40×
sham-02-4×
sham-02-40×

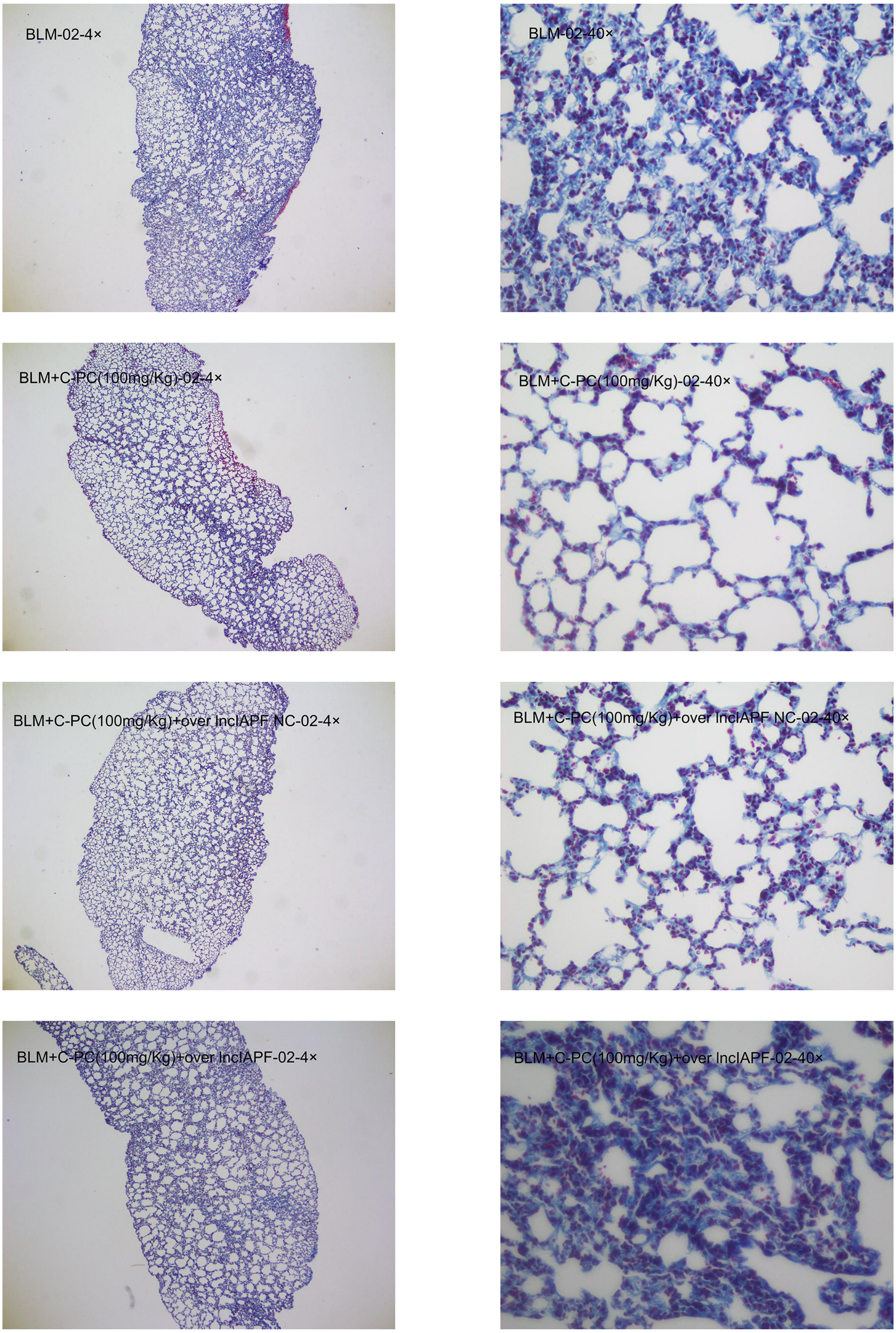
BLM-02-4×
BLM-02-40×
BLM+C-PC(100mg/Kg)-02-4×
BLM+C-PC(100mg/Kg)-02-40×
BLM+C-PC(100mg/Kg)+over lncIAPF NC-02-4×
BLM+C-PC(100mg/Kg)+over lncIAPF NC-02-40×
BLM+C-PC(100mg/Kg)+over lncIAPF-02-4×
BLM+C-PC(100mg/Kg)+over lncIAPF-02-40×

Figure 2E-1

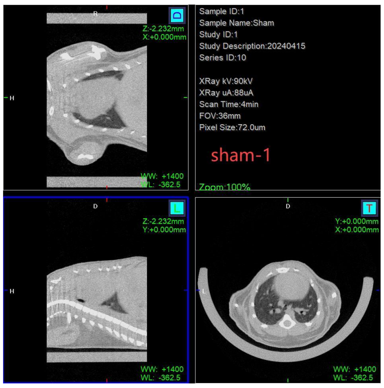
Sample ID:1
Sample Name:Sham
Study ID:1
Study Description:20240415
Series ID:10
XRay kV:90kV
XRay uA:88uA
Scan Time:4min
FOV:36mm
Pixel Size:72.0um
sham-1
Zoom:100%

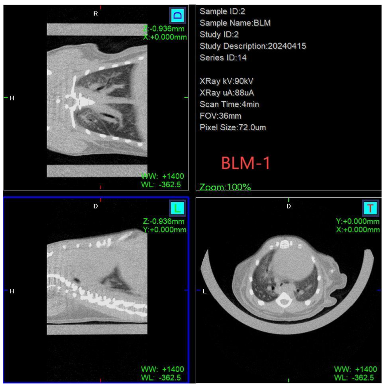
Sample ID:2
Sample Name:BLM
Study ID:2
Study Description:20240415
Series ID:14
XRay kV:90kV
XRay uA:88uA
Scan Time:4min
FOV:36mm
Pixel Size:72.0um
BLM-1
Zoom:100%

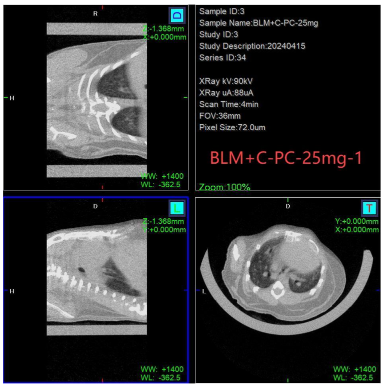
Sample ID:3
Sample Name:BLM+C-PC-25mg
Study ID:3
Study Description:20240415
Series ID:34
XRay kV:90kV
XRay uA:88uA
Scan Time:4min
FOV:36mm
Pixel Size:72.0um
BLM+C-PC-25mg-1
Zoom:100%

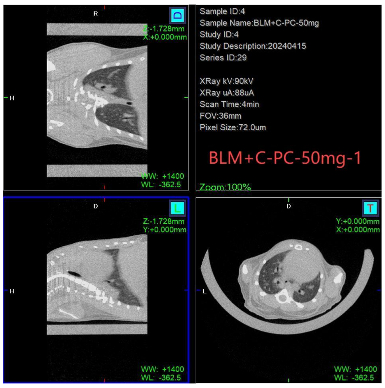
Sample ID:4
Sample Name:BLM+C-PC-50mg
Study ID:4
Study Description:20240415
Series ID:29
XRay kV:90kV
XRay uA:88uA
Scan Time:4min
FOV:36mm
Pixel Size:72.0um
BLM+C-PC-50mg-1
Zoom:100%

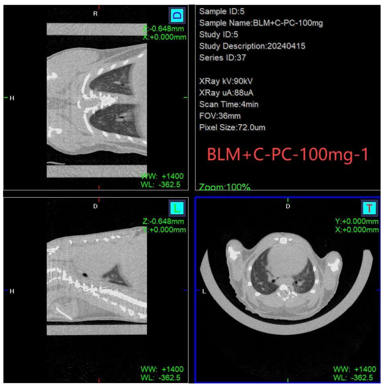
Sample ID:5
Sample Name:BLM+C-PC-100mg
Study ID:5
Study Description:20240415
Series ID:37
XRay kV:90kV
XRay uA:88uA
Scan Time:4min
FOV:36mm
Pixel Size:72.0um
BLM+C-PC-100mg-1
Zoom:100%

Figure 2E-2

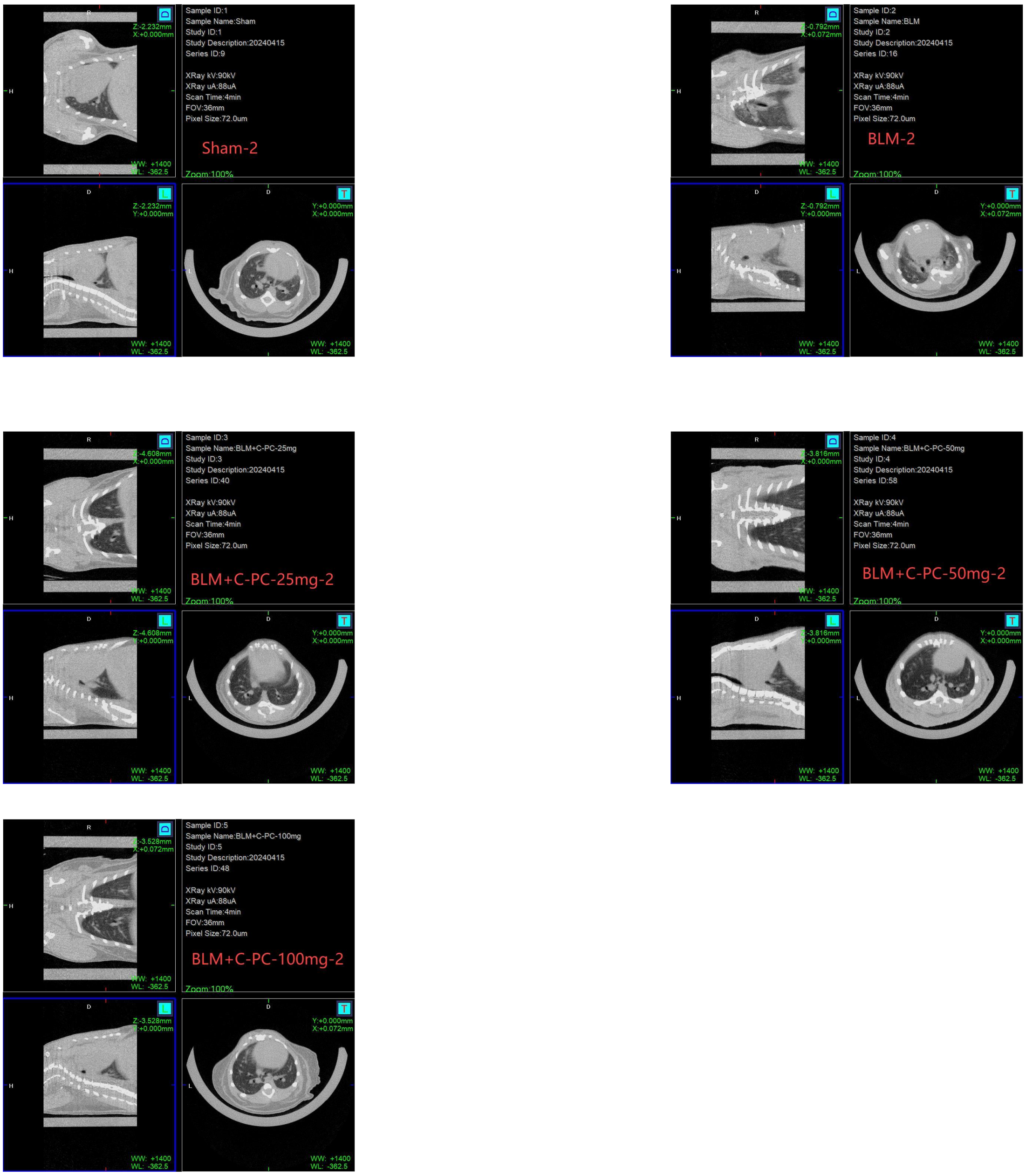
Sample ID:1
Sample Name:Sham
Study ID:1
Study Description:20240415
Series ID:9
XRay kV:90kV
XRay uA:88uA
Scan Time:4min
FOV:36mm
Pixel Size:72.0um
Sham-2
Sample ID:2
Sample Name:BLM
Study ID:2
Study Description:20240415
Series ID:16
XRay kV:90kV
XRay uA:88uA
Scan Time:4min
FOV:36mm
Pixel Size:72.0um
BLM-2
Sample ID:3
Sample Name:BLM+C-PC-25mg
Study ID:3
Study Description:20240415
Series ID:40
XRay kV:90kV
XRay uA:88uA
Scan Time:4min
FOV:36mm
Pixel Size:72.0um
BLM+C-PC-25mg-2
Sample ID:4
Sample Name:BLM+C-PC-50mg
Study ID:4
Study Description:20240415
Series ID:58
XRay kV:90kV
XRay uA:88uA
Scan Time:4min
FOV:36mm
Pixel Size:72.0um
BLM+C-PC-50mg-2
Sample ID:5
Sample Name:BLM+C-PC-100mg
Study ID:5
Study Description:20240415
Series ID:48
XRay kV:90kV
XRay uA:88uA
Scan Time:4min
FOV:36mm
Pixel Size:72.0um
BLM+C-PC-100mg-2

Figure 2E-3

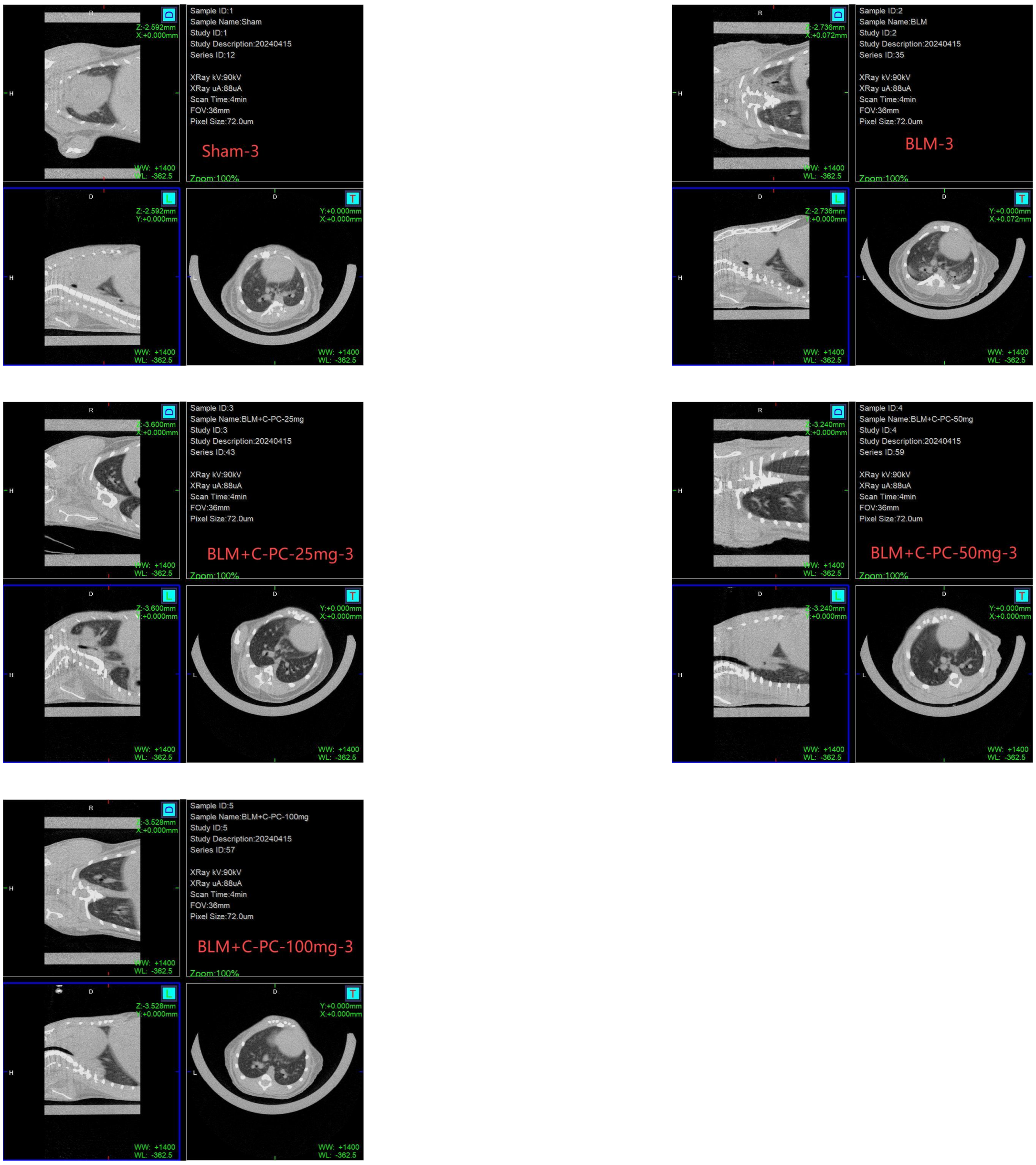
Sample ID:1
Sample Name:Sham
Study ID:1
Study Description:20240415
Series ID:12
XRay kV:90kV
XRay uA:88uA
Scan Time:4min
FOV:36mm
Pixel Size:72.0um
Sham-3
Sample ID:2
Sample Name:BLM
Study ID:2
Study Description:20240415
Series ID:35
XRay kV:90kV
XRay uA:88uA
Scan Time:4min
FOV:36mm
Pixel Size:72.0um
BLM-3
Sample ID:3
Sample Name:BLM+C-PC-25mg
Study ID:3
Study Description:20240415
Series ID:43
XRay kV:90kV
XRay uA:88uA
Scan Time:4min
FOV:36mm
Pixel Size:72.0um
BLM+C-PC-25mg-3
Sample ID:4
Sample Name:BLM+C-PC-50mg
Study ID:4
Study Description:20240415
Series ID:59
XRay kV:90kV
XRay uA:88uA
Scan Time:4min
FOV:36mm
Pixel Size:72.0um
BLM+C-PC-50mg-3
Sample ID:5
Sample Name:BLM+C-PC-100mg
Study ID:5
Study Description:20240415
Series ID:57
XRay kV:90kV
XRay uA:88uA
Scan Time:4min
FOV:36mm
Pixel Size:72.0um
BLM+C-PC-100mg-3

Figure 4F-1

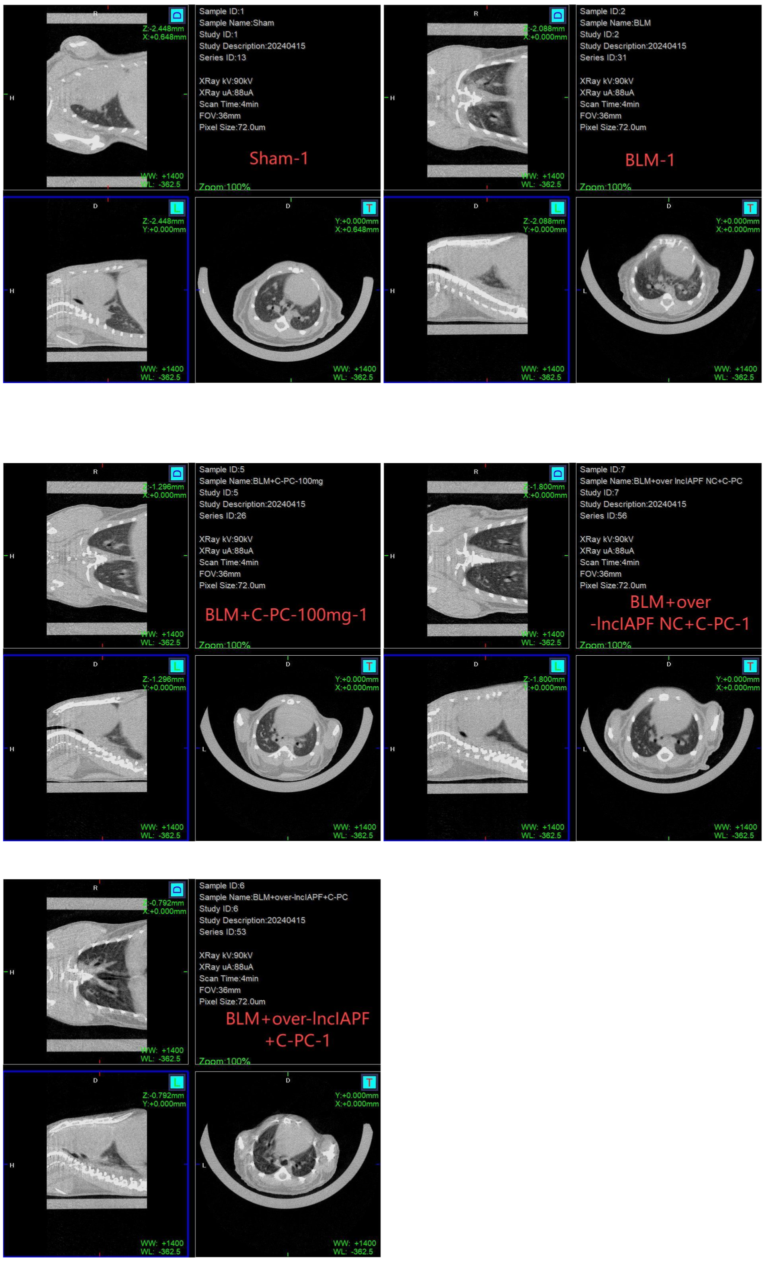
Sample ID:1
Sample Name:Sham
Study ID:1
Study Description:20240415
Series ID:13
XRay kV:90kV
XRay uA:88uA
Scan Time:4min
FOV:36mm
Pixel Size:72.0um
Sham-1
Sample ID:2
Sample Name:BLM
Study ID:2
Study Description:20240415
Series ID:31
XRay kV:90kV
XRay uA:88uA
Scan Time:4min
FOV:36mm
Pixel Size:72.0um
BLM-1
Sample ID:5
Sample Name:BLM+C-PC-100mg
Study ID:5
Study Description:20240415
Series ID:26
XRay kV:90kV
XRay uA:88uA
Scan Time:4min
FOV:36mm
Pixel Size:72.0um
BLM+C-PC-100mg-1
Sample ID:7
Sample Name:BLM+over lncIAPF NC+C-PC
Study ID:7
Study Description:20240415
Series ID:56
XRay kV:90kV
XRay uA:88uA
Scan Time:4min
FOV:36mm
Pixel Size:72.0um
BLM+over
-lncIAPF NC+C-PC-1
Sample ID:6
Sample Name:BLM+over-lncIAPF+C-PC
Study ID:6
Study Description:20240415
Series ID:53
XRay kV:90kV
XRay uA:88uA
Scan Time:4min
FOV:36mm
Pixel Size:72.0um
BLM+over-lncIAPF
+C-PC-1

Figure 4F-2

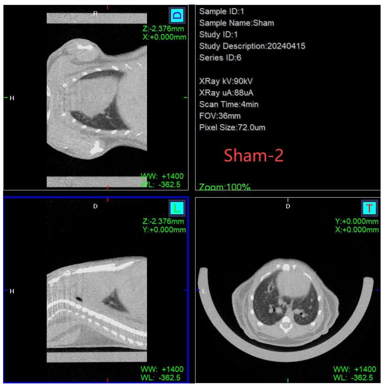
Sample ID:1
Sample Name:Sham
Study ID:1
Study Description:20240415
Series ID:6
XRay kV:90kV
XRay uA:88uA
Scan Time:4min
FOV:36mm
Pixel Size:72.0um
Sham-2

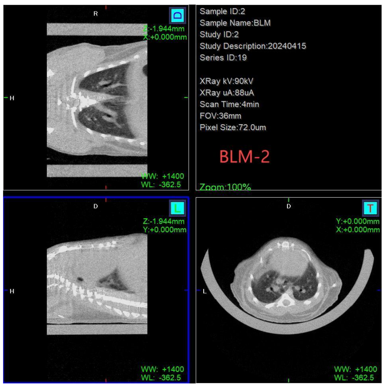
Sample ID:2
Sample Name:BLM
Study ID:2
Study Description:20240415
Series ID:19
XRay kV:90kV
XRay uA:88uA
Scan Time:4min
FOV:36mm
Pixel Size:72.0um
BLM-2

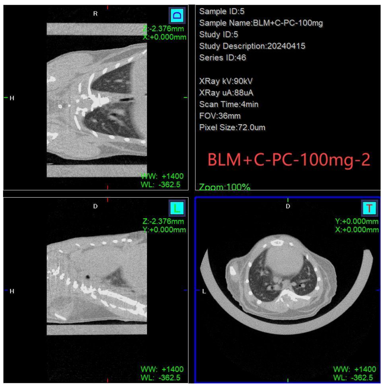
Sample ID:5
Sample Name:BLM+C-PC-100mg
Study ID:5
Study Description:20240415
Series ID:46
XRay kV:90kV
XRay uA:88uA
Scan Time:4min
FOV:36mm
Pixel Size:72.0um
BLM+C-PC-100mg-2

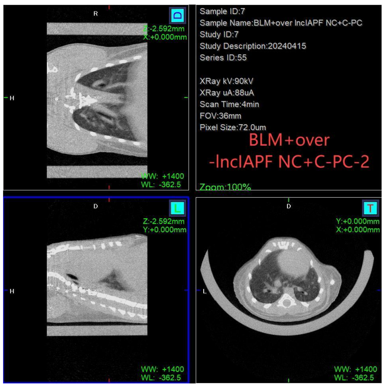
Sample ID:7
Sample Name:BLM+over lncIAPF NC+C-PC
Study ID:7
Study Description:20240415
Series ID:55
XRay kV:90kV
XRay uA:88uA
Scan Time:4min
FOV:36mm
Pixel Size:72.0um
BLM+over
-lncIAPF NC+C-PC-2

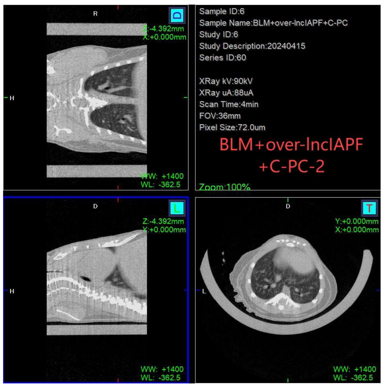
Sample ID:6
Sample Name:BLM+over-lncIAPF+C-PC
Study ID:6
Study Description:20240415
Series ID:60
XRay kV:90kV
XRay uA:88uA
Scan Time:4min
FOV:36mm
Pixel Size:72.0um
BLM+over-lncIAPF
+C-PC-2

Figure 4F-3

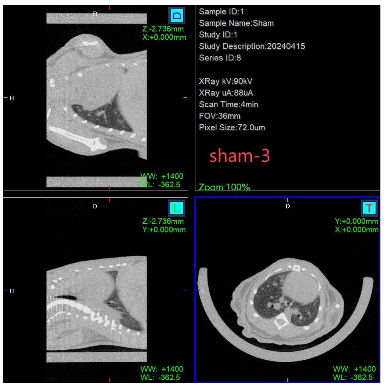
Sample ID:1
Sample Name:Sham
Study ID:1
Study Description:20240415
Series ID:8
XRay kV:90kV
XRay uA:88uA
Scan Time:4min
FOV:36mm
Pixel Size:72.0um
sham-3
Zoom:100%
WW: +1400
WL: -362.5

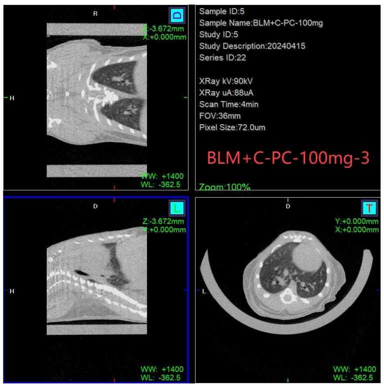
Sample ID:5
Sample Name:BLM+C-PC-100mg
Study ID:5
Study Description:20240415
Series ID:22
XRay kV:90kV
XRay uA:88uA
Scan Time:4min
FOV:36mm
Pixel Size:72.0um
BLM+C-PC-100mg-3
Zoom:100%
WW: +1400
WL: -362.5

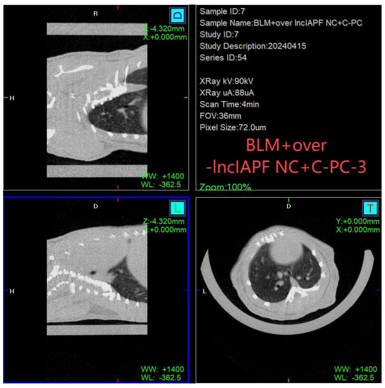
Sample ID:7
Sample Name:BLM+over lncIAPF NC+C-PC
Study ID:7
Study Description:20240415
Series ID:54
XRay kV:90kV
XRay uA:88uA
Scan Time:4min
FOV:36mm
Pixel Size:72.0um
BLM+over
-lncIAPF NC+C-PC-3
Zoom:100%
WW: +1400
WL: -362.5

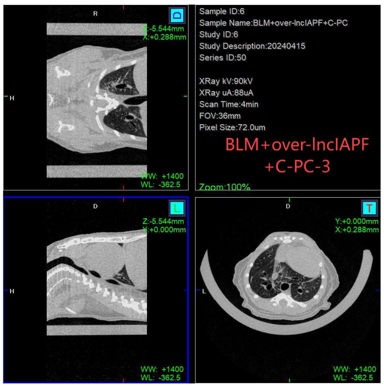
Sample ID:6
Sample Name:BLM+over-lncIAPF+C-PC
Study ID:6
Study Description:20240415
Series ID:50
XRay kV:90kV
XRay uA:88uA
Scan Time:4min
FOV:36mm
Pixel Size:72.0um
BLM+over-lncIAPF
+C-PC-3
Zoom:100%
WW: +1400
WL: -362.5
