# Supplementary material for: C-phycocyanin reinforces autophagy to block pulmonary fibrogenesis by inhibiting lncIAPF biogenesis
Source: Arch Pharm Res. 2024 Jul 22;47(7):659–74. doi: 10.1007/s12272-024-01508-y (PMC11300487; doi:10.1007/s12272-024-01508-y)
Supplement: Supplementary file 2 — Supplementary file2 (PDF 940 kb) [file 12272_2024_1508_MOESM2_ESM.pdf]

ATF3 plasmid construction information:

The ATF3 plasmid vector was purchased from HanBio Biotechnology Company (Shanghai, China).

The map of pcDNA3.1 vector is shown in the following figure:

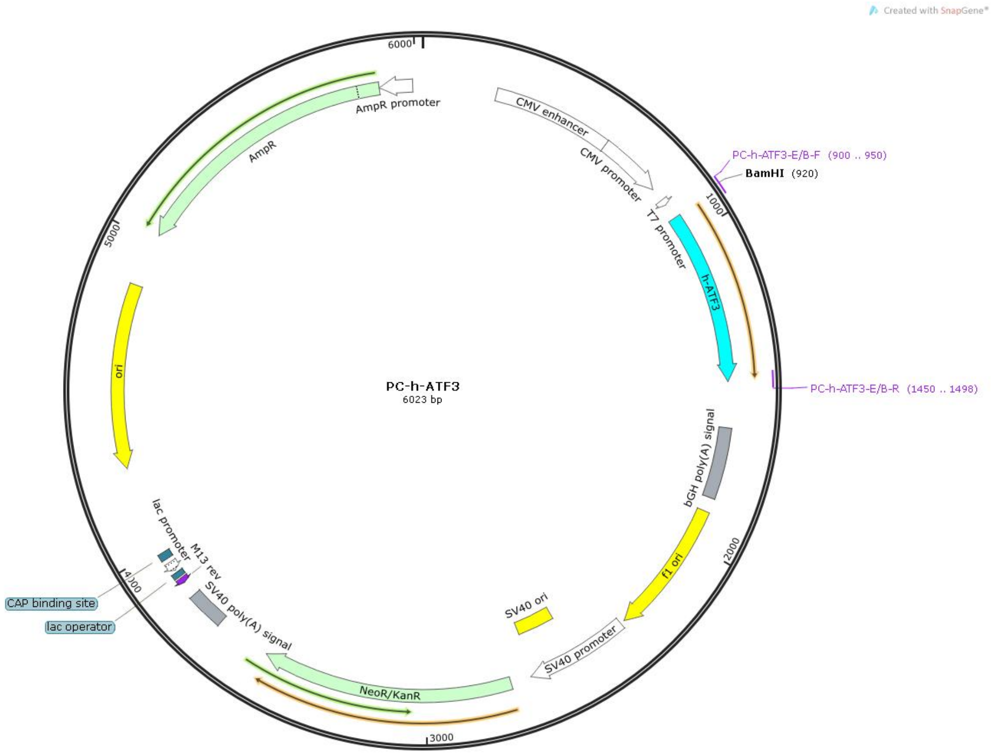

Gene sequence information:

Atgatgcttcaacacccaggccaggtctctgcctcggaagtgagtgcttctgccatcgtcccctgcctgtcccctcctgggt

cactggtgtttgaggattttgctaacctgacgccctttgtcaaggaagagctgaggtttgccatccagaacaagcacctctgc

caccggatgtcctctgcgctggaatcagtcactgtcagcgacagacccctcggggtgtccatcacaaaagccgaggtagc

ccctgaagaagatgaaaggaaaaagaggcgacgagaaagaaataagattgcagctgcaaagtgccgaaacaagaaga

aggagaagacggagtgcctgcagaaagagtcggagaagctggaaagtgtgaatgctgaactgaaggctcagattgagg

agctcaagaacgagaagcagcatttgatatacatgctcaaccttcatcggcccacgtgtattgtccgggctcagaatgggag

gactccagaagatgagagaaacctctttatccaacagataaaagaaggaacattgcagagctaa

Primer design:

PC-h-ATF3-E/B-F	TTAAGCTTGGTACCGAGCTCGGATCCGCCACCatgatgcttcaacacccag
PC-h-ATF3-E/B-R	CCACTGTGCTGGATATCTGCAttagctctgcaatgttccttcttttatc

The results of sequencing comparison are shown in the following figure: (the green area is the part that matches the target sequence)

```
NoName          1 mmlqhpgqvsasevsasaivpclsppgslvfedfanltpfvkeelrfaiqnkhlchrmssalesvtvsdrplgvsitkaevapeederkkrrrernkiaaakcrnkkkekteclqkeseklesvnaelkaqieelknekqhliymlnlhrptcivraqngrtpedernlfiqqikegtlqs*crypaqwrplehas-------------
08-22PC-h-AT  120 mmlqhpgqvsasevsasaivpclsppgslvfedfanltpfvkeelrfaiqnkhlchrmssalesvtvsdrplgvsitkaevapeederkkrrrernkiaaakcrnkkkekteclqkeseklesvnaelkaqieelknekqhliymlnlhrptcivraqngrtpedernlfiqqikegtlqs*crypaqwrplehasrgpfeqkliseedl
```

The sequencing results showed that the sequencing results were consistent with the target sequence, and the target plasmid was successfully constructed.

Lnc00941（lncIAPF） plasmid construction information:

The lncIAPF plasmid vector was purchased from Heyuan Biotechnology Company (Shanghai, China).

Clone serial number：H12557；Gene size：1967

The map of pcDNA3.1 vector is shown in the following figure:

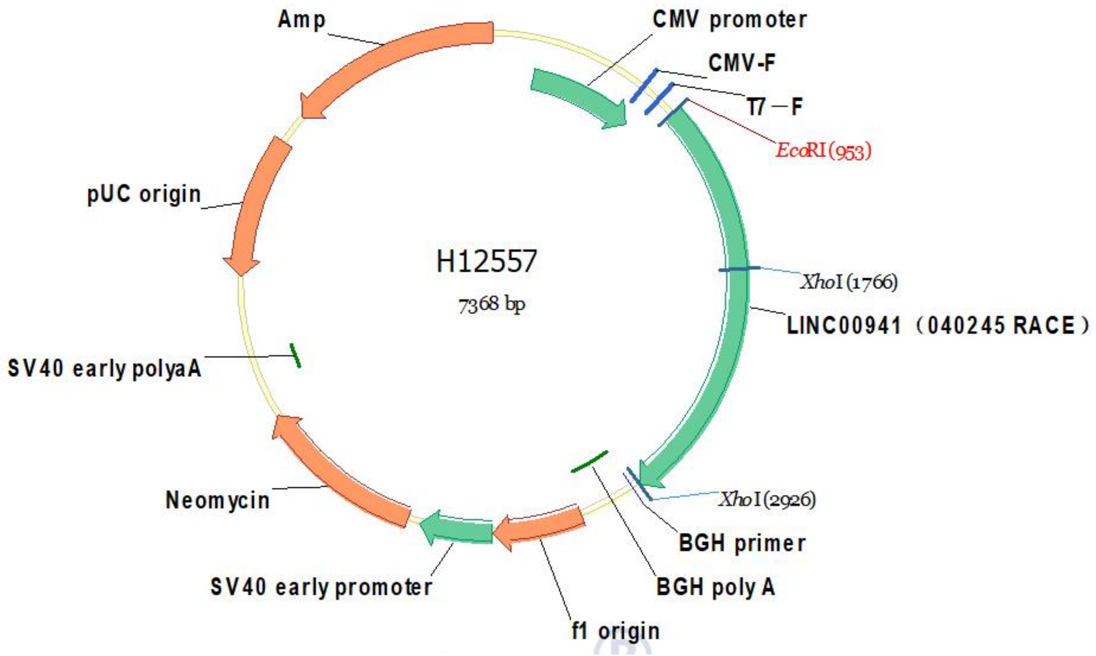

Primer design:

Forward sequencing primer	CMV-F CGCAAATGGGCGGTAGGCGTG
Reverse sequencing primer	BGH TAGAAGGCACAGTCGAGG

Comparison of sequencing results:

1 50

seq (1) CTCTCTGGCTAACTAGAGAACCCACTGCTTACTGGCTTATCGAAATTAAT

LINC00941（040245 RACE） (1) --

51 100

seq (51) ACGACTCACTATAGGGAGACCCAAGCTGGCTAGCGTTTAAACTTAAGCTT

LINC00941（040245 RACE） (1) --

101 150

seq (101) GGTACCGAGCTCGGATCCACTAGTCCAGTGTGGTGGAATTCTTGCCCATG

LINC00941（040245 RACE） (1) ---TTGCCCATG

151 200

seq (151) GAGATGAGTTTTATGTGAGCCAGGTTTACAAAGAGTAGAGTGTGGCTGGG

LINC00941（040245 RACE） (10) GAGATGAGTTTTATGTGAGCCAGGTTTACAAAGAGTAGAGTGTGGCTGGG

201 250

seq (201) CACGGTGGCTCACGCCTGTAATCCCAGCACTTTGGGGGACGTGGCTGAAG

LINC00941（040245 RACE） (60) CACGGTGGCTCACGCCTGTAATCCCAGCACTTTGGGGGACGTGGCTGAAG

251 300

seq (251) TCAGTCGGGCCGCCCGCTTGCTGCACGGCAGCCGTGGGACTGGCACAGGC

LINC00941（040245 RACE） (110) TCAGTCGGGCCGCCCGCTTGCTGCACGGCAGCCGTGGGACTGGCACAGGC

301 350

seq (301) TCCTCGGCAGCATTATGGGCAGCTGGTAGAGCGGCCACTTCTGAGAGCCG

LINC00941（040245 RACE） (160) TCCTCGGCAGCATTATGGGCAGCTGGTAGAGCGGCCACTTCTGAGAGCCG

351 400

seq (351) GATGGGCCTTCCGAGGTCGGGCCGTGCCCCGCTGGGCAGCGCAGAGGGGG

LINC00941（040245 RACE） (210) GATGGGCCTTCCGAGGTCGGGCCGTGCCCCGCTGGGCAGCGCAGAGGGGG

401 450

seq (401) TGTGTAGGGCACGCGACTTGGAGCCAGGTCCAGACCCATCCTGGCTCCTC

LINC00941（040245 RACE） (260) TGTGTAGGGCACGCGACTTGGAGCCAGGTCCAGACCCATCCTGGCTCCTC

451 500

seq (451) TCAGGGCTCCTTCCCCTGCCCCCATTCCACCCCAGCCCTGCTGGCTGTCC

LINC00941（040245 RACE） (310) TCAGGGCTCCTTCCCCTGCCCCCATTCCACCCCAGCCCTGCTGGCTGTCC

501 550

seq (501) CGCAGGACCCAGCGCCGCGGTAGCCTTCTCTGAACTGCGGCTCAGGCGGA

LINC00941（040245 RACE） (360) CGCAGGACCCAGCGCCGCGGTAGCCTTCTCTGAACTGCGGCTCAGGCGGA

551 600

seq (551) GGTGTCACTCCTGCCTCCAGCCCAGGAGGGCAGGTCAGGTTATGCAACGC

LINC00941（040245 RACE） (410) GGTGTCACTCCTGCCTCCAGCCCAGGAGGGCAGGTCAGGTTATGCAACGC

601 650

seq (601) GTGCCGCGCGATCTCCCCCCACCTCCAACCCCCTTTTCTCCCGGGTCCAC

LINC00941（040245 RACE） (460) GTGCCGCGCGATCTCCCCCCACCTCCAACCCCCTTTTCTCCCGGGTCCAC

651 700

seq (651) ACCGCAGTTCCCACCGCTCCGGGTGTCCTCCCCAGTGCGCCGCGATTTTT

LINC00941（040245 RACE） (510) ACCGCAGTTCCCACCGCTCCGGGTGTCCTCCCCAGTGCGCCGCGATTTTT

701 750

seq (701) GTGTCCAAGCCCCAGAGTCCCTCTGAGACCAACCCCCAGCCAGCACAGAC

LINC00941（040245 RACE） (560) GTGTCCAAGCCCCAGAGTCCCTCTGAGACCAACCCCCAGCCAGCACAGAC

751 800

seq (751) TTCCTGCCTTCCCAGCTCGGGGATGTGGTCTCATTAGGTTGCCCAAGCTG

LINC00941（040245 RACE） (610) TTCCTGCCTTCCCAGCTCGGGGATGTGGTCTCATTAGGTTGCCCAAGCTG

801 850

seq (801) GACTTGTACTCTTGGCCTCAAGAGGTCCTTCCATCTCAGTCTCCCAAGTA

LINC00941（040245 RACE） (660) GACTTGTACTCTTGGCCTCAAGAGGTCCTTCCATCTCAGTCTCCCAAGTA

851 900

seq (851) GCTGGGACTACAAGCATGCACCACTACACTCAGCCAAATACTTTCAATAA

LINC00941（040245 RACE） (710) GCTGGGACTACAAGCATGCACCACTACACTCAGCCAAATACTTTCAATAA

901 950

seq (901) TTTGCCAGCTGACAACTTGATTGGGTTCTCCTTCAGGTTTGAAGCGCCCT

LINC00941（040245 RACE） (760) TTTGCCAGCTGACAACTTGATTGGGTTCTCCTTCAGGTTTGAAGCGCCCT

951 1000

seq (951) CGAGAAGTGTCTAAAGGAGACAGTTGATAGCCAAACAACAGTTTTGGATT

LINC00941（040245 RACE） (810) CGAGAAGTGTCTAAAGGAGACAGTTGATAGCCAAACAACAGTTTTGGATT

1001 1050

seq (1001) CACTGACTGATTATGAAAGAAGCAGTAGACTGGTATCAAGAATCAGTCAG

LINC00941（040245 RACE） (860) CACTGACTGATTATGAAAGAAGCAGTAGACTGGTATCAAGAATCAGTCAG

1051 1100

seq (1051) CAAGGAGGCCCTCACCAGACGCCAGTGCCATGTTCTTGGACTTCTCAGCC

LINC00941（040245 RACE） (910) CAAGGAGGCCCTCACCAGACGCCAGTGCCATGTTCTTGGACTTCTCAGCC

1101 1150

seq (1101) TCCATATTCATGAACTAAGTTTTTGGAATCCTTAGGCTTCCACGTGTGGA

LINC00941（040245 RACE） (960) TCCATATTCATGAACTAAGTTTTTGGAATCCTTAGGCTTCCACGTGTGGA

1151 1200

seq (1151) AAGCCTGAGCTAACCTACTGGAGGATGAGCCATCACCTGGAGCAGATTCA

LINC00941（040245 RACE） (1010) AAGCCTGAGCTAACCTACTGGAGGATGAGCCATCACCTGGAGCAGATTCA

1201 1250

seq (1201) GGCCATCCTAGTTGAAGCCTCCCTAGGCCAAGCAACCGTCCAACTACCAG

LINC00941（040245 RACE） (1060) GGCCATCCTAGTTGAAGCCTCCCTAGGCCAAGCAACCGTCCAACTACCAG

1251 1300

seq (1251) ACATTGACCATTCAGCCTTGAACATTCAGCACAAAGACAAAACAGACCAG

LINC00941（040245 RACE） (1110) ACATTGACCATTCAGCCTTGAACATTCAGCACAAAGACAAAACAGACCAG

1301 1350

seq (1301) ACCAGAAGAGTCCCACAGAATAGGGGAAACTATTCAGAGAAAACTTAAGC

LINC00941（040245 RACE） (1160) ACCAGAAGAGTCCCACAGAATAGGGGAAACTATTCAGAGAAAACTTAAGC

1351 1400

seq (1351) CACTAAGTTTTATGGTGTTTTGTTCTGTAGCAGAAGCATAGGCATACTGA

LINC00941（040245 RACE） (1210) CACTAAGTTTTATGGTGTTTTGTTCTGTAGCAGAAGCATAGGCATACTGA

1401 1450

seq (1401) CAATACAAACCGAAATCCTTCTAACGTAGTGGACCTTTTCAGGCCAGCAT

LINC00941（040245 RACE） (1260) CAATACAAACCGAAATCCTTCTAACGTAGTGGACCTTTTCAGGCCAGCAT

1451 1500

seq (1451) TTTTTCCTTGAAAACCTGGAGCATGTATCCATCTTATAGCAGAGATCACT

LINC00941（040245 RACE） (1310) TTTTTCCTTGAAAACCTGGAGCATGTATCCATCTTATAGCAGAGATCACT

1501 1550

seq (1501) TTCACAATGTTTGGGCTCTTGATTTGAATTGATGATGTAATGAGCCCTCT

LINC00941（040245 RACE） (1360) TTCACAATGTTTGGGCTCTTGATTTGAATTGATGATGTAATGAGCCCTCT

1551 1600

seq (1551) ATCCAGATTGTAACTAATTACTCTGCGAATTGACTGGATTCCACACCCTT

LINC00941（040245 RACE） (1410) ATCCAGATTGTAACTAATTACTCTGCGAATTGACTGGATTCCACACCCTT

1601 1650

seq (1601) CTAATATTTTACTTTTCCTCTTTTATCAACTCTCATTCTTGCTGCCATGA

```
LINC00941（040245 RACE）   (1460) CTAATATTTTACTTTTCCTCTTTTATCAACTCTCATTCTTGCTGCCATGA
                                  1651                                            1700
                     seq   (1651) TCAATGGACCAACTATGCTTATAACCACAAATTTTGATATGCTTAAACAA
LINC00941（040245 RACE）   (1510) TCAATGGACCAACTATGCTTATAACCACAAATTTTGATATGCTTAAACAA
                                  1701                                            1750
                     seq   (1701) ATGAACAAATATATTTAATAATTTCTTTTTTTTTTTTGAAATAGTATCTT
LINC00941（040245 RACE）   (1560) ATGAACAAATATATTTAATAATTTCTTTTTTTTTTTTGAAATAGTATCTT
                                  1751                                            1800
                     seq   (1751) GCTCTGTCACCCAGGCTGCAGTGCAGCAGCGTGATCTCAGCTCACTATAA
LINC00941（040245 RACE）   (1610) GCTCTGTCACCCAGGCTGCAGTGCAGCAGCGTGATCTCAGCTCACTATAA
                                  1801                                            1850
                     seq   (1801) CCTCCACCTCCCGGGTTCAAGTGATTCTCCTGCCTCAGCCTCCCAAGTAG
LINC00941（040245 RACE）   (1660) CCTCCACCTCCCGGGTTCAAGTGATTCTCCTGCCTCAGCCTCCCAAGTAG
                                  1851                                            1900
                     seq   (1851) CTGGGACTACAGGCGCCCACCACCATACCTGGCTAATTTTTTGTATTTTT
LINC00941（040245 RACE）   (1710) CTGGGACTACAGGCGCCCACCACCATACCTGGCTAATTTTTTGTATTTTT
                                  1901                                            1950
                     seq   (1901) AGTAGAGACAGGGTTTATCCATGTTGGCCAGGCTGGTCTCAAACTCCTGA
LINC00941（040245 RACE）   (1760) AGTAGAGACAGGGTTTATCCATGTTGGCCAGGCTGGTCTCAAACTCCTGA
                                  1951                                            2000
                     seq   (1951) CCTCAAGTGATCCTCCTGCCTCGGCCTCCCAAAGTGCTGGGATTACAGGT
LINC00941（040245 RACE）   (1810) CCTCAAGTGATCCTCCTGCCTCGGCCTCCCAAAGTGCTGGGATTACAGGT
                                  2001                                            2050
                     seq   (2001) GTGAGCCACCATGCCCAGCCAATAATTTCCTGATATAATAAAAATGCCAA
LINC00941（040245 RACE）   (1860) GTGAGCCACCATGCCCAGCCAATAATTTCCTGATATAATAAAAATGCCAA
                                  2051                                            2100
                     seq   (2051) TACTATACAATTAAATAGTAAAGTGATAAAAAAATAGGATAACATGATAAC
LINC00941（040245 RACE）   (1910) TACTATACAATTAAATAGTAAAGTGATAAAAAAATAGGATAACATGATAAC
                                  2101                                            2150
                     seq   (2101) CACTAATTCTCGAGTCTAGAGGGCCCGTTTAAACCCGCTGATCAGCCTCG
LINC00941（040245 RACE）   (1960) CACTAATT------------------------------------------
                                  2151                           2182
                     seq   (2151) ACTGTGCCTTCTAGTTGCCAGCCATCTGTTGT
LINC00941（040245 RACE）   (1968) --------------------------------
```

h-ELAVL1 (HuR) plasmid construction information:

The HuR plasmid vector was purchased from HanBio Biotechnology Company (Shanghai, China).

The map of pcdna3.1-ef1a-MCS-3flag-CMV-EGFP vector is as follows:

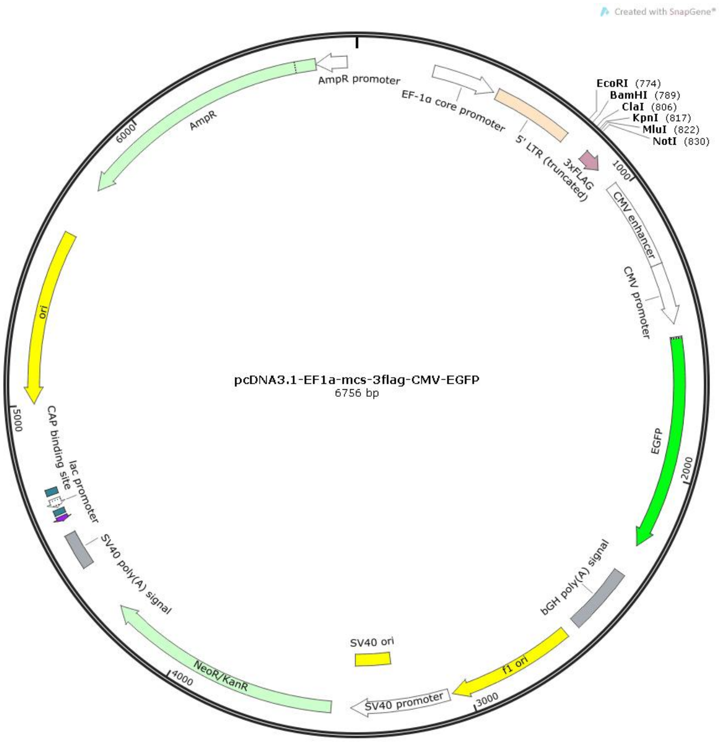

Gene sequence information:

Atgtctaatggttatgaagaccacatggccgaagactgcaggggtgacatcgggagaacgaatttgatcgtcaactacctc
cctcagaacatgacccaggatgagttacgaagcctgttcagcagcattggtgaagttgaatctgcaaaacttattcgggata
aagtagcaggacacagcttgggctatggctttgtgaactacgtgaccgcgaaggatgcagagagagcgatcaacacgct
gaacggcttgaggctccagtcaaaaaccattaaggtgtcgtatgctcgcccgagctcagaggtgatcaaagacgccaactt
gtacatcagcgggctcccgcggaccatgacccagaaggacgtagaagacatgttctctcggtttgggcggatcatcaactc
gcgggtcctcgtggatcagactacaggtttgtccagaggggttgcgtttatccggtttgacaaacggtcggaggcagaaga
ggcaattaccagtttcaatggtcataaacccccaggttcctctgagcccatcacagtgaagtttgcagccaaccccaaccag
aacaaaaacgtggcactcctctcgcagctgtaccactcgccagcgcgacggttcggaggccccgttcaccaccaggcgc
agagattcaggttctcccccatgggcgtcgatcacatgagcgggctctctggcgtcaacgtgccaggaaacgcctcctccg
gctggtgcattttcatctacaacctggggcaggatgccgacgaggggatcctctggcagatgtttgggccgtttggtgccgt
caccaatgtgaaagtgatccgcgacttcaacaccaacaagtgcaaagggtttggctttgtgaccatgacaaactatgaagaa
gccgcgatggccatagccagcctgaacggctaccgcctgggggacaaaatcttacaggtttccttcaaaaccaacaagtc
ccacaaataa

Primer design:

PC-h-ELAVL1-Eco/Bam-F	gctgtgaccggcgcctacgaattcGCCACCATGTCTAATGGTTAT
PC-h-ELAVL1-Eco/Bam-R	CccATCGATggACCGGTcgGGATCCTTTGTGGGACTTGTTGGTTTTG

The results of sequencing comparison are shown in the following figure: (the green area is the part that matches the target sequence)

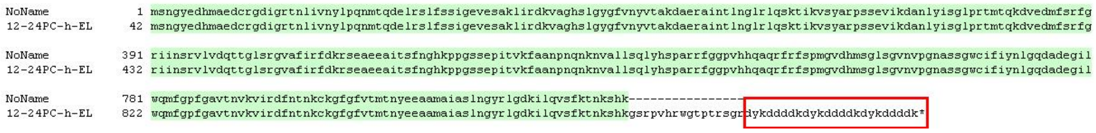

h-ELAVL1 △20-98aa (MUT1) plasmid construction information:

The h-ELAVL1 △20-98aa (MUT1) plasmid vector was purchased from HanBio Biotechnology Company (Shanghai, China).

The map of pcdna3.1-ef1a-MCS-3flag-CMV-EGFP vector is as follows:

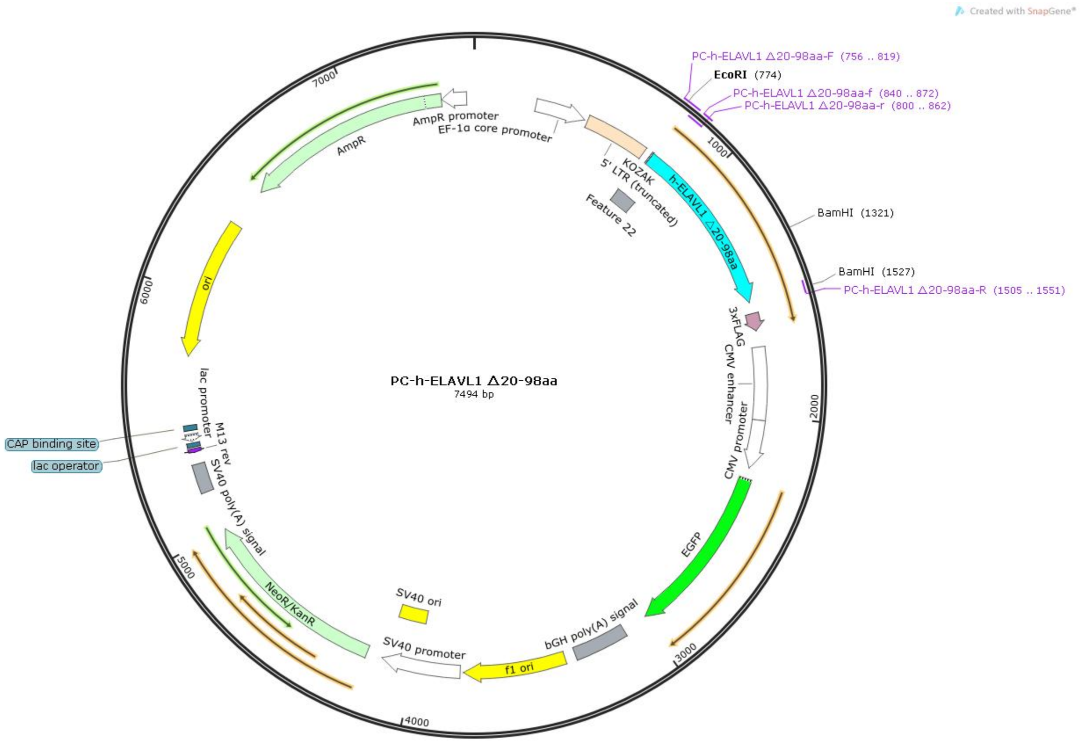

Gene sequence information:

ELAVL1 △20-98aa, with 3xFlag tag at the C terminal.

Atgtctaatggttatgaagaccacatggccgaagactgcaggggtgacatcgggagaagctcagaggtgatcaaagacg

ccaacttgtacatcagcgggctcccgcggaccatgacccagaaggacgtagaagacatgttctctcggtttgggcggatca

tcaactcgcgggtcctcgtggatcagactacaggtttgtccagaggggttgcgtttatccggtttgacaaacggtcggaggc
agaagaggcaattaccagtttcaatggtcataaacccccaggttcctctgagcccatcacagtgaagtttgcagccaacccc
aaccagaacaaaaacgtggcactcctctcgcagctgtaccactcgccagcgcgacggttcggaggccccgttcaccacc
aggcgcagagattcaggttctcccccatgggcgtcgatcacatgagcgggctctctggcgtcaacgtgccaggaaacgcc
tcctccggctggtgcattttcatctacaacctggggcaggatgccgacgaggggatcctctggcagatgtttgggccgtttg
gtgccgtcaccaatgtgaaagtgatccgcgacttcaacaccaacaagtgcaaagggtttggctttgtgaccatgacaaacta
tgaagaagccgcgatggccatagccagcctgaacggctaccgcctgggggacaaaatcttacaggtttccttcaaaacca
acaagtcccacaaa

Primer design:

PC-h-ELAVL1 △20-98aa-F	aagctgtgaccggcgcctacgaattcGCCACCAtgtctaatggttatgaagaccacatggccgaag
PC-h-ELAVL1 △20-98aa-r	tctttgatcacctctgagcttctcccgatgtcacccctgcagtcttcggccatgtggtcttca
PC-h-ELAVL1 △20-98aa-f	agaagctcagaggtgatcaaagacgccaacttg
PC-h-ELAVL1 △20-98aa-R	CccATCGATggACCGGTcgGGATCCTTTGTGGGACTTGTTGGTTTTG

The results of sequencing comparison are shown in the following figure: (the green area is the part that matches the target sequence)

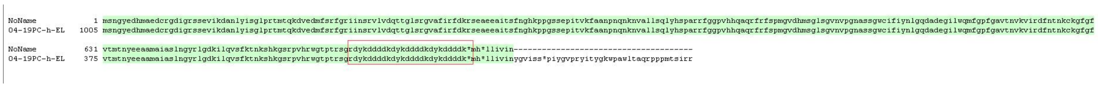

h-ELAVL1 △106-186aa (MUT2) plasmid construction information:

The h-ELAVL1 △106-186aa (MUT2) plasmid vector was purchased from HanBio Biotechnology Company (Shanghai, China).

The map of pcdna3.1-ef1a-MCS-3flag-CMV-EGFP vector is as follows:

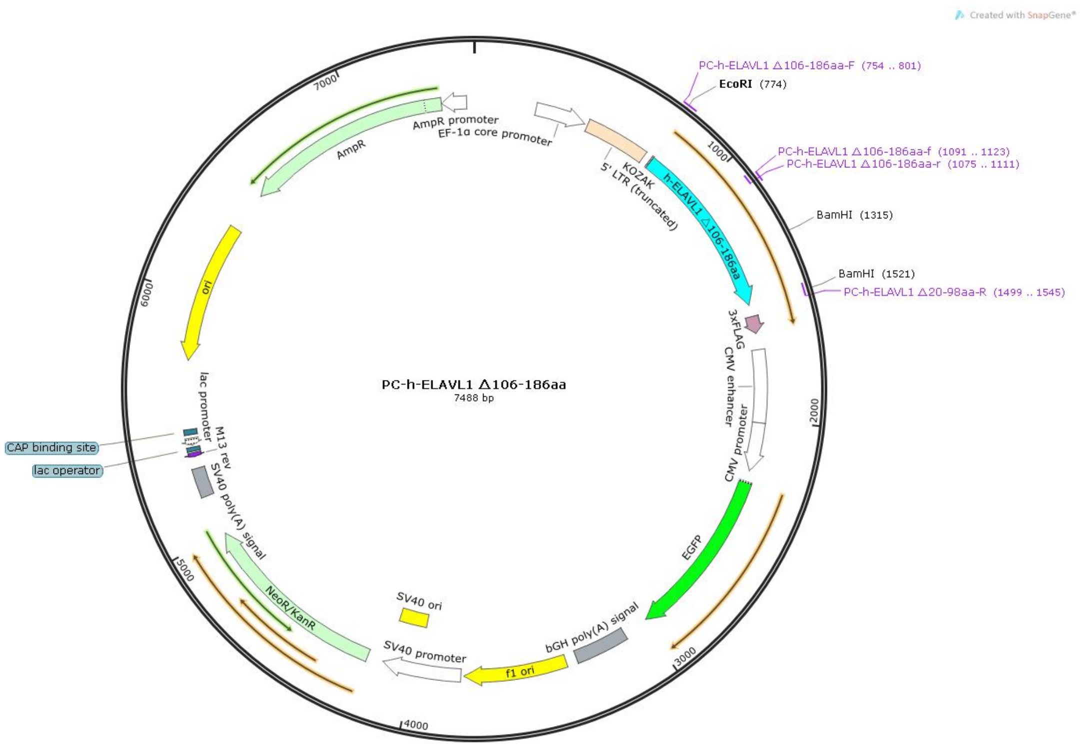

Gene sequence information:

ELAVL1 △106-186aa, C terminal plus 3xFlag tag.

atgtctaatggttatgaagaccacatggccgaagactgcaggggtgacatcgggagaacgaatttgatcgtcaactacctc

cctcagaacatgacccaggatgagttacgaagcctgttcagcagcattggtgaagttgaatctgcaaaacttattcgggata

aagtagcaggacacagcttgggctatggctttgtgaactacgtgaccgcgaaggatgcagagagagcgatcaacacgct

gaacggcttgaggctccagtcaaaaaccattaaggtgtcgtatgctcgcccgagctcagaggtgatcaaagaccccaacc

agaacaaaaacgtggcactcctctcgcagctgtaccactcgccagcgcgacggttcggaggccccgttcaccaccaggc

gcagagattcaggttctcccccatgggcgtcgatcacatgagcgggctctctggcgtcaacgtgccaggaaacgcctcctc

cggctggtgcattttcatctacaacctggggcaggatgccgacgaggggatcctctggcagatgtttgggccgtttggtgcc

gtcaccaatgtgaaagtgatccgcgacttcaacaccaacaagtgcaaagggtttggctttgtgaccatgacaaactatgaag

aagccgcgatggccatagccagcctgaacggctaccgcctgggggacaaaatcttacaggtttccttcaaaaccaacaag

tcccacaaa

Primer design:

PC-h-ELAVL1 △106-186aa-F	aagctgtgaccggcgcctacgaattcGCCACCatgtctaatggttatg
PC-h-ELAVL1 △106-186aa-r	ttctggttggggtctttgatcacctctgagctcgggc
PC-h-ELAVL1 △106-186aa-f	gatcaaagaccccaaccagaacaaaaacgtggc
PC-h-ELAVL1 △20-98aa-R	CccATCGATggACCGGTcgGGATCCTTTGTGGGACTTGTTGGTTTTG

The results of sequencing comparison are shown in the following figure: (the green area is the part that matches the target sequence)

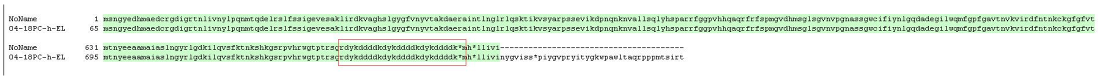

h-ELAVL1 △244-322aa (MUT3) plasmid construction information:

The h-ELAVL1 △244-322aa (MUT3) plasmid vector was purchased from HanBio Biotechnology Company (Shanghai, China).

The map of pcdna3.1-ef1a-MCS-3flag-CMV-EGFP vector is as follows:

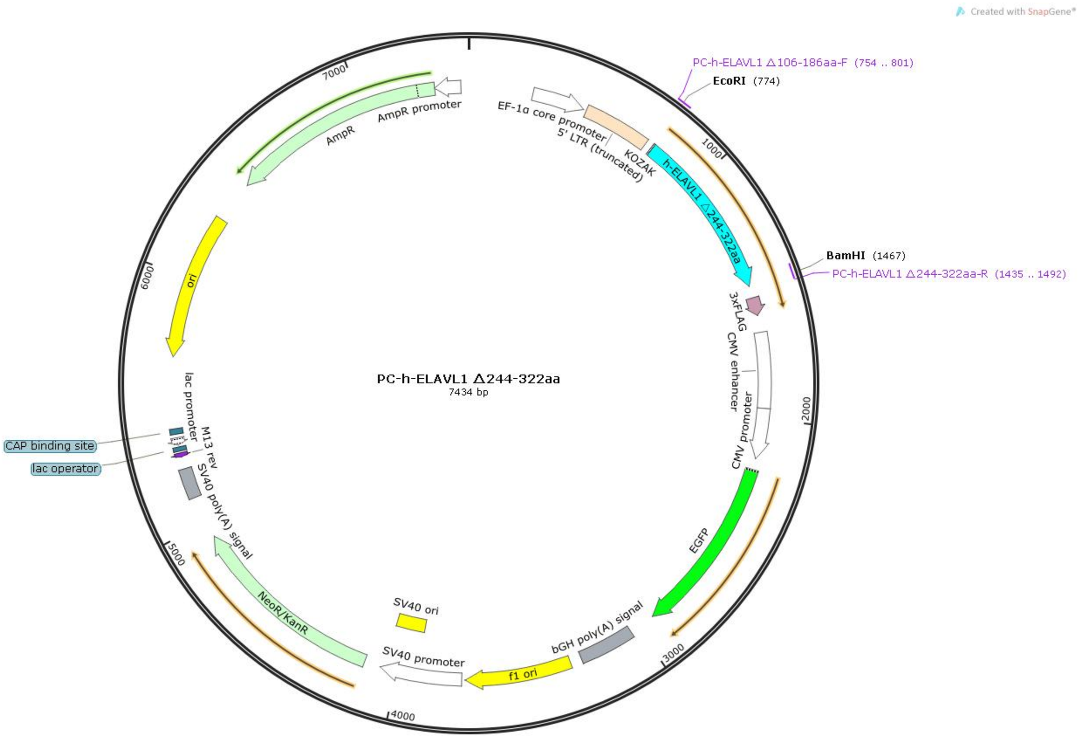

Gene sequence information:

ELAVL1 △244-322aa, C terminal plus 3xFlag tag.

Atgtctaatggttatgaagaccacatggccgaagactgcaggggtgacatcgggagaacgaatttgatcgtcaactacctc
cctcagaacatgacccaggatgagttacgaagcctgttcagcagcattggtgaagttgaatctgcaaaacttattcgggata
aagtagcaggacacagcttgggctatggctttgtgaactacgtgaccgcgaaggatgcagagagagcgatcaacacgct
gaacggcttgaggctccagtcaaaaaccattaaggtgtcgtatgctcgcccgagctcagaggtgatcaaagacgccaactt
gtacatcagcgggctcccgcggaccatgacccagaaggacgtagaagacatgttctctcggtttgggcggatcatcaactc
gcgggtcctcgtggatcagactacaggtttgtccagaggggttgcgtttatccggtttgacaaacggtcggaggcagaaga

ggcaattaccagtttcaatggtcataaacccccaggttcctctgagcccatcacagtgaagtttgcagccaaccccaaccag
aacaaaaacgtggcactcctctcgcagctgtaccactcgccagcgcgacggttcggaggccccgttcaccaccaggcgc
agagattcaggttctcccccatgaagtcccacaaa

Primer design:

PC-h-ELAVL1 △244-322aa-R	CCccATCGATggACCGGTcgGGATCCtttgtgggacttcatgggggagaacctgaatc
PC-h-ELAVL1 △106-186aa-F	aagctgtgaccggcgcctacgaattcGCCACCatgtctaatggttatg

The results of sequencing comparison are shown in the following figure: (the green area is the part that matches the target sequence)

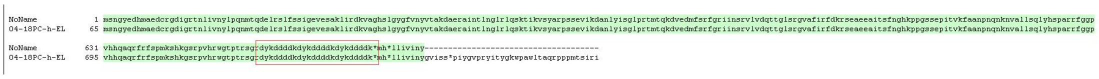

CXCL10 proomoter (WT) plasmid construction information:

The CXCL10 proomoter (WT) plasmid vector was purchased from Heyuan Biotechnology Company (Shanghai, China).

Clone serial number：H20837；GenBank ID：NR_040245.1; Gene size：2300

The map of pGL4.10-CXCL10 proomoter(WT) vector is shown in the following figure:

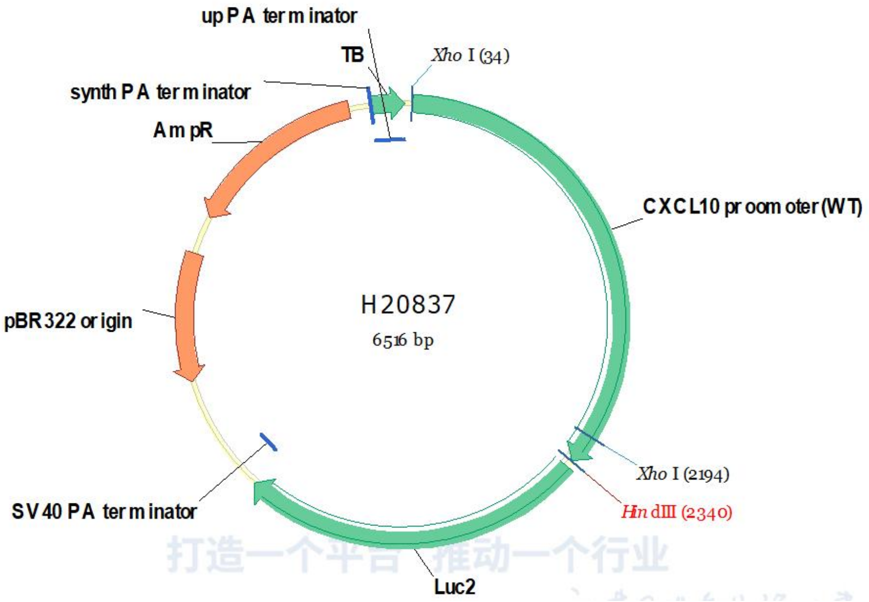

Primer design:

Forward sequencing primer:	RVprimer3 CTAGCAAAATAGGCTGTCCC
Reverse sequencing primer:	Luc2-N-R CGTCTTCGAGTGGGTAGAATG

Comparison of sequencing results:

```
                                1                                                50
                  SEQ     (1)   CATTTCTCTGGCCTAACTGGCCGGTACCTGAGCTCGCTAGCCTCGAGATA
CXCL10 proomoter(WT)     (1)   -----------------------------------------------ATA
                                51                                              100
                  SEQ    (51)   GAGTCATGGAGGGCTAAATCTAACTGGGCTTTCAATAGAAATTTATAATC
CXCL10 proomoter(WT)     (4)   GAGTCATGGAGGGCTAAATCTAACTGGGCTTTCAATAGAAATTTATAATC
                                101                                             150
                  SEQ   (101)   TGGAAGGTGGTGCAGGCTCACTTTAAGAAGGCTCACTCAGTGCCTTCATG
CXCL10 proomoter(WT)    (54)   TGGAAGGTGGTGCAGGCTCACTTTAAGAAGGCTCACTCAGTGCCTTCATG
                                151                                             200
                  SEQ   (151)   GTGGAATTGAGGAAACCCAGACCCAGGCAGCAACTCCCCAAGATTGCAGA
CXCL10 proomoter(WT)   (104)   GTGGAATTGAGGAAACCCAGACCCAGGCAGCAACTCCCCAAGATTGCAGA
                                201                                             250
                  SEQ   (201)   GATAGTAAGCAGTGAAGTTGGTATGCAAATCCAGGTCTTCTGAAGCCACA
CXCL10 proomoter(WT)   (154)   GATAGTAAGCAGTGAAGTTGGTATGCAAATCCAGGTCTTCTGAAGCCACA
                                251                                             300
                  SEQ   (251)   TCTGTAAGCCCCTCACTATATCACTACAACACCAGGGAGAGAGAGAGAGC
CXCL10 proomoter(WT)   (204)   TCTGTAAGCCCCTCACTATATCACTACAACACCAGGGAGAGAGAGAGAGC
                                301                                             350
                  SEQ   (301)   CCGGGGTTTCTTTCTCTTGAAAACGACCAGTCACTGGAGCAGAGCTGTTT
CXCL10 proomoter(WT)   (254)   CCGGGGTTTCTTTCTCTTGAAAACGACCAGTCACTGGAGCAGAGCTGTTT
                                351                                             400
                  SEQ   (351)   GCCCATGGAGATGAGTTTTATGTGAGCCAGGTTTACAAAGAGTAGAGTGT
CXCL10 proomoter(WT)   (304)   GCCCATGGAGATGAGTTTTATGTGAGCCAGGTTTACAAAGAGTAGAGTGT
                                401                                             450
                  SEQ   (401)   GGCTGGGCACGGTGGCTCACGCCTGTAATCCCAGCACTTTGGGAGGCCGA
CXCL10 proomoter(WT)   (354)   GGCTGGGCACGGTGGCTCACGCCTGTAATCCCAGCACTTTGGGAGGCCGA
                                451                                             500
                  SEQ   (451)   GGCAGGCAGATCACAAGGTCAGAAGATCAAGACCATCCTGGCTAACATGG
CXCL10 proomoter(WT)   (404)   GGCAGGCAGATCACAAGGTCAGAAGATCAAGACCATCCTGGCTAACATGG
                                501                                             550
                  SEQ   (501)   TGAAACCCCATCTCTACTAAAAAAATACCAAAAAATTAGCCAGGCGTGGT
CXCL10 proomoter(WT)   (454)   TGAAACCCCATCTCTACTAAAAAAATACCAAAAAATTAGCCAGGCGTGGT
                                551                                             600
                  SEQ   (551)   GGTGGGTGCCTGTAGTCCCAGCTACTTGGGAGGCTGAGGCAGGAGAAAGG
CXCL10 proomoter(WT)   (504)   GGTGGGTGCCTGTAGTCCCAGCTACTTGGGAGGCTGAGGCAGGAGAAAGG
                                601                                             650
                  SEQ   (601)   CTTGAACCCAGGAGGCGGAGCTTGCAGTGAGCTGAGATGGCACCACTGCG
CXCL10 proomoter(WT)   (554)   CTTGAACCCAGGAGGCGGAGCTTGCAGTGAGCTGAGATGGCACCACTGCG
```

```
                                     651                                              700
                  SEQ      (651)  CTCCAGCCTGGGTGACAGAGCGAGACTCTGTCAAAAAAAAAAAAAAGGGT
CXCL10 proomoter(WT)      (604)  CTCCAGCCTGGGTGACAGAGCGAGACTCTGTCAAAAAAAAAAAAAAGGGT
                                     701                                              750
                  SEQ      (701)  AGAGTAAGAAGAAAGAAGAGTGGAAGATTGCAGACAAATATGGCCAGCAA
CXCL10 proomoter(WT)      (654)  AGAGTAAGAAGAAAGAAGAGTGGAAGATTGCAGACAAATATGGCCAGCAA
                                     751                                              800
                  SEQ      (751)  GCATCTCTCTCCAGCCCTCTCTGGAGCCACGCTGTGCCCCTGCTCATGAC
CXCL10 proomoter(WT)      (704)  GCATCTCTCTCCAGCCCTCTCTGGAGCCACGCTGTGCCCCTGCTCATGAC
                                     801                                              850
                  SEQ      (801)  TTTCAGTTGCTTCACTATGCAATGAAAGTCAAAGCCCAGTTCCCATGCCA
CXCL10 proomoter(WT)      (754)  TTTCAGTTGCTTCACTATGCAATGAAAGTCAAAGCCCAGTTCCCATGCCA
                                     851                                              900
                  SEQ      (851)  CACACAGAGGAGTCCCTATCTCCTTTTCTGGCATCTTTTCTCACATTCCT
CXCL10 proomoter(WT)      (804)  CACACAGAGGAGTCCCTATCTCCTTTTCTGGCATCTTTTCTCACATTCCT
                                     901                                              950
                  SEQ      (901)  GCCACACAGATTCTCCACTCTAGCCACATACCTTATCCTGTCCCACCCAA
CXCL10 proomoter(WT)      (854)  GCCACACAGATTCTCCACTCTAGCCACATACCTTATCCTGTCCCACCCAA
                                     951                                             1000
                  SEQ      (951)  GGACCTTGGCTTCTACCATCCGGAAGTCTTCTGCCAACCCCAGGGCTGTC
CXCL10 proomoter(WT)      (904)  GGACCTTGGCTTCTACCATCCGGAAGTCTTCTGCCAACCCCAGGGCTGTC
                                     1001                                             1050
                  SEQ     (1001)  CAAATGCCTTTTCTTTTTCTTTTCTCTTTTTTTTTTTTTTTTTTTTTGGA
CXCL10 proomoter(WT)      (954)  CAAATGCCTTTTCTTTTTCTTTTCTCTTTTTTTTTTTTTTTTTTTTTGGA
                                     1051                                             1100
                  SEQ     (1051)  GACAGAGTTTTGCTCTCGTTGCCCAGGCTGGAGTGCAGTGGCGCCATCTT
CXCL10 proomoter(WT)     (1004)  GACAGAGTTTTGCTCTCGTTGCCCAGGCTGGAGTGCAGTGGCGCCATCTT
                                     1101                                             1150
                  SEQ     (1101)  GGCTGACTGCAACCTCCGCCTCCTGGGTTCAAGCGATTCTCCTGCCTCAG
CXCL10 proomoter(WT)     (1054)  GGCTGACTGCAACCTCCGCCTCCTGGGTTCAAGCGATTCTCCTGCCTCAG
                                     1151                                             1200
                  SEQ     (1151)  CCTCCCAAGTAGTTGGGATTACAGGTACCCACCACCACGCCCAGCTAATT
CXCL10 proomoter(WT)     (1104)  CCTCCCAAGTAGTTGGGATTACAGGTACCCACCACCACGCCCAGCTAATT
                                     1201                                             1250
                  SEQ     (1201)  TTTTGTATTTTTTTTAGCAGAGACAGGGTTTCATCGTGTTGGCCAGGCTG
CXCL10 proomoter(WT)     (1154)  TTTTGTATTTTTTTTAGCAGAGACAGGGTTTCATCGTGTTGGCCAGGCTG
                                     1251                                             1300
                  SEQ     (1251)  GCCAGGCTGGTCTGGAACTCCTGAAGTTAGGTGATCCACCCACCTCAGCC
CXCL10 proomoter(WT)     (1204)  GCCAGGCTGGTCTGGAACTCCTGAAGTTAGGTGATCCACCCACCTCAGCC
                                     1301                                             1350
                  SEQ     (1301)  CCTCAAAGTGCTGGGATTACAGGCGTTATCCACTGCGCCCCACCCCAGAT
CXCL10 proomoter(WT)     (1254)  CCTCAAAGTGCTGGGATTACAGGCGTTATCCACTGCGCCCCACCCCAGAT
                                     1351                                             1400
                  SEQ     (1351)  GCCTTTTCTATGAAGTATATCCCAGTTGGAAGTAATTTTTGTTTACCCTG
```

CXCL10 proomoter(WT) (1304) GCCTTTTCTATGAAGTATATCCCAGTTGGAAGTAATTTTTGTTTACCCTG

1401 1450

SEQ (1401) GATTTCTGTACATTCACTGATTGTGCAAAGAGCTTGCCACAGGCGGCCTT

CXCL10 proomoter(WT) (1354) GATTTCTGTACATTCACTGATTGTGCAAAGAGCTTGCCACAGGCGGCCTT

1451 1500

SEQ (1451) GCAAGATAGTTCTCTACCTTCAAGCCTTACTTCCTCCGAAATGAGTTAGG

CXCL10 proomoter(WT) (1404) GCAAGATAGTTCTCTACCTTCAAGCCTTACTTCCTCCGAAATGAGTTAGG

1501 1550

SEQ (1501) TCCCATGACATTAGAGCCTTCGGGATATTTCTGTACAGTTCTAGTTCCTA

CXCL10 proomoter(WT) (1454) TCCCATGACATTAGAGCCTTCGGGATATTTCTGTACAGTTCTAGTTCCTA

1551 1600

SEQ (1551) GCATAGCGCCTTGTATTCTTTAGGTACCTGACAAATGGCCACCAACCCTC

CXCL10 proomoter(WT) (1504) GCATAGCGCCTTGTATTCTTTAGGTACCTGACAAATGGCCACCAACCCTC

1601 1650

SEQ (1601) CACCGGAGAGGACTTTTTACCAAGCCTGCAGGAAGGGCCAGCTAGGCAGG

CXCL10 proomoter(WT) (1554) CACCGGAGAGGACTTTTTACCAAGCCTGCAGGAAGGGCCAGCTAGGCAGG

1651 1700

SEQ (1651) CGTCTCCCTGCTTTGCCCAACAGTGAAAGTCACAGCACATTTCTCAATAT

CXCL10 proomoter(WT) (1604) CGTCTCCCTGCTTTGCCCAACAGTGAAAGTCACAGCACATTTCTCAATAT

1701 1750

SEQ (1701) TATTCACGTTAAAAACATCTTTTTCCTGCTAAATAGACACACTCTCCACC

CXCL10 proomoter(WT) (1654) TATTCACGTTAAAAACATCTTTTTCCTGCTAAATAGACACACTCTCCACC

1751 1800

SEQ (1751) CTCTTTTAGTCAATCTTCCACAAAATTAGATAAGGAGAAATAACCCTTTG

CXCL10 proomoter(WT) (1704) CTCTTTTAGTCAATCTTCCACAAAATTAGATAAGGAGAAATAACCCTTTG

1801 1850

SEQ (1801) TTTCACATCCGCTGACTCAGATACGAGCATTATTCAACTGGGCGGGGGTG

CXCL10 proomoter(WT) (1754) TTTCACATCCGCTGACTCAGATACGAGCATTATTCAACTGGGCGGGGGTG

1851 1900

SEQ (1851) GCAGTGGATGGGAGGGGGCTGAGCAAACTACAAGTGGAGGAAGAAGAGGA

CXCL10 proomoter(WT) (1804) GCAGTGGATGGGAGGGGGCTGAGCAAACTACAAGTGGAGGAAGAAGAGGA

1901 1950

SEQ (1901) CAGCCTAGGAGAGGGAGGGCAGAAGAAAGGAGAGCACATTGTGCAGGGGC

CXCL10 proomoter(WT) (1854) CAGCCTAGGAGAGGGAGGGCAGAAGAAAGGAGAGCACATTGTGCAGGGGC

1951 2000

SEQ (1951) ACAGTGTTTTGCTGCTCCTTTTTTTCTTTCTTTTCTTAATATGGTCCTGG

CXCL10 proomoter(WT) (1904) ACAGTGTTTTGCTGCTCCTTTTTTTCTTTCTTTTCTTAATATGGTCCTGG

2001 2050

SEQ (2001) CTCACAGCAAGGCCACTACCTGACAAGCAGATGAGTCACTTCCTATGGGC

CXCL10 proomoter(WT) (1954) CTCACAGCAAGGCCACTACCTGACAAGCAGATGAGTCACTTCCTATGGGC

2051 2100

SEQ (2051) ACAGCGGGGGTACAACTTGATAGCTACAAAACCAGACAACCTCTCGCCAT

CXCL10 proomoter(WT) (2004) ACAGCGGGGGTACAACTTGATAGCTACAAAACCAGACAACCTCTCGCCAT

2101 2150

```
                 SEQ  (2101) GCGCTCACCCTGTTCCCAGACCCGGGTGGTGGTCTGGGGGGTTGGAAGGG
CXCL10 proomoter(WT)  (2054) GCGCTCACCCTGTTCCCAGACCCGGGTGGTGGTCTGGGGGGTTGGAAGGG
                                     2151                                          2200
                 SEQ  (2151) GCGGGAAGGGCGCTGTCTTAAGAATCATCCTCACATCTCAGGGCTTCGCC
CXCL10 proomoter(WT)  (2104) GCGGGAAGGGCGCTGTCTTAAGAATCATCCTCACATCTCAGGGCTTCGCC
                                     2201                                          2250
                 SEQ  (2201) CCTCGAGGGAGGTGACCCTTGGCCACCCTCTGCGCAGTTCTCCCTGAAAG
CXCL10 proomoter(WT)  (2154) CCTCGAGGGAGGTGACCCTTGGCCACCCTCTGCGCAGTTCTCCCTGAAAG
                                     2251                                          2300
                 SEQ  (2251) GTCATGCCACGTCTTAGCACTCTGGTCCCTGCGCCCCTGCAGGCCTCGCG
CXCL10 proomoter(WT)  (2204) GTCATGCCACGTCTTAGCACTCTGGTCCCTGCGCCCCTGCAGGCCTCGCG
                                     2301                                          2350
                 SEQ  (2301) GATCCCCCACTTGTCTCGACGCAGGGGACGCCCACTTGTCTGACGCAAAG
CXCL10 proomoter(WT)  (2254) GATCCCCCACTTGTCTCGACGCAGGGGACGCCCACTTGTCTGACGCA---
                                     2351                                          2400
                 SEQ  (2351) CTTGGCAATCCGGTACTGTTGGTAAAGCCACCATGGAAGATGCCAAAAAC
CXCL10 proomoter(WT)  (2301) --------------------------------------------------
```
